# Loss of killifish cGAS attenuates age-related signatures but does not affect organismal life span

**DOI:** 10.1038/s44319-026-00917-y

**Published:** 2026-09-09

**Authors:** Eugen Ballhysa, Roberto Ripa, Nadine Hochhard, Tin Tin Manh Nguyen, Jennifer Brazzell, Youngjun Park, Baptiste Ferreri, Elena Hoffmann, Raymond Laboy, Joachim David Steiner, Adam Antebi

**Affiliations:** 1 Max Planck Institute for Biology of Ageing, Cologne, Germany; 2 Cologne Excellence Cluster on Cellular Stress Responses in Aging-Associated Diseases (CECAD); University of Cologne, Cologne, Germany; 3 IDEXX BioAnalytics, Columbia, MO USA; 4 Sorbonne Université, Paris, France; 5 University of Cologne, Cologne, Germany; 6 Joslin Diabetes Center, Harvard Medical School, Boston, MA USA; 7 Department II of Internal Medicine and Center for Molecular Medicine Cologne, Faculty of Medicine and University Hospital Cologne, University of Cologne, Cologne, Germany

**Keywords:** Immunology, Molecular Biology of Disease, Signal Transduction

## Abstract

The cGAS/STING pathway is a central innate immune signaling pathway responsive to cytosolic DNA. Chronic activation of this pathway promotes numerous age-related pathologies, but its impact on lifespan remains unknown. Here we engineer a cGAS knockout (KO) in the turquoise killifish *Nothobranchius furzeri* to assess effects on physiology and aging. In cultured fibroblasts, cGAS deficiency results in elevated DNA damage but reduces radiation-induced senescence and enhances cellular proliferation. In vivo, cGAS KO attenuates DNA damage-induced transcriptional responses in young fish, and blunts age-associated transcriptional changes in old fish, consistent with dampening of senescence and aging. Accordingly, old cGAS KO animals exhibit lower levels of senescence-associated β-galactosidase activity and higher levels of cell proliferation, without detectable differences in immune infiltration. Despite these attenuated aging signatures, lifespan is not extended. Together, these findings reveal that while cGAS loss alleviates senescence and age-related signatures, additional mechanisms constrain longevity.

## Synopsis

Chronic activation of the cGAS/STING pathway promotes numerous age-related pathologies, but its impact on lifespan remains unknown. Loss of killifish cGAS results in an attenuation of irradiation induced senescence and age-related signatures but does not affect overall lifespan.

- Ablation of cGAS leads to increased intrinsic DNA damage and proliferation in killifish primary fibroblasts, yet decreased irradiation-induced senescence.
- Young cGAS KO fish have a blunted transcriptional response to irradiation-induced senescence.
- Old cGAS KO fish show attenuated age-related transcriptional signatures, reduced senescence and increased proliferation in a tissue-specific manner.
- cGAS KO and WT fish exhibit comparable lifespans.

## Abstract (web summary)

Chronic activation of the cGAS/STING pathway promotes numerous age-related pathologies, but its impact on lifespan remains unknown. Loss of killifish cGAS results in an attenuation of irradiation induced senescence and age-related signatures but does not affect overall lifespan.

## Graphical abstract

**Figure:**
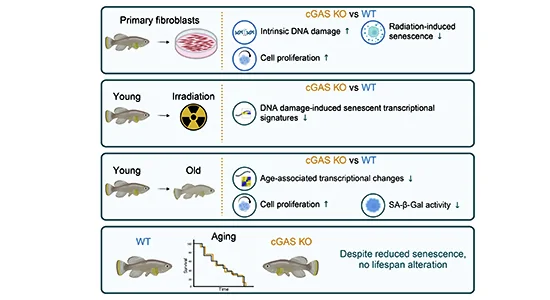

## Introduction

Aging is driven in part by inflammaging, a low-grade chronic inflammation that contributes to age-related pathology and loss of tissue homeostasis (Franceschi et al, 2000). Many age-related diseases, including cardiovascular (Yu et al, 2022), neurodegenerative (Baruch et al, 2014; Gulen et al, 2023), cancer (Samson and Ablasser, 2022), and metabolic diseases (Franceschi et al, 2018) are linked to unresolved inflammation, which impairs regenerative processes and promotes pathology.

A major source of inflammaging stems from chronic activation of the nucleic acid-sensing innate immune pathway cGAS/STING (Decout et al, 2021). Cytosolic DNA fragments arising from DNA damage (Gluck et al, 2017; Mackenzie et al, 2017; Yang et al, 2017), mitochondrial dysfunction (Sprenger et al, 2021; West et al, 2015), or infection (Du et al, 2023) bind cGAS (Civril et al, 2013) to stimulate production of 2’3’-cGAMP (cGAMP) (Sun et al, 2013; Wu et al, 2013). Subsequently, cGAMP activates STING, triggering the type I interferon response and inflammatory cytokine production (Ablasser et al, 2013). While this pathway is important for host defense, its chronic stimulation promotes age-related pathology (Zheng et al, 2023).

The cGAS-STING pathway also plays a central role in cellular senescence. Cells experiencing DNA damage (Dou et al, 2017; Yang et al, 2017), mitochondrial dysfunction (Victorelli et al, 2024; West et al, 2015), and other cellular insults (Gluck et al, 2017), permanently exit the cell cycle and secrete inflammatory cytokines, and matrix remodeling proteins, collectively termed the senescence-associated secretory phenotype (SASP) (Coppe et al, 2010). Cytosolic DNA in these cells is detected by cGAS, triggering the SASP, whose persistence fosters chronic inflammation (Khosla et al, 2020). Hence, limiting cGAS/STING pathway activity may improve late-life health. Indeed, late-life administration of STING inhibitors ameliorates neurodegeneration and slows cognitive decline in mice (Gulen et al, 2023).

Despite its importance in age-related disease, the effect of cGAS/STING on lifespan itself remains unknown. Here, we elucidated the role of cGAS in the short-lived African turquoise killifish *Nothobranchius furzeri*, a vertebrate model of aging. The *N. furzeri* GRZ strain lives only 4–8 months, yet recapitulates many features of mammalian aging (Boos et al, 2024; Kim et al, 2016; Platzer and Englert, 2016; Valdesalici and Cellerino, 2003). In this work, we used CRISPR-engineering to knock out killifish cGAS to examine its impact on senescence and aging. We hypothesized that cGAS loss would improve health and extend life. Although cGAS deficiency reduced senescence markers, attenuated age-related transcriptional changes, and maintained higher proliferative capacity with age, lifespan was unchanged, indicating that cGAS is not limiting for organismal longevity.

## Results and discussion

### cGAS structure and function are conserved from teleosts to mammals

**Figure 1:**
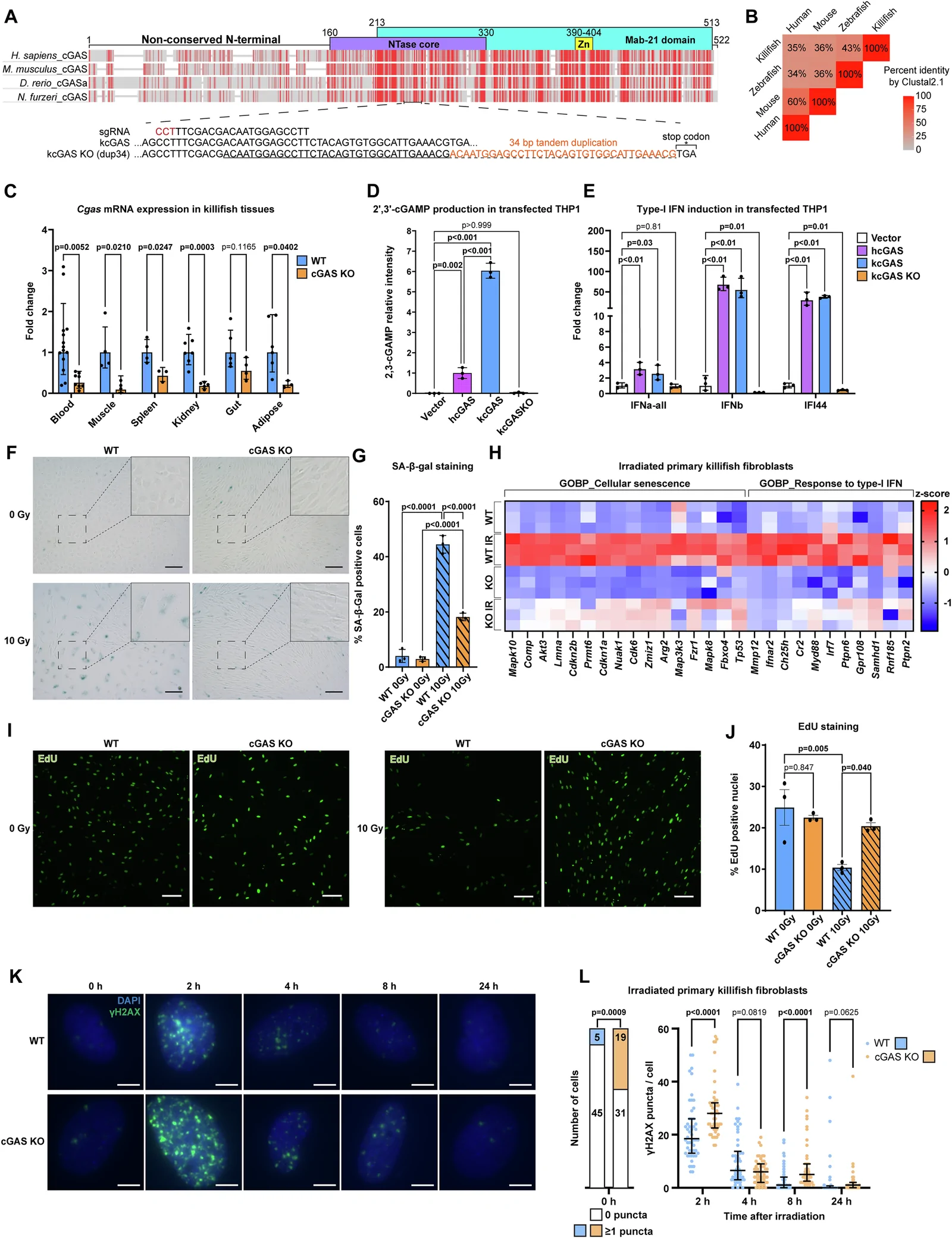
cGAS structure and function are conserved from teleosts to humans. (**A**) Multiple sequence amino acid alignment of killifish cGAS and orthologues from other species using a constraint-based multiple sequence alignment tool (COBALT). Identical amino acids are shown in red, non-identical in gray. NTase, Mab21, and Zn finger domains are shown above the alignment. Schematic of the CRISPR-Cas9-generated mutated genomic region of killifish cGAS and the sgRNA used is shown below. (**B**) Triangle heatmap showing pairwise percent identity between aligned cGAS orthologs, calculated using ClustalW. (**C**) cGAS mRNA expression levels were measured by qPCR of different tissues from WT and kcGAS KO animals. Each dot represents data from one fish. n ≥ 3 fish per tissue and genotype. Normalized expression values of *Cgas* to *Gapdh* were statistically analyzed using the Student’s *t* test. Error bars indicate standard deviation from the mean. (**D**) LC–MS-based targeted metabolomics of 2’,3’-cGAMP extracted from THP1 cells transfected with human cGAS (hcGAS), killifish cGAS (kcGAS), and mutated killifish cGAS (kcGAS KO). All values were normalized to hcGAS levels to depict fold change from the hcGAS average. Statistical analysis was performed using one-way ANOVA with Dunn–Šidák correction for multiple comparisons. Exact *P* values for the comparisons shown are: Vector vs hcGAS, *P* = 0.002679308676358; Vector vs kcGAS, *P* = 0.000000003578504; Vector vs kcGAS KO, *P* = 0.999911579750400; hcGAS vs. kcGAS, *P* = 0.000000015056732. Error bars indicate standard deviation from the mean. (**E**) qPCR analysis of interferon and interferon-stimulated genes from cell extracts of cGAS KO THP1 cells transfected with the indicated cGAS genes. Samples correspond to those used in (**D**). Expression values normalized to GAPDH were statistically analyzed using the Student’s *t* test. Exact *P* values for the comparisons shown are: For IFNa-all; Vector vs hcGAS, *P* = 0.008737317; Vector vs kcGAS, *P* = 0.030516546; Vector vs kcGAS KO, *P* = 0.8128233, For IFNb; Vector vs hcGAS, *P* = 0.001134425; Vector vs kcGAS, *P* = 0.00182603; Vector vs kcGAS KO, *P* = 0.016581201, For IFI44; Vector vs hcGAS, *P* = 0,000644174; Vector vs kcGAS, *P* = 3.72803E-05; Vector vs kcGAS KO, *P* = 0.013859455. Error bars indicate standard deviation from the mean. (**F**) Bright-field images of killifish WT and cGAS KO primary fibroblasts stained for SA-β-galactosidase activity 9 days after exposure to 10 Gy γ-radiation. Scale bar = 100 μm. (**G**) Quantitation of SA-β-galactosidase-positive cells from images in (**F**). At least 100 cells were quantified per replicate. Statistical analysis was performed using one-way ANOVA with Dunn–Šidák correction for multiple comparisons. Exact *P* values for the comparisons shown are: WT 0 Gy vs WT 10 Gy, *P* = 0.000000039453939; WT 10 Gy vs cGAS KO 10 Gy, *P* = 0.000001147611475; cGAS KO 0 Gy vs cGAS KO 10 Gy, *P* = 0.000071001144959. Error bars indicate standard deviation from the mean. (**H**) Heatmaps showing the z-score-normalized expression values of senescence and type-I interferon response genes from RNAseq analysis of irradiated and non-irradiated WT and cGAS KO primary fibroblasts, highlighting cGAS-dependent regulation. Shown are the top 16 senescence and top 11 type-I interferon response genes upregulated in irradiated WT compared to non-irradiated WT cells. (**I**) Representative images of primary killifish fibroblasts stained for EdU (Green) 9 days after irradiation. Cells were incubated with EdU for 4 h prior to fixation and staining. Scale bar = 100 μm. (**J**) Quantification of EdU-positive cells from images in (**I**). Error bars indicate standard deviation from the mean. (**K**) γH2AX (Green) and DAPI (blue) staining of WT and cGAS KO primary fibroblasts fixed at the indicated times following 10 Gy of γ-radiation. Non-irradiated cells correspond to 0 h controls. These representative images are zoomed in to show individual cells, while the source images have a wider view with multiple cells per image. Scale bar = 20 μm. (**L**) Quantitation of phosphorylated γH2AX puncta from (**K**). For the 0 h timepoint, bars indicate the number of cells displaying either zero or at least one γH2AX puncta. For later timepoints, each dot represents the number of puncta per cell. *n* ≥50 cells were measured for each condition. Statistical analysis was performed using the Mann–Whitney test. Exact *P* values for the comparisons shown are: WT 0 h vs KO 0 h, *P* = 0.000904937458743; WT 2 h vs KO 2 h, *P* = 0.000042564777741; WT 4 h vs KO 4 h, *P* = 0.081927997835422; WT 8 h vs KO 8 h, *P*= 0.000001866827479; WT 24 h vs KO 24 h, *P* = 0.062464600371320. Error bars indicate standard deviation from the mean. Data information: All cell culture experiments in (**D**–**L**) were performed in three independent biological replicates with *n* = 3 plates per experiment. Each panel shows one representative experiment. Source data are available online for this figure.

**Figure EV1:**
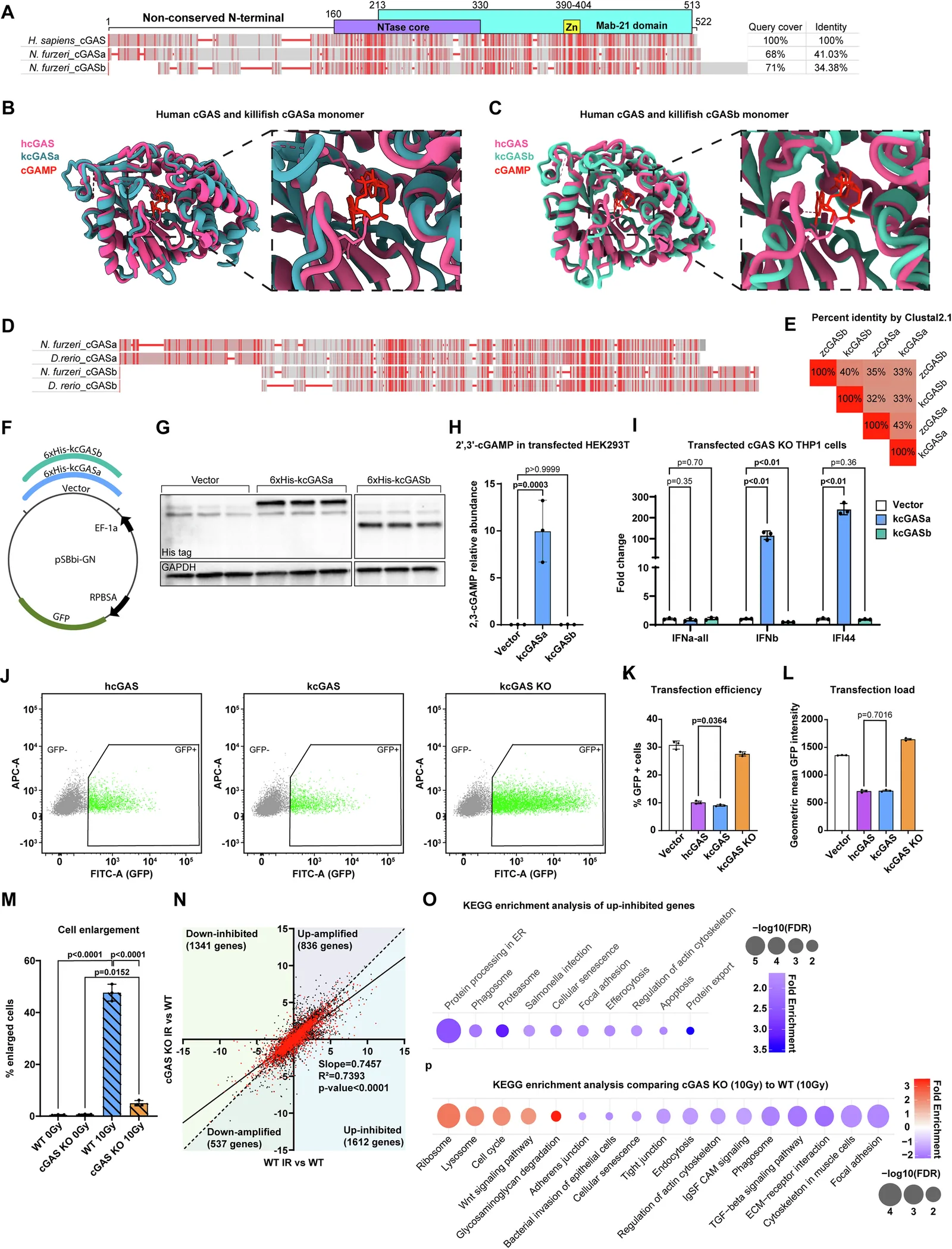
Killifish and human cGAS have similar tertiary structures, and kcGASa harbors cGAMP activity. (**A**) Multiple sequence amino acid alignment of human cGAS with killifish cGASa and cGASb generated using COBALT. Identical amino acids are shown in red, non-identical in gray. NTase, Mab21, and Zn finger domains are shown above the alignment.Right, percent identity to human cGAS as measured by BLAST. (**B**) Structural alignment of the human cGAS (PDB 5VDO) with the AlphaFold 3-predicted model of killifish cGASa bound to cGAMP. Inset depicts the cGAMP-binding pocket. (**C**) Structural alignment of the human cGAS (pdb 5vdo) with the AlphaFold 3-predicted model of killifish cGASb, while bound to cGAMP. Inset depicts the cGAMP-binding pocket. (**D**) Multiple sequence amino acid alignment of cGASa and cGASb orthologs from killifish and zebrafish was generated using COBALT. Identical amino acids are shown in red, non-identical in gray. (**E**) Triangle heatmap showing pairwise percent identity between aligned cGAS proteins in (**D**), calculated using ClustalW. (**F**) Schematic of expression vectors used for transfection of 6xHis-tagged kcGASa and kcGASb into THP1 and HEK293T cells. cGAS is driven by the *Ef-1a* promoter, and GFP driven by the *Rpbsa* promoter. Plasmid maps and sequences are provided in the Reagents & Tools Table. (**G**) Western blot analysis of HEK293T cell lysates transfected with empty expression vector or with plasmids harboring 6xHis-tagged killifish cGASa, killifish cGASa KO null mutation, and killifish cGASb. Blots were probed with anti-6xHis tag and anti-GAPDH as an internal control. (**H**) LC–MS-based targeted metabolomics of 2’,3’-cGAMP from cell extracts shown in (**G**). cGAMP levels per tissue were normalized to protein concentrations measured by BCA. Statistical analysis was performed using one-way ANOVA with Tukey correction for multiple comparisons. Error bars indicate standard deviation from the mean. (**I**) qPCR analysis of interferon and interferon-stimulated genes from cell extracts of cGAS KO THP1 cells transfected with the indicated cGAS constructs. Samples are identical to those used in (**H**). Expression values normalized to GAPDH were statistically analyzed using the Student’s *t* test. Exact *P* values for the comparisons shown are: For IFNa-all; Vector vs kcGASa, *P* = 0.353092; Vector vs kcGASb, *P* = 0.699750, for IFNb; Vector vs kcGASa, *P* = 0.000537; Vector vs kcGASb, *P* = 0.000988, for IFI44; Vector vs kcGASa, *P* = 0.000115; Vector vs kcGASb, *P* = 0.359836. Error bars indicate standard deviation from the mean. (**J**) Representative flow cytometry plots of live THP1 cells transfected with plasmids carrying the indicated cGAS genes. The GFP intensity on the *x* axis (FITC-A) is plotted against background noise (APC-A) on the *y* axis. GFP + -gated cells are considered successfully transfected. (**K**) Quantification of transfection efficiency derived from the GFP+ gate as shown in (**J**). Student’s *t* test was used to compare hcGAS and kcGAS means. Error bars indicate standard deviation from the mean. (**L**) Geometric means of GFP intensities of the GFP+ cells shown in (**J**). Student’s *t* test was used to compare hcGAS and kcGAS geometric means. Error bars indicate standard deviation from the mean. (**M**) Percent of cells with area greater than 3000 μm^2^ in irradiated and non-irradiated primary killifish fibroblasts. At least 500 cells were quantified per replicate. Statistical analysis was performed using One-way ANOVA with Dunn–Šidák correction for multiple comparisons. Exact *P* values for the comparisons shown are: WT 0 Gy vs WT 10 Gy, *P* = 0.000000001902642; KO 0 Gy vs KO 10 Gy, *P* = 0.015185213159004; WT 10 Gy vs KO 10 Gy, *P* = 0.000000002802417. Error bars indicate standard deviation from the mean. (**N**) Linear regression analysis of the log2-Fold Change (Log2FC) of genes significantly regulated by cGAS during irradiation identified by gene_~_irradiation interaction analysis. The *x* axis represents the Log2FC between irradiated and non-irradiated WT fibroblasts, and the *y* axis represents the Log2FC between irradiated cGAS KO and non-irradiated WT fibroblasts. The solid line indicates the linear regression fit of the significantly regulated genes in gene_~_irradiation interaction, while the dashed line represents the reference slope = 1 (*x* = *y*). (**O**) KEGG pathway enrichment of the up-inhibited genes in (**N**). Genes in this category are upregulated by irradiation in WT fibroblasts, but cGAS loss significantly blunts upregulation. All significantly (FDR < 0.05) regulated KEGG pathways are shown. (**P**) KEGG pathway enrichment comparing irradiated cGAS KO to irradiated WT primary fibroblasts. All significantly (FDR < 0.05) regulated KEGG pathways are shown. Data information: Flow cytometry data in (**J**–**L**) represent the transfection experiments shown in Fig. 1D,E. Cell enlargement data in (**M**) represent the irradiation experiment shown in Fig. 1F,G. Experiments were performed with three independent biological replicates, *n* = 3 plates per replicate, except for HEK293T transfections in (**G**, **H**), which were performed with two independent biological replicates, and transfection of THP1 cells for immune activation in (**I**), which was performed once. Each panel shows one representative experiment. IR irradiated, FDR false discovery rate.

BLAST analysis of the *N. furzeri* genome (UI_Nfuz_MZM_1.0 reference genome) revealed two genes homologous to the human cGAS, which we termed cGASa (XP_015800200) and cGASb (XP_054600933.2). cGASa showed greater amino acid identity with human cGAS (Figs. 1A,B and EV1A) and was more similar in protein length (kcGASa 560aa; kcGASb 472 aa; hcGAS 522 aa). Both homologs retained the conserved catalytic domains, including the NTase core that catalyzes cGAMP production and the Mab21 domain that binds DNA. However, kcGASa had higher tertiary structural similarity to hcGAS than kcGASb (RMSD: 0.944 Å for kcGASa versus 1.191 Å for kcGASb), and preserved key hydrogen-bonding interactions with cyclic nucleotides absent in kcGASb (Fig. EV1B,C).

Killifish cGASb is most homologous to zebrafish cGASb (Fig. EV1D,E), which exhibits minimal cGAS activity (Liu et al, 2020). To assess cyclic GMP-AMP synthase activity of the two killifish cGAS paralogs, we expressed them in cGAS-deficient HEK293T cells (Sun et al, 2013). Despite comparable protein expression (Fig. EV1F,G), only kcGASa generated measurable intracellular cGAMP (Fig. EV1h). Furthermore, whereas plasmid transfection with kcGASa robustly activated innate immune gene expression, kcGASb did not (Fig. EV1i). Thus, kcGASa appears to be the functional ortholog, and hereafter we refer to it as killifish cGAS (kcGAS).

To investigate kcGAS physiologic function, we generated a CRISPR/Cas9 knock-out. The sgRNA caused random mutagenesis, resulting in a 34 bp tandem duplication that led to an early stop codon, creating a presumptive null (Fig. 1A) (Annibal et al, 2021). Consistently, kcGAS mRNA expression was reduced across multiple tissues, implying nonsense-mediated RNA decay (Fig. 1C), and KO animals completely lacked measurable cGAMP (Annibal et al, 2021). To estimate intrinsic activity of hcGAS versus kcGAS, we transfected wild-type hcGAS, kcGAS, and mutated kcGAS (kcGAS KO, same mutation as above), into cGAS-deficient human THP1 cells. High levels of cGAMP were detected in cells expressing human and killifish cGAS, whereas empty vector or kcGAS KO controls produced no detectable cGAMP (Fig. 1D), confirming our in vivo observations. Notably, kcGAS generated higher levels of cGAMP than hcGAS, suggesting elevated intrinsic activity similar to mouse cGAS (Zhou et al, 2018). Accordingly, kcGAS showed lower transfection efficiency than hcGAS or kcGAS KO (Fig. EV1J,K)—likely reflecting activation of the cytosolic DNA response, despite similar transgene load (Fig. EV1L). Finally, only enzymatically active constructs induced innate immune gene expression (Fig. 1E). Induction levels were comparable between hcGAS and kcGAS, likely due to signal saturation (Fig. 1E).

Cellular senescence is closely linked to the aging process. In mammalian cell culture, cGAS helps establish multiple aspects of the senescent phenotype (Dou et al, 2017; Gluck et al, 2017; Gulen et al, 2023; Yang et al, 2017). To test whether this function is conserved, we isolated primary fibroblasts from WT and cGAS KO killifish fins, and exposed them to 0 Gy or 10 Gy of γ-radiation to induce DNA damage-driven senescence. Nine days after irradiation, cGAS KO fibroblasts retained a spindle-like morphology, had significantly fewer enlarged cells (Fig. EV1M) and senescence-associated β-galactosidase (SA-β-gal) positive cells compared to WT fibroblasts (Fig. 1F,G), indicating reduced senescence. Consistent with these findings, transcriptome analysis showed that expression of multiple senescence-associated genes (e.g., *Cdkn2b*, *Cdkn1a*, *Lmna*) and interferon pathway genes (e.g., *Mmp12*, *Ifnar2*, *Irf7*) was attenuated (Fig. 1H). Gene _~_ irradiation interaction analysis (Duda et al, 2023) revealed that the regulation of hundreds of transcripts was significantly blunted in response to irradiation (WT IR vs WT) in cGAS KO cells (Fig. EV1N). Up-inhibited genes subjected to KEGG enrichment revealed pathways including senescence and apoptosis (Fig. EV1N,O), whereas down-inhibited genes were associated with protein synthesis (ribosome), cell–cell adhesion, and canonical developmental signaling (Notch and Wnt), suggesting a shift in growth, proliferation, and cell fate signaling. Pairwise comparison of irradiated cGAS KO cells to irradiated WT controls yielded similar results. Significantly regulated genes subjected to KEGG enrichment showed reduced senescence and increased cell cycle programs in cGAS KO fibroblasts (Fig. EV1P). Accordingly, irradiated cGAS KO cells maintained high levels of cell proliferation, measured by EdU incorporation, whereas cell proliferation markedly declined in irradiated WT controls (Fig. 1I,J). Altogether, these data indicate that loss of killifish cGAS dampens multiple features of cellular senescence.

DNA damage is intimately linked to aging, and cGAS plays an important role in genomic maintenance. Mouse and human cGAS exacerbate DNA damage during genotoxic stress (Jiang et al, 2019; Liu et al, 2018), whereas naked mole rat cGAS protects against DNA damage (Chen et al, 2025). To investigate whether killifish cGAS influences this process, we irradiated WT and killifish cGAS KO primary fibroblasts and quantified γH2AX puncta as a proxy for DNA damage at several time points (0, 2, 4, 8, and 24 h). Interestingly, even in the absence of irradiation (0 h), kcGAS KO fibroblasts contained significantly more γH2AX puncta than WT cells (Fig. 1K,L). At 2 h post-irradiation, γH2AX puncta peaked at higher levels in kcGAS KO cells, and persisted over the next few hours before returning to basal levels 24 h post-irradiation. This finding suggests that killifish cGAS may normally protect against DNA damage, similar to naked mole rat cGAS. In summary, despite elevated DNA damage signaling, cGAS KO fibroblasts exhibit reduced senescence and immune marker activation, and increased DNA replication in vitro, suggesting relaxed cell cycle control.

### Killifish cGAS impacts innate immunity regulation in vivo

**Figure 2:**
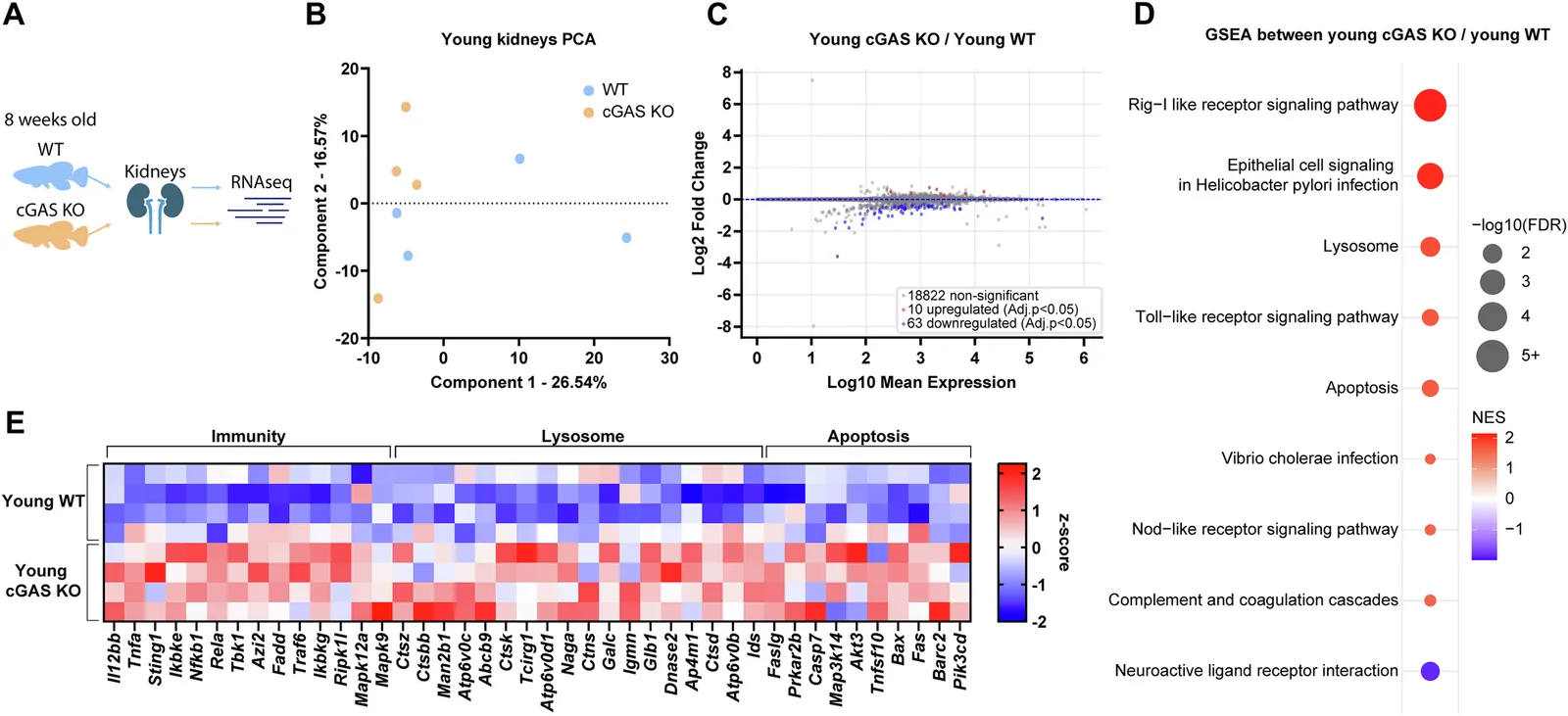
Loss of cGAS stimulates upregulation of downstream innate immune signaling components in young killifish. (**A**) Schematic overview depicting samples used for RNAseq analysis. Transcriptomic profiling was performed on kidney RNA from young 8-week-old WT and cGAS KO killifish. (**B**) Principal component analysis (PCA) of kidney transcriptomes from young WT and cGAS KO animals. (**C**) MA plot showing minimal differential gene expression comparing kidney transcriptomes from young cGAS KO and WT animals. Genes with adjusted *P* value < 0.05 were considered significantly differentially expressed and marked as upregulated (red) or downregulated (blue). (**D**) GSEA comparing kidney transcriptomes from young cGAS KO and WT animals. All significantly (FDR < 0.05) up- and downregulated KEGG pathways are shown. Unless noted otherwise, gene sets throughout the paper use the KEGG_LEGACY subset of canonical pathways. Additional KEGG pathways and associated genes can be found in our data repository (see Source Data and “Data availability”). (**E**) Heatmap of z-score normalized expression values of the genes with the highest positive and lowest negative rank metric scores from representative GSEA pathways shown in (**D**). Data information: NES normalized enrichment score, FDR false discovery rate. *N* = 4 fish per genotype. Some elements of the illustration in (**A**) were created with biorender.com. Source data are available online for this figure.

**Figure EV2:**
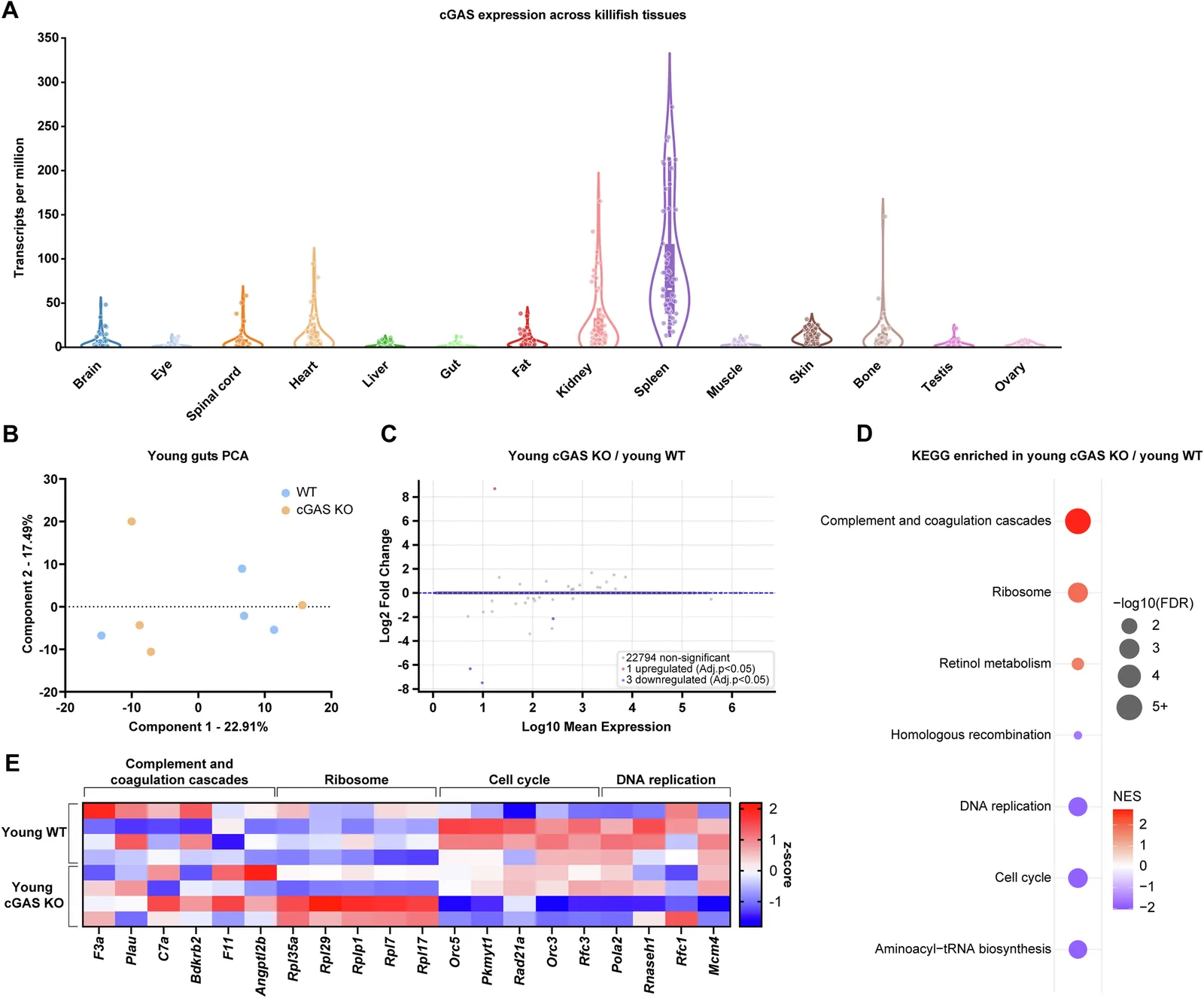
Lack of cGAS minimally impacts the young gut transcriptome. (**A**) Violin plot showing cGAS gene expression across killifish tissues. Expression data were obtained from Costa E et al (Costa et al, 2026). Exact values for each tissue are: for brain, median = 1.6245286431846302, Q1 = 1.0791615694676375, Q3 = 2.8037888982471975, IQR = 1.72462732877956, lower whisker=0.474158421926774, upper whisker = 5.390729891416537, min=0.474158421926774, max = 48.2453715667804; for eye, median=0.547680269358157, Q1 = 0.235363777736343, Q3 = 0.798345855574392, IQR = 0.562982077838049, lower whisker = 0.0, upper whisker=1.6428189723314655, min = 0.0, max = 12.1157552945876; for spinal cord, median=2.04627555126626, Q1 = 1.284645037250495, Q3 = 3.4376155205063776, IQR = 2.1529704832558827, lower whisker=0.217740281324982, upper whisker=6.6670712453902015, min = 0.217740281324982, max = 58.4880278758347; for heart, median=3.865281330013805, Q1 = 1.9589985254247875, Q3 = 12.811329526353475, IQR = 10.852331000928688, lower whisker = 0.439708512712193, upper whisker=29.089826027746508, min=0.439708512712193, max=94.5804438771201; for liver, median=0.5173101940333855, Q1 = 0.1746531946679195, Q3 = 2.0735977679541873, IQR = 1.8989445732862678, lower whisker = 0.0, upper whisker = 4.9220146278835895, min = 0.0, max = 10.8698616012988; for gut, median = 0.747865822726491, Q1 = 0.3397524591959925, Q3 = 1.795310461933685, IQR = 1.4555580027376926, lower whisker=0.0498437438811976, upper whisker=3.978647466040224, min=0.0498437438811976, max=11.8289638252401; for fat, median=3.01517811785425, Q1 = 1.73249885639874, Q3 = 5.46268366311472, IQR = 3.7301848067159797, lower whisker = 0.205501559850421, upper whisker=11.05796087318869, min = 0.205501559850421, max=38.2113117279423; for kidney, median=10.8054617219689, Q1 = 6.9128429676001595, Q3 = 27.6433653947586, IQR = 20.73052242715844, lower whisker=2.39874603857986, upper whisker=58.73914903549626, min=2.39874603857986, max=165.549858564364; for spleen, median=65.9689996898086, Q1 = 43.244477891429796, Q3 = 111.433045617879, IQR = 68.18856772644921, lower whisker = 13.3715532974671, upper whisker=213.7158972075528, min = 13.3715532974671, max = 271.947822176741; for muscle, median = 1.79864845535314, Q1 = 1.299485437079505, Q3 = 2.75280808943001, IQR = 1.453322652350505, lower whisker = 0.19207068303625, upper whisker = 4.932792067955767, min = 0.19207068303625, max=11.5862018603163; for skin, median = 11.123886655218449, Q1 = 6.916782891220315, Q3 = 16.5269339270682, IQR = 9.610151035847885, lower whisker=3.53813755557935, upper whisker=30.942160480840027, min=3.53813755557935, max=31.7439057910779; for bone, median=5.51303497371631, Q1 = 3.956933329710515, Q3 = 10.54572843574095, IQR = 6.588795106030434, lower whisker=1.2402779123645, upper whisker=20.428921094786602, min=1.2402779123645, max=148.035881907591; for testis, median=1.37947155752431, Q1 = 1.04091472081611, Q3 = 3.669744486008645, IQR = 2.6288297651925348, lower whisker=0.262387918868094, upper whisker = 7.612989133797447, min = 0.262387918868094, max = 21.4040638076684; for ovary, median=2.35800766871269, Q1 = 0.7814855668306304, Q3 = 3.962219821641135, IQR = 3.1807342548105044, lower whisker = 0.110071398308551, upper whisker=6.8056434046692, min=0.110071398308551, max = 6.8056434046692; (**B**) PCA of gut transcriptomes from young 8-week-old WT and cGAS KO animals. (**C**) MA plot showing minimal differential gene expression comparing gut transcriptomes from young cGAS KO to WT animals. Genes with adjusted *P* value < 0.05 were considered significantly differentially expressed and marked as upregulated (red) or downregulated (blue). (**D**) GSEA comparing gut transcriptomes from young cGAS KO to WT animals. All significantly (FDR < 0.05) up- and downregulated KEGG pathways are shown (**E**). Heatmap showing z-score-normalized expression values of genes with the highest positive and lowest negative rank metric scores from representative GSEA pathways shown in (**D**). Data information: NES normalized enrichment score, FDR false discovery rate. *n* = 4 fish per genotype.

We next asked how loss of cGAS affects killifish physiology in vivo by performing bulk RNAseq on kidneys from young (7–8 week) healthy WT and cGAS KO fish (Fig. 2A). We focused on the kidney because of its central role in hematopoiesis, immune cell production, teleost primary lymphoid function (Bjorgen and Koppang, 2021), as well as its implication in inflammaging (Morabito et al, 2026). In addition, cGAS is highly expressed in this tissue (Costa et al, 2026) (Fig. EV2A).

Principal component analysis (PCA) and minus-average (MA) plots showed minimal transcriptomic differences between young WT and cGAS KO kidneys (Fig. 2B,C). To detect coordinated pathway-level changes, we used gene set enrichment analysis (GSEA) and identified several significantly enriched KEGG pathways. Notably, immune-related pathways, including Rig-I-like receptor, Toll-like receptor and Nod-like receptor signaling, as well as complement and coagulation cascades, were upregulated in the cGAS KO relative to wild-type (Fig. 2D). Closer inspection of these genes revealed that many act downstream of or modulate cGAS signaling (Decout et al, 2021), namely *Sting1*, *Tbk1*, *Nfkb1*, *Rela*, *Ikbkg*, *Tnfa*, as well as *Traf6*, *Il12bb*, and *Ripk1l*, consistent with compensation for cGAS loss (Fig. 2E). Other gene categories upregulated in cGAS KO included lysosome function (*Ctsz*, *Ctsb*, *Atp6voc*, and *Atp6vob*), consistent with enhanced recycling capacity, and apoptosis (*Casp7*, *Map3k14*, *Bax*, and *Birc2*), suggesting increased potential for cell death signaling. Although these expression changes were small and individual genes did not remain significant after Benjamini–Hochberg correction (DESeq2 analysis), they collectively contributed to significant pathway enrichment in GSEA. Together, these changes suggest mild compensatory activation of pathways downstream of cGAS in cGAS KO kidneys.

We also performed RNAseq on gut samples from young WT and cGAS KO killifish. We selected the gut since it serves as the first line of defense against pathogens. As in the kidney, we saw neither clear separation of genotypes in the PCA plot, nor a substantial impact on individual gene regulation (Fig. EV2B,C). GSEA revealed that pathways related to the complement system and ribosome were significantly upregulated, while pathways associated with DNA replication, cell cycle, and aminoacyl tRNA biosynthesis were downregulated in cGAS KO guts relative to WT (Fig. EV2D). However, gene expression across biological replicates was highly variable (Fig. EV2E) and thus we conclude that cGAS KO has limited effects on the gut transcriptome.

### Killifish cGAS modulates the transcriptional response to DNA damage and senescence in vivo

**Figure 3:**
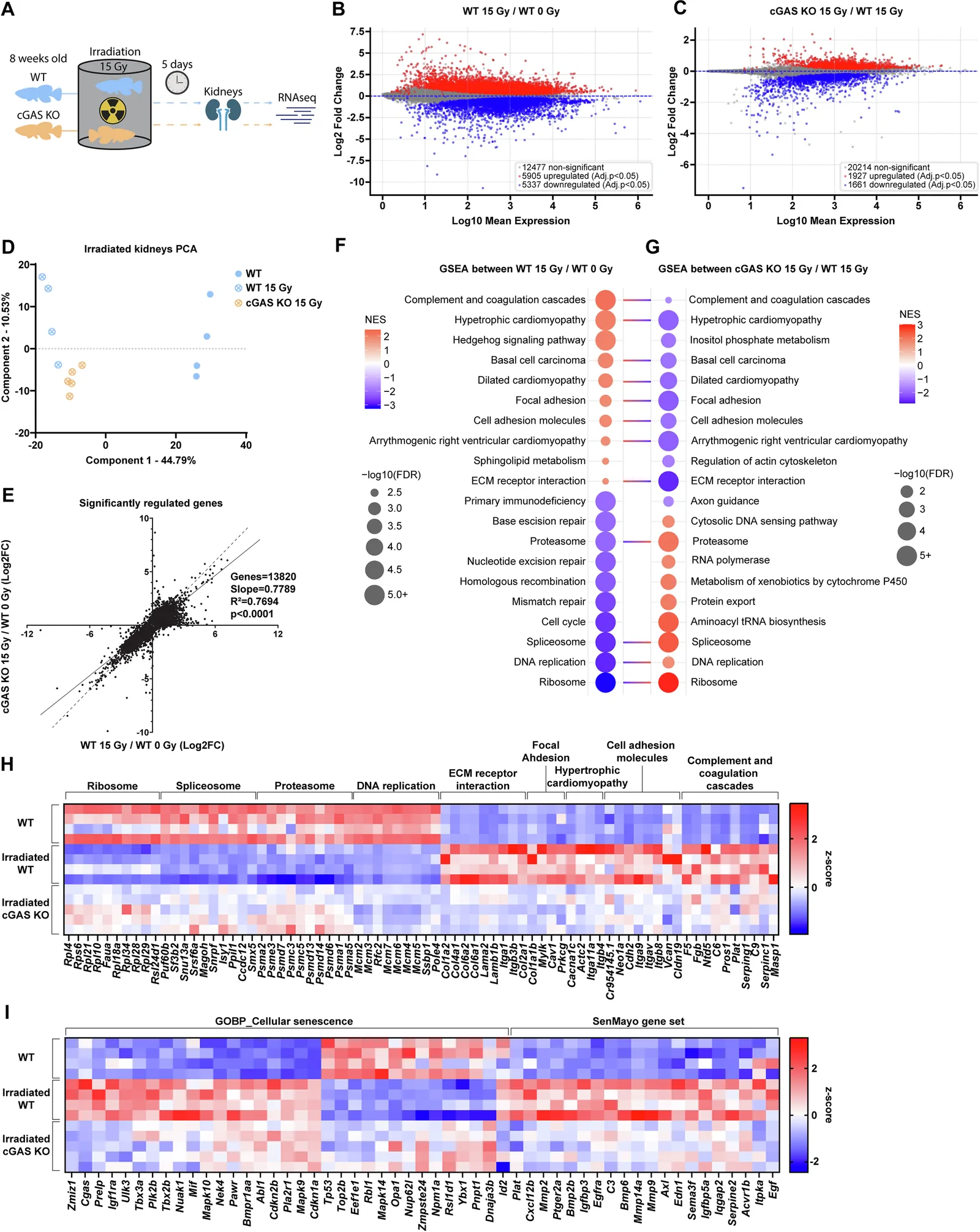
cGAS modulates DNA damage-induced transcriptional changes in vivo. (**A**) Schematic overview of samples used for transcriptomic profiling. RNAseq was performed on kidneys from young 8-week-old WT and cGAS KO animals, 5 days after exposure to 15 Gy γ-radiation. (**B**) MA plot showing differentially expressed genes from irradiated versus non-irradiated young WT kidneys. Genes having an adjusted *P* value < 0.05 were considered significantly upregulated (red) or downregulated (blue). (**C**) MA plot showing differential mRNA expression comparing irradiated cGAS KO versus irradiated WT kidneys. (**D**) Principal Component Analysis of transcriptomes from non-irradiated and irradiated WT and irradiated age-matched cGAS KO kidneys. (**E**) Linear regression analysis of the log2-Fold Change (Log2FC) in significantly expressed genes regulated by irradiation. The *x* axis represents the Log2FC between irradiated and non-irradiated WT kidneys, and the *y* axis represents the Log2FC between irradiated cGAS KO and non-irradiated WT kidneys. Genes significantly regulated in both comparisons are shown. The solid line indicates the linear regression fit, while the dashed line represents the reference slope = 1 (*x* = *y*). *P* value indicates comparison between the observed slope and slope = 1, and the value is ~3.3*10^−808^. (**F**) GSEA comparing irradiated to non-irradiated WT kidneys. The top KEGG pathways upregulated and downregulated in irradiated WT, ranked by normalized enrichment score. (**G**) GSEA comparing irradiated cGAS KO and irradiated WT kidneys. KEGG pathways commonly regulated by irradiation and cGAS loss are positioned adjacent to their counterparts in (**F**), while remaining pathways are ranked independently. (**H**) Heatmap of z-score-normalized expression values of genes with the highest positive and lowest negative rank metric scores from representative GSEA pathways shown in (**F**, **G**). (**I**) Heatmap of z-score-normalized expression values for genes with the highest positive and lowest negative rank metric scores from GSEA comparing irradiated to non-irradiated WT kidneys, using the SenMayo and GOBP Cellular Senescence gene sets. Data information: GOBP Gene Ontology Biological Process. *N* > = 4 fish per genotype and treatment. Some elements of the illustration in (**A**) were created with biorender.com. Source data are available online for this figure.

We next examined the response of cGAS-deficient killifish to irradiation in vivo. Young WT and cGAS KO fish were exposed to 15 Gy of γ-radiation, and the kidney transcriptomes were examined 5 days later (Fig. 3A) to capture early senescence-associated responses (Schoetz et al, 2021; Turnquist et al, 2019). Given the minimal basal differences between genotypes (Fig. 2C), young WT fish served as the reference for subsequent analyses. This approach avoided a four-way interaction analysis, which would require considerably larger sample sizes because of inter-individual variability (Duda et al, 2023). As expected, irradiation triggered widespread transcriptional changes in WT fish (Fig. 3B). In this case, PCA clearly separated non-irradiated WT, irradiated WT, and irradiated cGAS KO samples (Fig. 3D), and the irradiated cGAS KO transcriptome visibly diverged from irradiated WT (Fig. 3C), indicating that cGAS modulates the transcriptional response to DNA damage.

Linear regression of all genes significantly regulated by irradiation in WT versus cGAS KO kidneys revealed a strong correlation (*R*^2^ = 0.7694), indicating that WT and cGAS-deficient fish activate largely similar transcriptional programs relative to WT unirradiated controls (Fig. 3E). However, the regression slope was significantly less than 1 (*m* = 0.7789), consistent with an attenuated transcriptional response in cGAS KO kidneys (Fig. 3E), in agreement with our in vitro findings. Few genes were oppositely regulated in irradiated cGAS KO, indicating that cGAS modulates the magnitude rather than the direction of transcriptional responses. Consistent with the linear regression analysis, GSEA revealed that most pathways showed significantly dampened regulation in the irradiated cGAS KO kidneys (Fig. 3F,G). Several pathways induced upon irradiation in WT were induced less strongly in irradiated cGAS KO, including innate immunity (complement components *C6*, *C9*, *Serping1*), extracellular matrix organization, and cell–cell interaction (*Col1a2*, *Lama2*, *Cdh*, *Itga9*, *Cav1*, *Mylk*, *Itgb4*) (Fig. 3G,H), consistent with attenuation of senescence-associated programs (Wolfson et al, 2009). Conversely, several pathways repressed by irradiation in WT were less strongly repressed in irradiated cGAS KO, including ribosome (*Rpl4/10/21*, *Rps6*), spliceosome (*Puf60b*, *Sf3b2*, *Magoh*), proteasome (*Psma2*, *Psme3*, *Psmd7*), and DNA replication (*Mcm2/3/4/5/6/7*, *Rfc2*) (Fig. 3H)—processes mainly associated with growth and proliferation. Curiously, DNA repair pathways were downregulated in both cGAS KO and WT at this 5-day timepoint, consistent with previous observations that cells on a senescent trajectory downregulate DNA repair (Collin et al, 2018). Because KEGG pathways incompletely capture senescence and SASP factors, we specifically examined genes within the Cellular Senescence GO Biological Process (GOBP) term and the SenMayo gene set (Saul et al, 2022). Notably, we observed a blunted senescent-associated gene expression signature in the cGAS KO kidneys (Fig. 3I), particularly among SASP components (*Mif*, *Cxcl12b*, *Igfbp3/5a*, *Mmp2/9*), corroborating our in vitro findings.

Thus, DNA damage and senescence responses remain intact in kcGAS KO, but the magnitude is markedly reduced, suggesting that cGAS amplifies the transcriptional response of these processes in vivo.

**Figure EV3:**
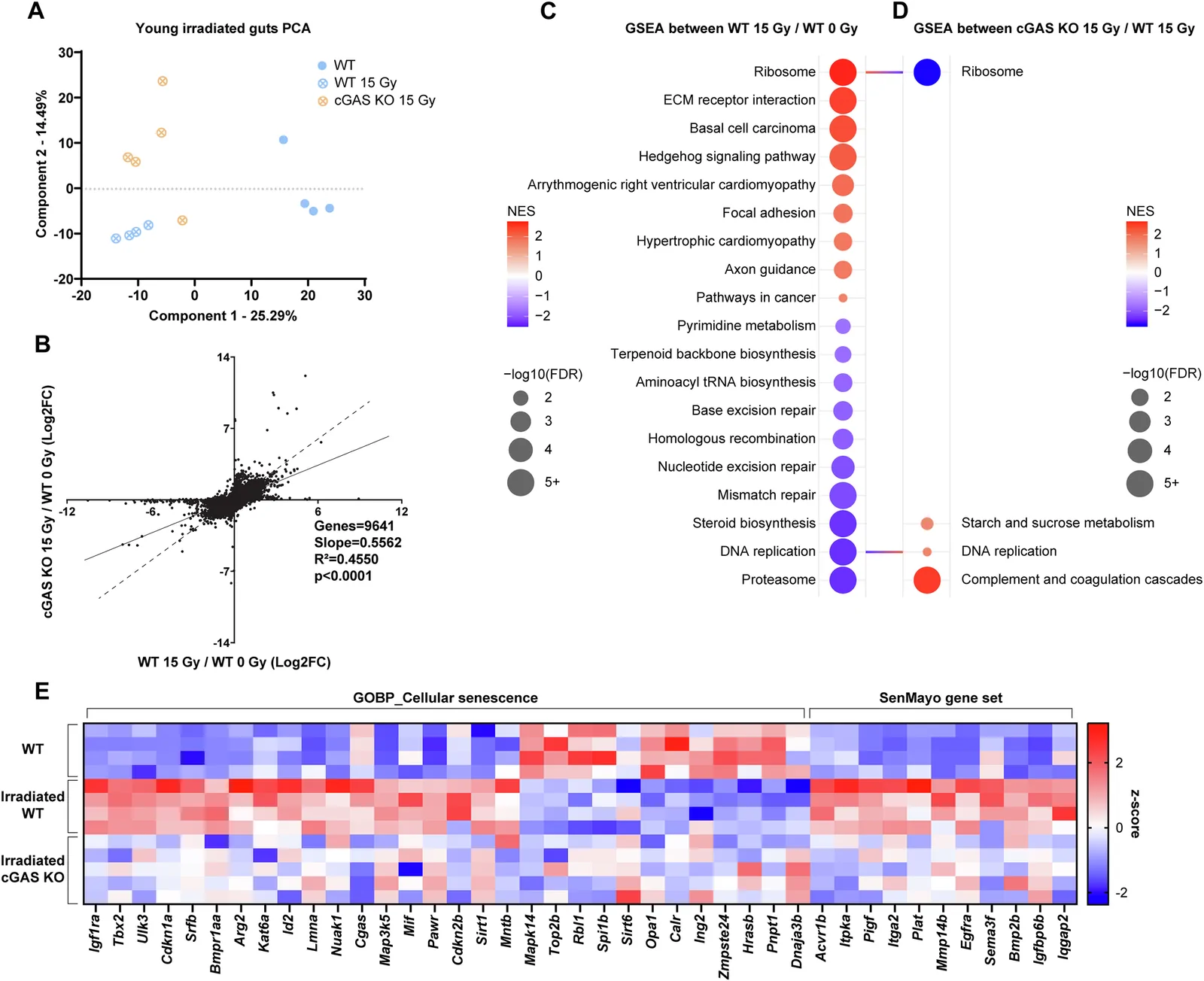
cGAS modulates DNA damage-induced transcriptional changes in killifish guts. (**A**) Principal component analysis of transcriptomes from young 8-week-old non-irradiated WT, irradiated (15 Gy) age-matched WT, and cGAS KO killifish guts. (**B**) Linear regression analysis of the Log2 Fold Change (Log2FC) of genes significantly regulated by irradiation. The *x* axis represents the Log2FC between irradiated and non-irradiated WT guts, and the *y* axis represents the Log2FC between irradiated cGAS KO and non-irradiated WT guts. Genes significantly regulated in both comparisons are shown. The solid line indicates the linear regression fit, while the dashed line represents the reference slope = 1 (*x* = *y*). *P* value indicates comparison between the observed slope and slope = 1, and the value is ~3.21*10^−1114^. (**C**) GSEA comparing irradiated to non-irradiated WT guts. The top KEGG pathways upregulated and downregulated in irradiated WT, ranked by normalized enrichment score. (**D**) GSEA comparing irradiated cGAS KO and irradiated WT guts. KEGG pathways commonly regulated by irradiation and cGAS loss are positioned adjacent to their counterparts in (**C**), while remaining pathways are ranked independently. (**E**) Heatmap showing z-score-normalized expression values for genes with the highest positive and lowest negative rank metric scores from GSEA comparing irradiated to non-irradiated WT guts, using the SenMayo and GOBP Cellular Senescence gene sets.

We also analyzed the gut transcriptome from the same irradiated fish. As with the kidney, PCA plots showed that genotypes clustered separately after irradiation (Fig. EV3a), and linear regression again showed a decreased slope (*m* = 0.5562), albeit with weaker correlation (*R*^2^ = 0.4550) (Fig. EV3b). GSEA revealed similar regulation of multiple pathways after irradiation (Fig. EV3C), which was blunted by cGAS deficiency (Fig. EV3C,D). Though attenuation of gene and KEGG pathway changes was less prominent than in the kidney, senescence and SASP activation were dampened to a similar extent (Fig. EV3E), suggesting that cGAS functions in multiple tissues during genotoxic stress, but the breadth of regulation appears tissue-specific.

### Killifish cGAS modulates the aging transcriptome

**Figure EV4:**
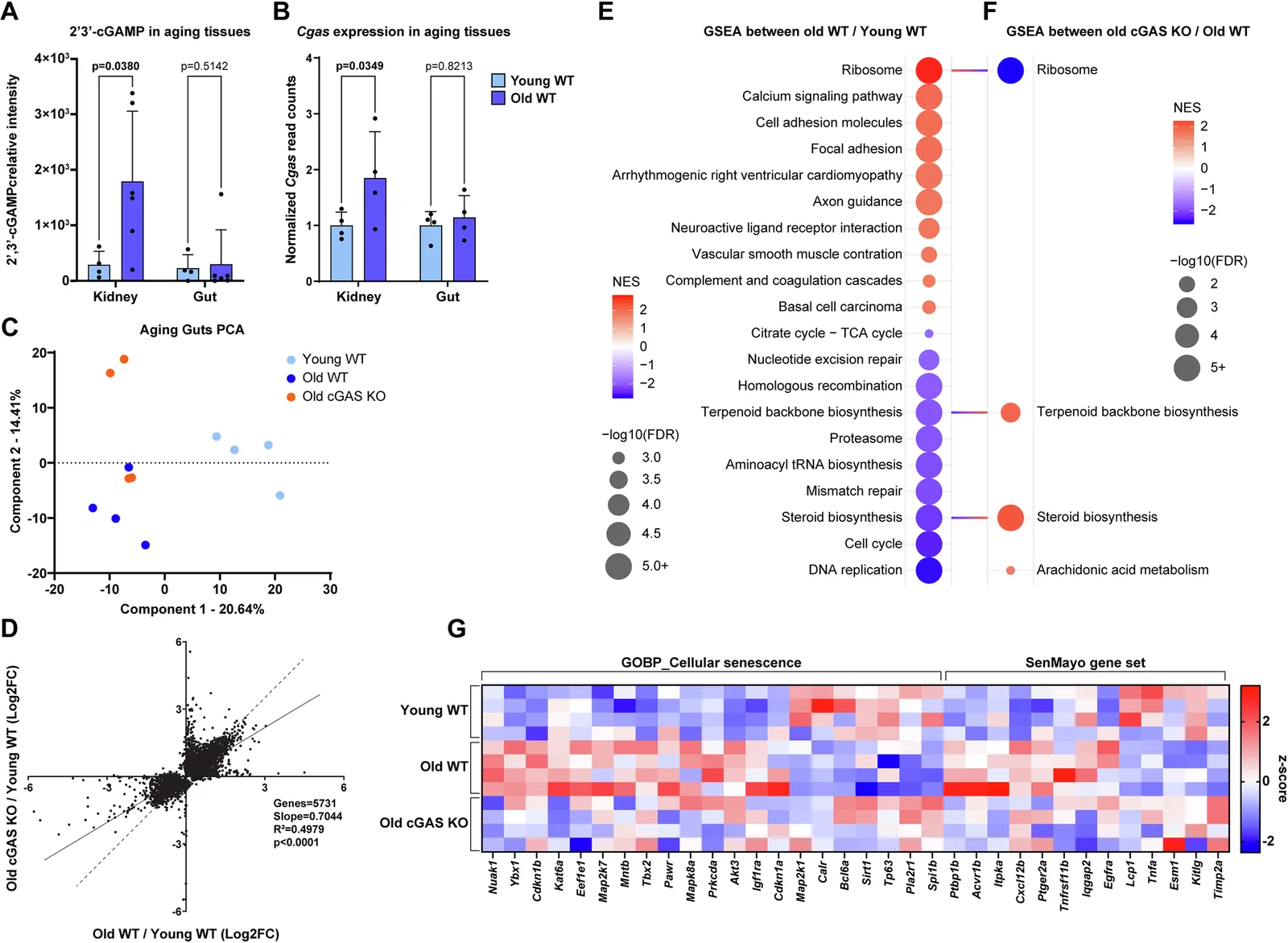
cGAS modulates senescent transcriptional signatures during aging in killifish guts. (**A**) LC–MS-based targeted metabolomics of 2’,3’-cGAMP extracted from young (7 weeks, *n* = 4) and old (18 weeks, *n* = 6) WT kidneys and guts. Statistical analysis was performed using the Mann–Whitney *U* test. Error bars indicate standard deviation from the mean. (**B**) Read counts of *Cgas* from RNAseq analysis in young and old kidneys and guts. Reads were normalized to the average of the young samples in each tissue. *P* values indicate Benjamini–Hochberg–adjusted *P* values obtained from the DESeq2 Wald test. Error bars indicate standard deviation from the mean. (**C**) PCA of gut transcriptomes from young (8 weeks) WT and old (18 weeks) WT and cGAS KO killifish. *n* = 4 for each condition. (**D**) Linear regression analysis of the Log2 Fold Change (Log2FC) of genes significantly regulated by aging. The *x* axis shows the Log2FC between old WT and young WT. The *y* axis shows the Log2FC between old cGAS KO and young WT. Genes significantly regulated in both comparisons are shown. The solid line indicates the linear regression fit, while the dashed line represents the reference slope = 1 (*x* = *y*). *P* value indicates comparison between the observed slope and slope = 1, and the value is ~1.67*10^−219^. (**E**) GSEA comparing old to young WT guts. Top KEGG pathways upregulated and downregulated in irradiated WT, ranked by normalized enrichment score. (**F**) GSEA comparing old cGAS KO and old WT guts. KEGG pathways commonly regulated by aging and cGAS are positioned adjacent to their counterparts in (**E**), while remaining pathways are ranked independently. (**G**) Heatmap showing z-score-normalized expression values of genes with the highest positive and lowest negative rank metric scores from GSEA comparing old to young WT guts, using the Sen_Mayo and GOBP_Cellular Senescence gene sets.

**Figure 4:**
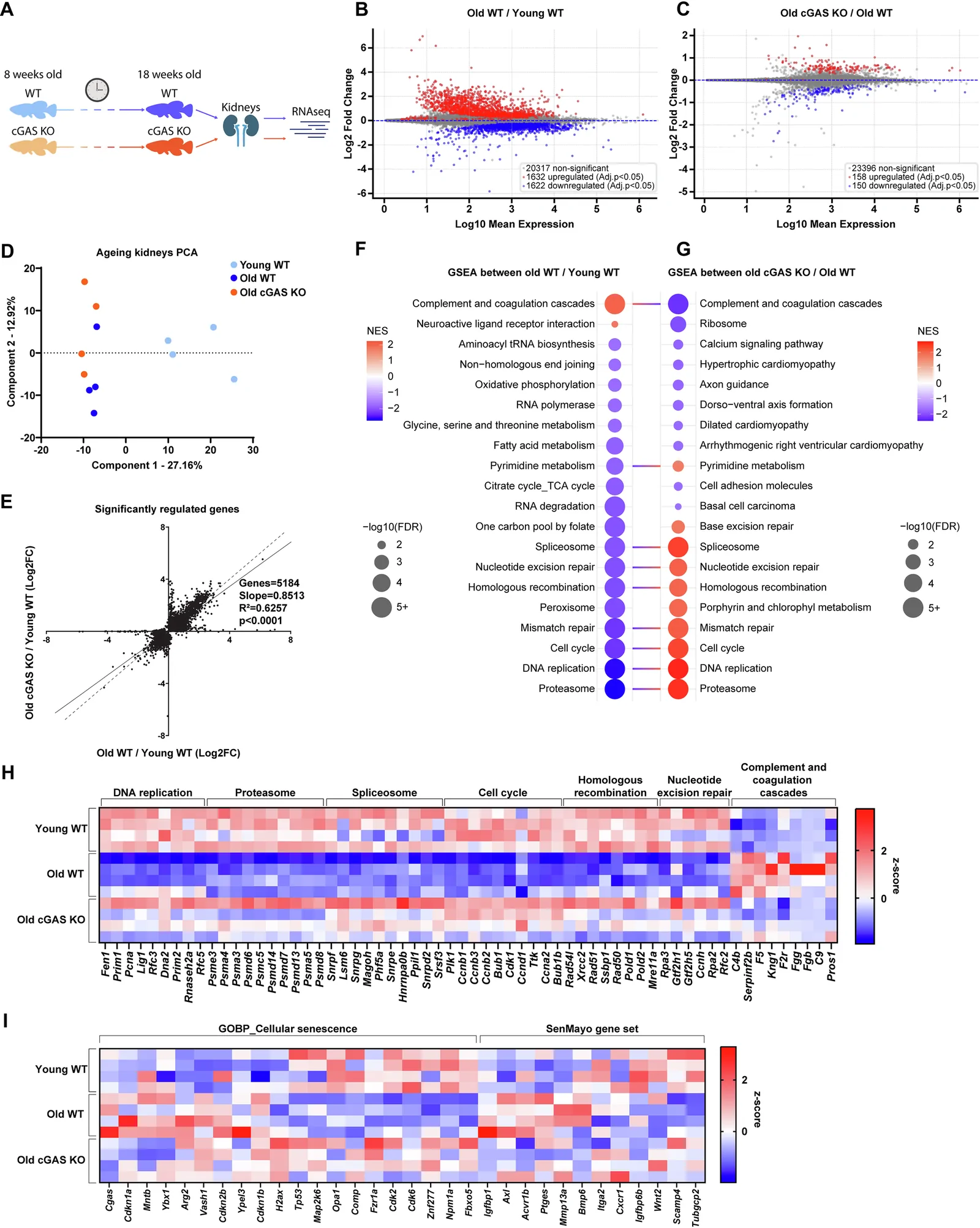
cGAS modulates transcriptional changes during aging. (**A**) Schematic overview of samples used for age-related transcriptomic analysis. RNAseq was performed on RNA from the kidneys of 18-week-old WT and cGAS KO fish. (**B**) MA plot showing differential mRNA expression in kidneys from old versus young WT animals. Genes with an adjusted *P* value < 0.05 were considered significantly upregulated (red) or downregulated (blue). (**C**) MA plot showing differential mRNA expression comparing old cGAS KO to old WT kidneys. (**D**) PCA plot of transcriptomes from young and old WT and old cGAS KO kidneys. (**E**) Linear regression analysis of the Log2 Fold Change (Log2FC) of genes significantly regulated during aging. The *x* axis represents the Log2FC of old WT and young WT kidneys, and the *y* axis represents the Log2FC of old cGAS KO and young WT kidneys. Genes significantly regulated in both comparisons are shown. The solid line indicates the linear regression fit, while the dashed line represents the reference slope = 1 (*x* = *y*). *P* value indicates comparison between the observed slope and slope = 1, and the value is ~5.49*10^−58^. (**F**) GSEA comparing old to young WT kidneys. The top KEGG pathways upregulated and downregulated in old WT are shown, ranked by normalized enrichment score. (**G**) GSEA comparing old cGAS KO and old WT kidneys. KEGG pathways commonly regulated by aging and cGAS loss are positioned next to their counterparts in (**F**), while remaining pathways are ranked independently. (**H**) Heatmap showing z-score-normalized expression values of genes with the highest positive and lowest negative rank metric scores from representative GSEA pathways shown in (**F**, **G**). (**I**) Heatmap showing z-score-normalized expression values of genes with the highest positive and lowest negative rank metric scores from GSEA comparing old and young WT kidneys, using SenMayo and GOBP Cellular Senescence gene sets. *N* = 4 for all conditions illustrated. Data information: Some elements of the illustration in (**A**) were created with biorender.com. Source data are available online for this figure.

We then examined the impact of cGAS activity during aging. We first asked whether cGAMP levels in killifish change with age, as reported in mice (Gulen et al, 2023), and found that cGAMP increased with age in the kidney, but not in the gut (Fig. EV4A), indicating tissue-specific regulation. We next analyzed the aging transcriptome, comparing young (8 weeks) healthy WT fish as a reference to old (18 weeks) WT and cGAS KO kidneys (Fig. 4A). In line with the cGAMP measurements, *Cgas* transcripts increased with age in kidneys but not in guts (Fig. EV4B).

Overall, the kidney transcriptome became dysregulated with aging, and the aged cGAS KO transcriptional profile deviated noticeably from the aged WT counterpart (Fig. 4B–D). Linear regression analysis and GSEA comparing old cGAS KO and old WT to young WT indicated age-related changes in gene expression that were blunted by cGAS KO (Fig. 4E–G), analogous to the response to irradiation. In particular, GSEA revealed that in the old cGAS KO kidneys, DNA replication and repair pathways, cell cycle, spliceosome, proteasome, and pyrimidine metabolism were less strongly repressed, while complement and coagulation cascades were less strongly induced. Notably, complement and coagulation cascades are a shared signature of inflammaging in both killifish (Costa et al, 2026; Morabito et al, 2026) and human (Costa et al, 2026; Li et al, 2026) tissues. In addition, aging cGAS KO kidneys showed less reduction in cell proliferation markers (*Fen1*, *Pcna*, *Rfc3*, *Ccnb1/2/3*) (Fig. 4H), suggesting enhanced proliferative capacity. Surprisingly, evidence was lacking for a strong senescence-associated transcriptional signature in the aged kidney (Fig. 4I) except for a handful of genes (*Cdkn1a*, *Tp53*, *Igfbp1*) that appeared less regulated, likely reflecting the scarcity of senescent cells within tissues during aging (Idda et al, 2020). Since killifish kidneys contain both highly proliferative cells (kidney marrow) and post-mitotic cells (renal cells), senescent signatures might be obscured in bulk transcriptomics.

In contrast to the kidney, the aging killifish gut transcriptome showed much less dependence on cGAS (Fig. EV4C,D), consistent with the lack of increased cGAMP and *Cgas* levels with age in this tissue (Fig. EV4A,B). Transcriptional dampening was less apparent than in the kidney (Fig. EV4E,F), though the induction of several senescent genes (*Nuak1*, *Cdkn1b/2b*, *Ptbp1*) was attenuated in cGAS KO guts (Fig. EV4G).

In sum, cGAS modulates the transcriptional landscape during both DNA damage and aging, but the extent and nature of the changes are tissue-specific.

### cGAS affects age-related pathology but not lifespan in killifish

**Figure 5:**
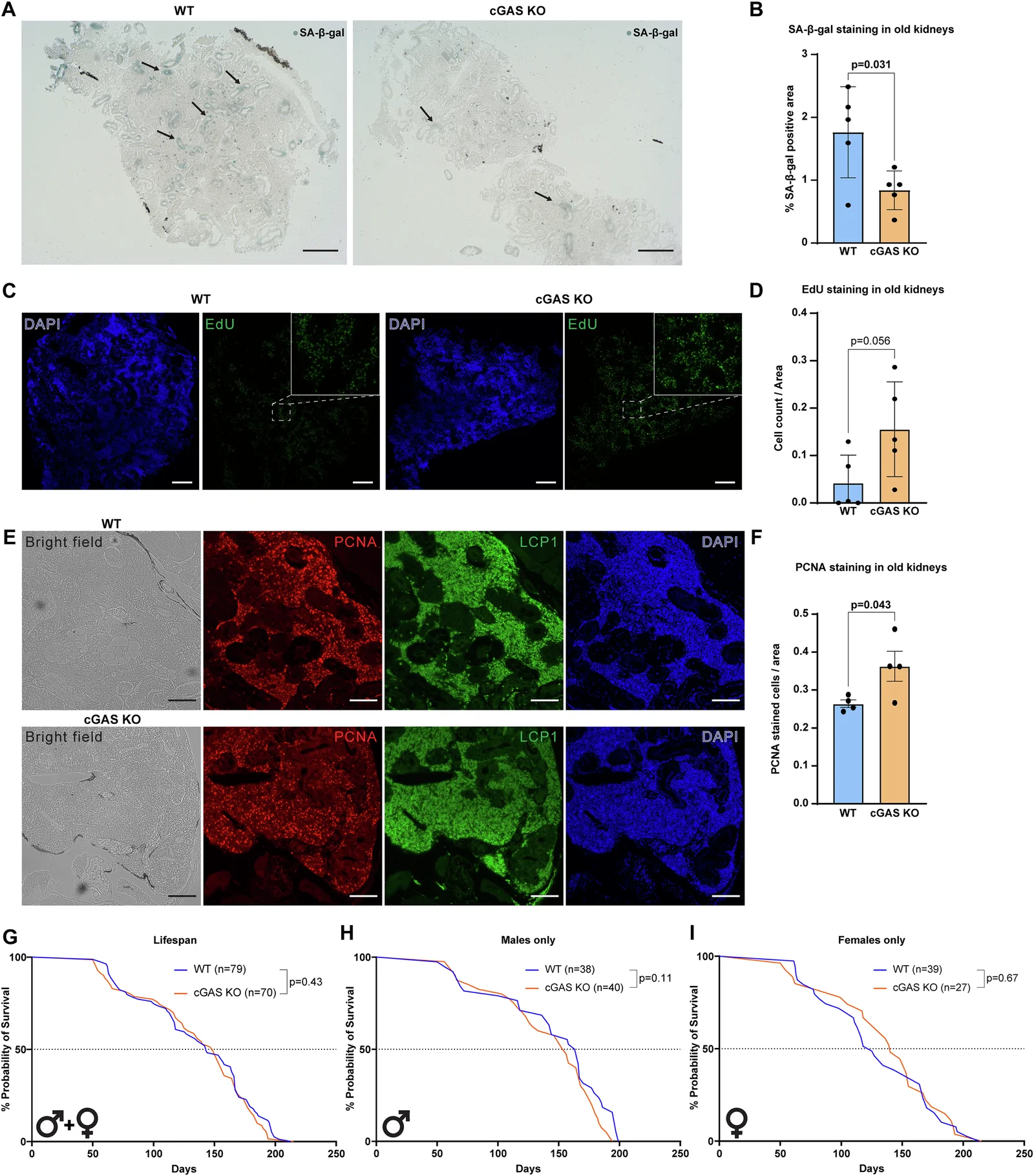
Lack of cGAS leads to reduced senescence burden and increased proliferation, but does not impact lifespan. (**A**) Representative true-color bright-field images of old (19 weeks) WT and cGAS KO kidneys stained for SA-β-galactosidase activity. Arrows indicate senescent renal tubules. Scale bars = 200 μm. (**B**) Quantification of β-gal positive area normalized to total kidney area. Each dot represents one fish, *n* = 5 per genotype. Statistical significance was determined using the Student’s *t* test. Error bars indicate standard deviation from the mean. (**C**) Representative images of old (19 weeks) WT and cGAS KO kidneys stained for DAPI and EdU incorporation. EdU was injected intraperitoneally, and tissues were harvested 6 h later. Scale bars = 150 μm. (**D**) Quantification of EdU-positive cells normalized to kidney area. Each dot represents one fish, *n *= 5 per genotype. Statistical significance was determined using the Mann–Whitney test. Error bars indicate standard deviation from the mean. (**E**) Representative images of old (18 weeks) WT and cGAS KO kidneys stained for LCP1, PCNA, and DAPI. Scale bars = 100 μm. (**F**) Quantification of PCNA-positive cells normalized to the kidney area. Each dot represents one fish, *n* = 4 per genotype. Statistical significance was determined using the Student’s *t* test. Error bars indicate standard deviation from the mean. (**G**) Kaplan–Meier survival curves comparing WT (*n* = 79) and cGAS KO (*n* = 70) animals. *P* value indicates the comparison between genotypes using the log-rank Mantel–Cox test. (**H**, **I**) Kaplan–Meier curve showing the survival of male and female WT and cGAS KO fish, respectively. *P* value indicates the comparison between genotypes using the log-rank Mantel–Cox test. Fish (*n*) numbers per sex and genotype are shown in brackets. Data information: At least two sections were stained per fish in (**A**–**F**). Source data are available online for this figure.

Because killifish cGAS KO mitigated senescence in cultured fibroblasts and attenuated age-related transcriptional signatures in the kidney in vivo, we sought to investigate if these changes were reflected in tissue physiology and pathology. We first investigated SA-β-gal activity in renal sections of old (19 weeks) WT and cGAS KO kidneys (Fig. 5A). Histological assessment showed a significant decrease in senescent cells in old cGAS KO kidneys compared to age-matched WT (Fig. 5A,B). Staining was seen mostly in the renal tubules rather than in the proliferative marrow.

Transcriptomic analysis also suggested increased cell proliferation in cGAS KO kidneys. Accordingly, cGAS KO kidneys consistently showed greater EdU incorporation, with higher staining intensity and cell numbers compared to WT (Fig. 5C,D). An independent cohort of old fish (18 weeks) stained for Proliferating Cell Nuclear Antigen (PCNA) (Fig. 5E), confirmed that kidneys from old cGAS KO enhanced cell proliferation compared to old WT (Fig. 5F). Together, these data indicate that cGAS loss results in decreased senescence and increased cell proliferation in the old kidney. We next sought to measure inflammation by co-staining kidneys with LCP1 (L-Plastin), a histological marker of immune infiltration (Fig. 5E). However, since LCP1 was widespread in the marrow, we could not distinguish infiltration from natural immune cell production.

**Figure EV5:**
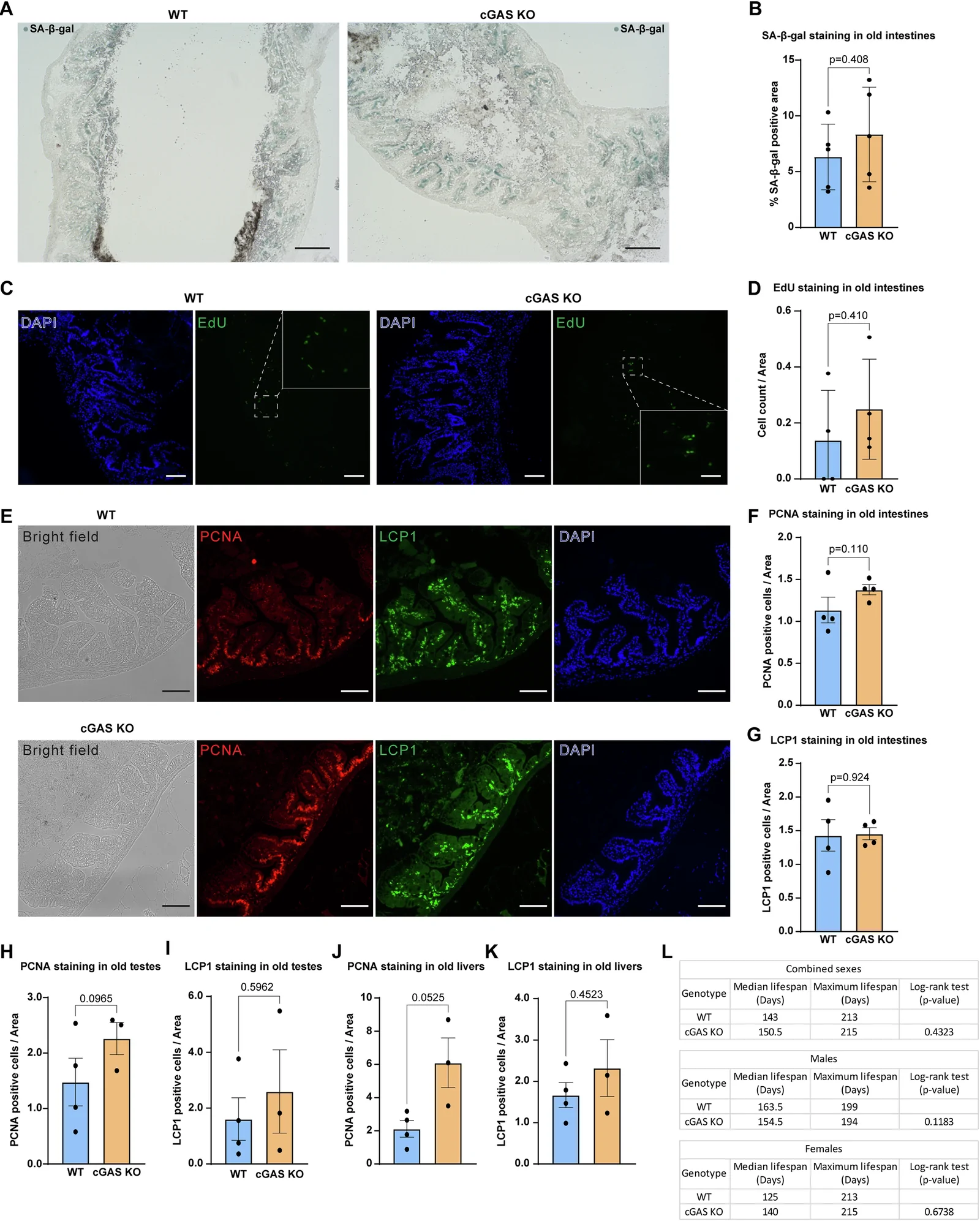
Lack of cGAS does not significantly affect intestinal proliferation or immune infiltration in old age. (**A**) Representative true-color bright-field images of old (19 weeks) WT and cGAS KO intestines stained for senescence-associated β-galactosidase activity. Scale bars = 200 μm. (**B**) Quantification of β-gal positive area normalized to intestinal area. Each dot represents one fish. *n* = 5 per genotype. Statistical significance was determined using the Student’s *t* test. Error bars indicate standard deviation from the mean. (**C**) Representative images of old (19 weeks) WT and cGAS KO intestines stained for DAPI and EdU incorporation. EdU was injected intraperitoneally, and tissues were harvested 6 h later. Scale bars = 150  μm. (**D**) Quantification of EdU-positive cells normalized to intestinal area. Each dot represents one fish. *n* = 5 per genotype. Statistical significance was determined using the Mann–Whitney test. Error bars indicate standard deviation from the mean. (**E**) Representative Images of old (18 weeks) WT and cGAS KO intestines stained for LCP1, PCNA, and DAPI. Scale bars = 100 μm. (**F**) Quantification of PCNA-positive cells normalized to intestinal area. Each dot represents one fish. *n* = 4 per genotype. Error bars indicate standard deviation from the mean. (**G**) Quantification of LCP1-positive cells normalized to intestinal area. Each dot represents one fish. *n* = 4 per genotype. Error bars indicate standard deviation from the mean. (**H**) Quantification of PCNA-positive cells normalized to testis area. Each dot represents one fish. *n* = 4 for WT, *n* = 3 for cGAS KO. Error bars indicate standard deviation from the mean. (**I**) Quantification of LCP1-positive cells normalized to testis area. Each dot represents one fish. *n* = 4 for WT, *n* = 3 for cGAS KO. Error bars indicate standard deviation from the mean. (**J**) Quantification of PCNA-positive cells normalized to liver area. Each dot represents one fish. *n* = 4 for WT, *n* = 3 for cGAS KO. Error bars indicate standard deviation from the mean. (**K**) Quantification of LCP1-positive cells normalized to liver area. Each dot represents one fish. *n* = 4 for WT, *n* = 3 for cGAS KO. Error bars indicate standard deviation from the mean. (**L**) Tables summarizing median and maximum lifespans (in days), and *P* values calculated from Mantel–Cox log-rank statistics. Genotype and sex are indicated. Data information: At least two sections were stained per fish in (**A**–**K**). Statistical significance in (**F**, **G**–**K**) was determined using Student’s *t* test.

We also investigated physiological parameters in the intestine. Despite dampened transcriptional signatures of senescence, intestinal SA β-gal activity did not differ significantly between genotypes (Fig. EV5A,B), since the intestine had uniformly high β-gal activity. EdU incorporation and PCNA staining also showed no genotype-dependent differences (Fig. EV5C–F). Although intestinal LCP1 staining could clearly capture immune cell infiltration (Fig. EV5E), there was no apparent difference between genotypes (Fig. EV5G). Likewise, analysis of LCP1 and PCNA staining in male gonads and liver did not reveal statistically significant differences (Fig. EV5H–K). While PCNA staining showed modest increases across tissues in the cGAS KO, these differences did not reach statistical significance.

In light of the dampened transcriptional signatures of aging, reduced senescence burden, and increased proliferation in the kidneys, we hypothesized that cGAS mutants might prolong life. To test this, we measured the longevity of cGAS KO (*n* = 70) and WT controls (*n* = 79) after ≥4 rounds of backcrossing, using singly housed males and females from the same cohort. Surprisingly, cGAS KO fish had overall similar lifespans to WT (Fig. 5G–I), contravening our hypothesis. Median (143 days for WT and 150.5 days for cGAS KO) and maximum lifespan (213 days for WT and 215 days for cGAS KO) were comparable between genotypes. Survival curves were not significantly different either in single sexes or combined (Fig. EV5L). Thus, despite attenuating molecular and cellular hallmarks of aging, cGAS loss is not limiting for killifish lifespan.

**Figure EV6:**
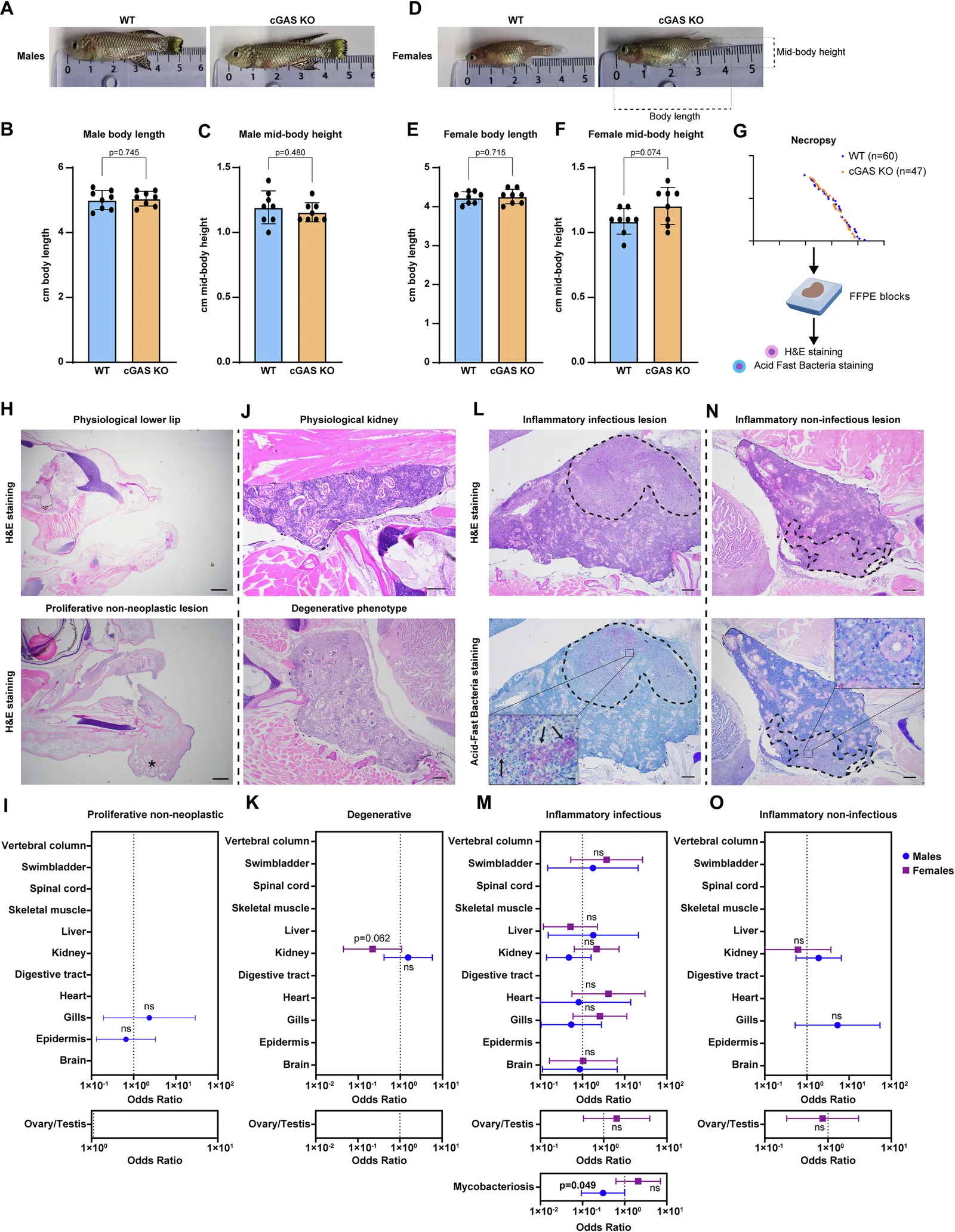
Killifish cGAS contributes to age-related pathology. (**A**) Representative images showing gross male body size of old (19 weeks) WT and cGAS KO killifish. (**B**) Quantification of male body length and (**C**) mid-body height. Each dot represents one fish. Error bars indicate standard deviation from the mean. (**D**) Female gross body size in old (19 weeks) WT and cGAS KO killifish. (**E**) Quantification of female body length and (**F**) mid-body height. Each dot represents one fish. Error bars indicate standard deviation from the mean. (**G**) Schematic overview of fish retrieved for necropsy analysis from lifespan experiments (Fig. 5G–I), showing FFPE sections stained for hematoxylin & eosin (H&E) and acid-fast bacteria. (**H**) Representative H&E histological images of the jaw region depicting a normal lower lip and proliferative non-neoplastic lesion (*). (**I**) Forest plots for non-neoplastic proliferative lesions across 12 tissues in WT and cGAS KO fish. Center markers denote the point estimate of the odds ratio (OR), with error bars representing 95% confidence intervals (CI). Results summarize a multivariate logistic regression model used for interaction analysis (Pathology ~ Genotype + Sex + Genotype:Sex + Age). The OR for the female subgroup, purple square; OR for the male subgroup, a blue circle. OR and CI bars are omitted where the event incidence was zero. Statistical significance (see “Methods”) is indicated adjacent to each plotted OR. (**J**) H&E histological images depicting a healthy kidney and a kidney displaying degenerative tubular changes. (**K**) Forest Plots illustrating the OR and 95% CI for degenerative pathology. Data were analyzed as in (**I**). (**L**) Representative H&E and acid-fast bacteria-stained histological images of a kidney depicting an inflammatory lesion infiltrated by mycobacteria. The outlined area contains necrosis and mononuclear inflammatory infiltrates, inset shows aggregates of acid-fast-positive rod-shaped bacteria, indicated by arrows. (**M**) Forest Plots illustrating the OR and 95% CI for inflammatory infectious lesions. Among the infectious lesions, mycobacteriosis incidence (positive for acid-fast staining) is plotted separately and indicates occurrence per fish. Data were analyzed as in (**I**). (**N**) Representative H&E and acid-fast bacteria-stained histological images of a kidney depicting an inflammatory lesion with no apparent bacterial infiltration. The inflammatory cell aggregate within the kidney is outlined. Neither positive nor negative staining acid-fast rod-shaped organisms were detected in these lesions. (**O**) Forest Plots illustrating the OR and 95% CI for inflammatory non-infectious lesions. Data were analyzed as in (**I**). Data information: For male and female size comparisons in (**A**–**F**), *n* = 8 fish were measured per sex and genotype. Statistical analysis was performed using Student’s *t* test. *n* = 60 WT fish and *n* = 47 cGAS KO fish were used for necropsy in (**G**–**O**). 32 WT were female, and 28 were male, while 17 cGAS KO were female and 30 were male. Scalebars in kidney images = 200 µm, insets =  10 µm. Scalebars in jaw images = 500 µm.

We then investigated additional aspects of physiology and pathology. Whereas previous studies reported that cGAS KO mice exhibit smaller body size and obesity at older ages (Martinez et al, 2026), kcGAS KO fish showed no obvious difference in body size (Fig. EV6A–F). A subset of KO females displayed slightly enlarged abdomens, possibly due to ovarian inflammation (Fig. EV6D,F).

To ascertain the cause of death, fish from the lifespan analysis were preserved in paraformaldehyde and subjected to histological necropsy by a certified veterinary pathologist. Twelve tissues were analyzed using 60 WT and 47 cGAS KO animals (Fig. EV6G). Lesions were characterized as neoplastic, proliferative non-neoplastic, degenerative, inflammatory infectious, and inflammatory non-infectious. No clear neoplastic lesions in any of the tissues were identified, suggesting that killifish have a lower cancer incidence under our laboratory conditions. This contrasts with mice and could explain species-specific differences in cGAS KO phenotypes.

Non-neoplastic lesions were detected predominantly at the mandibular lip, which was enlarged and contained nodular fibroplasia (Fig. EV6H), while a few fish showed proliferative bronchitis with or without goblet cell hyperplasia. The most common degenerative lesions were found in the kidney in the form of minimal to mild tubular dilation with or without luminal mineralization (Fig. EV6I). Infectious and non-infectious inflammatory lesions were widespread across tissues but most prominent in the kidneys and ovaries (Fig. EV6J,K). Infectious lesions often contained acid-fast positive rod-shaped bacteria, consistent with mycobacterial infection (Fig. EV6J). These infections usually affect multiple organs at once within an individual. Inflammatory non-infectious lesions were classified when significant immune cell infiltration was observed without detectable rod-shaped microorganisms (Fig. EV6K). These lesions were far less prevalent than infectious lesions overall.

To assess differences in pathology, we used a multivariate logistic regression model incorporating genotype, date of death, sex, and pathology category and plotted the odds ratio and 95% confidence intervals (CIs) (Fig. EV6I–O). Consistent with the minimal differences in lifespan, no genotype-dependent differences in age-related disease occurrence across tissues and sexes were apparent. Exceptionally, a significantly lower occurrence of mycobacteriosis in male cGAS KO fish than WT was seen (Fig. EV6N), reflecting a decrease in the incidence of individual infection. This result is surprising since cGAS functions in innate immunity, yet its reduction wards off bacterial infection in the laboratory setting. In addition, female kidneys tended to have fewer degenerative lesions with age (Fig. EV6M). However, it is important to emphasize that the differences in odds ratios were small, and the sample size was limited, restricting the ability to discern subtle effects.

In sum, we find that killifish cGAS has conserved and species-specific functions in senescence, cell proliferation, DNA damage, and aging. Killifish cGAS exhibits higher intrinsic enzymatic activity than human cGAS, and robustly expresses type I interferon signaling in response to cytosolic DNA. In line with mammalian counterparts (Dou et al, 2017; Yang et al, 2017), cGAS knockout mitigates DNA damage-induced cellular senescence in cultured killifish fibroblasts. In vivo, cGAS KO dampens the transcriptional response to DNA damage, inflammation, and senescence in young animals, and attenuates aging-related transcriptomic signatures in old animals. Accordingly, cGAS KO kidneys show lower SA-b-gal staining, and higher proliferative markers, mirroring mouse models (Gulen et al, 2023).

Despite the attenuation of age-related signatures and senescence, the cGAS KO does not extend lifespan. This unexpected result suggests that the benefits of reduced senescence are offset by other physiologic constraints. Multiple mechanisms may underlie this observation. For example, compensatory upregulation of the cGAS-STING regulatory cascade may foster low-level chronic inflammation despite cGAS loss (Salminen et al, 2008). Nevertheless, immune infiltration remained unchanged with age, and cGAS KO males actually showed reduced mycobacteriosis incidence, implying that compensatory immune pathways may protect against infection under laboratory conditions (Li et al, 2013). In parallel, enhanced cellular proliferation alongside increased intrinsic damage could impose opposing forces to tissue maintenance.

Interestingly, while human and mouse cGAS can inhibit DNA repair (Jiang et al, 2019; Liu et al, 2018), naked mole-rat cGAS promotes repair (Chen et al, 2025), paralleling kcGAS function and highlighting species-specific adaptation. Notably, killifish cGAS harbors one of four amino acid substitutions (E511) that are genoprotective in naked-mole rat cGAS (Chen et al, 2025), though additional residues may contribute. These differences underscore that cGAS is under strong evolutionary selection, balancing genome integrity, immune surveillance, and senescence (Hancks et al, 2015; Martinez et al, 2026; Zhen et al, 2023).

Our findings also reveal tissue-specific effects of cGAS inhibition. Amelioration of age-related signatures and SA-β−gal was more pronounced in the kidney, while attenuation of senescent gene expression was more prominent in the aged intestine. Inhibition of STING dampens late-life inflammation and neurodegeneration in the mouse brain (Gulen et al, 2023). Tissues vary in their degree of DNA damage (Wang et al, 2012a), senescent load (Yousefzadeh et al, 2020), and immune activation (Tabula Muris, 2020) with age, potentially engaging the cGAS/STING pathway to various extents. Furthermore, senescence and SASP can also have protective or regenerative roles (Coppe et al, 2010; Gorgoulis et al, 2019; Huang et al, 2022), hence chronic inhibition of cGAS might lead to mixed outcomes at the organismal level. Conceivably, partial or selective loss of function might have different physiological consequences.

Several limitations warrant consideration. The RNA-seq data were restricted to males and a few tissues. Though lifespan analysis included both sexes, the numbers may not be sufficient to discern subtle sex- or genotype-specific differences. Many tools and reagents needed to assess innate immune function remain underdeveloped in the killifish.

Collectively, this study establishes kcGAS as a conserved modulator of senescence, inflammation, and DNA damage responses, while revealing that attenuation of these cellular aging pathways does not necessarily translate into extended lifespan. Evidently, lifespan is governed by a complex interplay of factors beyond cellular senescence, including tissue-specific stress responses, compensatory immune signaling, and genomic maintenance.

## Methods

**Table:** Reagents and tools table

Reagent/resource	Reference or source	Identifier or catalog number
**Experimental models**		
Killifish (*N. furzeri*)	This study	GRZ-AD strain
Killifish primary fibroblasts (*N. furzeri*)	This study	N/A
THP1-Dual™ KO-cGAS Cells (*H. sapiens*)	Invivogen	#thpd-kocgas
**Recombinant DNA**		
pSBbi-GN-kcGAS	This study	N/A
pSBbi-GN-hcGAS	This study	N/A
pSBbi-GN-kcGAS KO	This study	N/A
pSBbi-GN-6xHis-kcGASa	This study	N/A
pSBbi-GN-6xHis-kcGASb	This study	N/A
**Antibodies**		
Phospho-Histone H2A.X (Ser139) (20E3) Rabbit mAb	Cell Signaling	9718
Donkey anti-Rabbit IgG (H + L) Highly Cross-Adsorbed Secondary Antibody, Alexa Fluor™ 488	ThermoFisher	A21206
Anti-6X His tag® antibody [HIS.H8]	Abcam	ab18184
Peroxidase AffiniPure® Donkey Anti-Mouse IgG (H + L)	Jackson ImmunoResearch	715-035-151
Lcp1 antibody	GeneTex	GTX124420
Anti-PCNA antibody [PC10] Mouse monoclonal (ab29)	Abcam	[PC10] (ab29)
Donkey anti-Mouse IgG (H + L) Highly Cross-Adsorbed Secondary Antibody, Alexa Fluor™ 568 - A10037	Invitrogen	A10037
**Primers**	**Sequence**	**Notes**
cGAS KO genotyping Forward	GTTAAGGAACCCCTTCGCACT	Used for genotyping cGAS mutant fish
cGAS KO genotyping Reverse	TTGCCGTCATCTCCCATTCTG	Used for genotyping cGAS mutant fish
Killifish Eif3c Forward	ggttaccagcagaagcagtc	Used for qPCR
Killifish Eif3c Reverse	tgttgaggaatgaagacgacg	Used for qPCR
Killifish GAPDH Forward	CGACGATATCAAGAAGGTTGTG	Used for qPCR
Killifish GAPDH Reverse	CAAAGATGGAGGAGTGGCAGTC	Used for qPCR
Killifish cGAS Forward	GTTTGGCGGGTTTCCTTTTCT	Used for qPCR
Killifish cGAS Reverse	TCTTGACGTGGTAGGAGCAG	Used for qPCR
Killifish p15 (CDKN2B) Forward	AGCTGCGGTGAACGGGGTG	Used for qPCR
Killifish p15 (CDKN2B) Reverse	GTCGTGCAACGGGGTGGTC	Used for qPCR
Killifish p21 (CDKN1A) Forward	TGCCCTACAGATCCAGCGTC	Used for qPCR
Killifish p21 (CDKN1A) Reverse	TTCCGTCTCTGATGTTGTCTC	Used for qPCR
Killifish IFI44 Forward	CCAGAGATGTGAGTGAGGTC	Used for qPCR
Killifish IFI44 Reverse	TGGAACGATGTAGGAGGTGG	Used for qPCR
Killifish ISG20 Forward	TTACAACACGTCTTCTGAGTCG	Used for qPCR
Killifish ISG20 Reverse	ACCTTTCCTCCCAGCCTGTATC	Used for qPCR
kcGAS Gi-As Forward	ctaccccaagctggcctctgCGCGTAGCAGTTTCAGCCAA	Used to clone kcGAS into pSBbi-GN using Gibson Assembly
kcGAS Gi-As Reverse	atccccaagcttggcctgacCCCCAGTTTCTACCTGGGATTCA	Used to clone kcGAS into pSBbi-GN using Gibson Assembly
Human GAPDH Forward	GAGTCAACGGATTTGGTCGT	Used for qPCR
Human GAPDH Reverse	GACAAGCTTCCCGTTCTCAG	Used for qPCR
Human IFNa-all Forward	AATCTCTCCTTTCTCCTG	Used for qPCR. Binds to a region common to all IFNa isoforms.
Human IFNa-all Reverse	TCTGACAACCTCCCAGGCACA	Used for qPCR. Binds to a region common to all IFNa isoforms.
Human IFNb Forward	CTTCTCCACTACAGCTCTTTCC	Used for qPCR. Binds to IFNb1 cDNA
Human IFNb Reverse	GCCAGGAGGTTCTCAACAAT	Used for qPCR. Binds to IFNb1 cDNA
Human IFI44 Forward	GTGAGCCTGTGAGGTCCAAG	Used for qPCR
Human IFI44 Reverse	CCTCCCTTAGATTCCCTATTTGCT	Used for qPCR
hcGAS Gi-As Forward	CTACCCCAAGCTGGCCTCTGCCGCCAGTAGTGCTTGGTTT	Used to clone hcGAS into pSBbi-GN using Gibson Assembly
hcGAS Gi-As Reverse	atccccaagcttggcctgacTGTATTCTCCAGGATTTAGGGTGAC	Used to clone hcGAS into pSBbi-GN using Gibson Assembly
**sgRNA for CRISPR/Cas9 in vivo**	**Sequence 5’ -** > **3’ (with PAM)**	
sgRNA targeting killifish cGAS	AAGGCTCCATTGTCGTCGAAAGG	
**Chemicals, enzymes, and other reagents**		
Dry pellet fish food	BioMar INICIO Plus G	518427
Alt-R S.p. HiFi Cas9 Nuclease	IDT	1081060
Dulbecco’s PBS	Gibco	14190144
PVP-I	Sigma-Aldrich	25655-41-8
Gentamicin	Gibco	15750060
Primocin®	InvivoGen	ant-pm-05
Collagenase P	Merck Millipore	12352204
Leibovitz’s L-15, 2 mM L-glutamine	Gibco	11415064
Trypsin-EDTA 0.05%	Gibco	25300054
RPMI 1640 4.5 g/L D-Glucose, 2.383 g/L HEPES, 2 mM L-glutamine, 1.5 g/L Sodium Bicarbonate, 110 mg/L Sodium Pyruvate	Gibco	11875093
2-Mercaptoethanol	Gibco	21985023
FBS	Gibco	10270106
Senescence β-Galactosidase Staining Kit	Cell Signaling	9860
Tissue-Tek® O.C.T. Compound	SAKURA	4583
Superfrost Plus slides	Epredia	H867.1
SfiI	NEB	R0123L
NEBuilder® HiFi DNA Assembly	NEB	E2621L
GeneXPlus	ATCC	ACS-4004
PhosSTOP™	Roche	04906845001
cOmplete™	Roche	04693116001
microBCA kit	Thermo Fisher Scientific	23235
1X Laemli SDS buffer	Thermo	J61337.AC
TGX Stain-Free Precast Gels	Criterion	5678084
5% Skim Milk	Serva	42590.01
Western Lightning Plus Enhanced Chemiluminescence Substrate	PerkinElmer	10400505
PierceTM BCA protein assay kit	Thermo Fisher Scientific	23227
RLT buffer	QIAGEN	79216
RNeasy Mini kit	QIAGEN	74104
RNase-Free DNase Set kit	QIAGEN	79254
iScript	Bio-Rad	1708890
Power SYBR Green Master Mix	Applied Biosystems	4367659
Nunc™ Lab-Tek™ II	Thermo Fisher Scientific	154453
paraformaldehyde	Sigma-Aldrich	30525-89-4
Triton-X100	Sigma-Aldrich	9036-19-5
Fluoromount-G™ with DAPI	Invitrogen	00-4959-52
Click-iT™ EdU Cell Proliferation Kit for Imaging, Alexa Fluor™ 488 dye	Invitrogen	C10337
0.5 M EDTA	Ambion	10458654
BSA fraction V	Roth	No. 0052.2
Ribo-Zero Gold rRNA Depletion Kit	Illumina	20040529
**Software**		
GraphPad Prism 11		
FIJI		
BD FACSDiva Software 8.0.1		
FlowJo 10.10.0		
Flaski	flaski.age.mpg.de	
UCSF ChimeraX 1.6.1		
EVOSTM Analysis 1.4.998.659		
DESeq2 (1.40.2)		
EVOS FL Auto 2 Cell Imaging System		
Image Lab v.6.1		
Skyline Version 22.2.0.527		
RSeQC (v4.0.0)		
DAVID Bioinformatics Resources tool		
Python		
R		
CHOP-CHOP	https://chopchop.cbu.uib.no/	

### Fish husbandry

All experiments were performed on adult (young, aged 7–8 weeks; and old, aged 18–19 weeks) African turquoise killifish *N. furzeri* laboratory strain GRZ-AD. Adult fish were housed individually in 2.8 L tanks from the second week of life. Water parameters were maintained at pH 7–8, kH 3–5, and a temperature of 27.5 °C. The system was automatically replenished with 10% fresh water daily. Fish were raised under 12 h of light and 12 h of darkness and fed with 10 mg of dry pellet (BioMar INICIO Plus G) and Premium Artemia Coppens twice a day, with total amount of daily food delivered equivalent to approximately 2–3% of fish body weight, with the first feeding at 8:30 and the second at 13:30. For tissue collection, fish were euthanized by rapid chilling. Tissues were rapidly dissected, snap-frozen in liquid nitrogen, and stored at −80 °C. For irradiation experiments, fish from each genotype were pooled in a 5 L tank, irradiated with 15 Gy of γ-radiation in a Biobeam 8000 device, and then returned to individually housed tanks. Five days post irradiation, fish were sacrificed for tissue collection. Animal experiments were conducted in accordance with relevant guidelines and approved by the ‘Landesamt für Natur, Umwelt und Verbraucherschutz Nordrhein-Westfalen’ under license number 2023.A109.

### Generation of cGAS KO killifish

CRISPR/Cas9 genome editing was performed as described previously (Harel et al, 2016). The single guide RNA (sgRNA) was designed based on the CHOP-CHOP web-based tool (https://chopchop.cbu.uib.no). Alt-R S.p. HiFi Cas9 Nuclease and the Alt-R sgRNA were purchased from IDT (see the Reagents and Tools Table for sgRNA and primer sequences). One-cell-stage embryos were injected with 1–2 nl of a solution containing Cas9 enzyme (200 ng µl^−1^), sgRNA (20 ng µl^−1^), KCl (0.2 M), and 1% phenol red. The F0 generation was genotyped by fin-clipping to identify potential founders. Selected founders were then backcrossed with the GRZ-AD strain for at least four generations to minimize potential off-target mutations induced by CRISPR editing.

### Establishment of killifish fin fibroblast primary cell cultures

Killifish primary fibroblasts were established as described by Astre et al (Astre et al, 2023) with modifications. Specifically, 10-week-old male WT and cGASa KO killifish were euthanized by rapid chilling and their caudal fins excised, washed twice with Dulbecco’s PBS (DPBS, Gibco), and then transferred under a sterile laminar flow hood. The fins were washed twice more with sterile DPBS, and then disinfected for 10 min with 25 ppm iodine solution (PVP-I, Sigma-Aldrich) in DPBS. The fins were then washed once with DPBS and the samples further sterilized for 2 h in antibiotic solution with Gentamicin (50 µg/ml Gibco) and Primocin® (50 µg/ml InvivoGen) in DPBS at room temperature. The fins were washed again with DPBS and then digested for 20 min in Collagenase P solution (1 mg/ml, Merck Millipore) in Leibovitz’s L-15 medium, 2 mM L-glutamine (Gibco). Without washing, the fins were transferred to culture plates where they were cut into small pieces using sterile scalpels. The tissues were left on the plate with covers open under the hood for 10 min, allowing the digestion solution to evaporate and tissues to adhere to the plate. Plates were then filled with 10 ml L-15 medium supplemented with 15% FBS, Gentamycin (50 µg/ml) and Primocin® (50 µg/ml) and incubated at 28 °C with no addition of CO_2_. Every 4 days, the plates were carefully washed with DPBS to prevent tissue detachment, followed by medium replacement. When cell outgrowth from the tissues became visible, the tissues were removed, and the cells were then detached with Trypsin-EDTA 0.05% (Gibco) and passaged onto a new plate. For the first five passages, cells were grown in media containing Gentamycin and Primocin (50 µg/ml each), and thereafter grown without antibiotics.

### Cell culture maintenance

cGAS KO human THP1 monocytes were purchased from InvivoGen (THP1-Dual™ KO-cGAS Cells). THP1 cells were cultured under 5% CO_2_ at 37 °C in RPMI 1640 (Gibco), 4.5 g/L D-Glucose, 2.383 g/L HEPES, 2 mM L-glutamine, 1.5 g/L Sodium Bicarbonate, 110 mg/L Sodium Pyruvate, 50 µM 2-Mercaptoethanol (Gibco), 20% (v/v) heat-inactivated fetal bovine serum (FBS; 30 min at 56 °C for heat inactivation). Killifish fin fibroblasts were cultured in a humidified incubator at 28 °C with no addition of CO_2_ in Leibovitz’s L-15 medium (Gibco), 2 mM L-glutamine, 15% (v/v) fetal bovine serum.

Both human and killifish cells were repeatedly tested for mycoplasma infection using the Eurofins Genomics Mycoplasmacheck Service covering all mycoplasma species listed in the European Pharmacopoeia 2.6.7.

### Primary cell culture irradiation and senescence-associated β-galactosidase staining

Killifish primary cells were irradiated with 10 Gy of γ-radiation in a Biobeam 8000 device 24 h after the last passage, while still in the medium-filled plate. For senescence-associated β-galactosidase staining, cells were fixed and stained nine days post-irradiation using the Senescence β-Galactosidase Staining Kit (Cell Signaling) according to the manufacturer’s instructions. Images of the cells were taken using an EVOS FL Auto 2 microscope (×20 magnification) and analyzed with the EVOS FL Auto 2 Cell Imaging System software.

### In situ β-galactosidase staining

Kidneys and intestines were harvested from 19-week-old WT and cGAS KO female fish. Tissues were harvested in ice-cold PBS, transferred to ice-cold 10% sucrose for 60 min and then transferred to ice-cold 20% sucrose for another 60 min. Tissues were then transferred to ice-cold 30% sucrose and left overnight. The following day, the tissues were embedded in Tissue-Tek® O.C.T. Compound (SAKURA) and then were frozen at −80 °C until sectioning. The embedded tissues were sectioned using an Epredia CryoStar NX70 Cryostat at 10 μm thickness and mounted in Superfrost Plus slides (Epredia). Slides were air-dried for 60 min, then fixed and stained with buffers from the Senescence β-Galactosidase Staining Kit (Cell Signaling), according to the manufacturer’s instructions.

Images of the tissues were taken using an EVOS FL Auto 2 microscope (×40 magnification) and analyzed with the EVOS FL Auto 2 Cell Imaging System software.

### Plasmids

All plasmids were generated using the pSBbi-GN backbone designed by Eric Kowarz (Kowarz et al, 2015) (Addgene plasmid ID #60517). The kcGASa gene was amplified from cDNA obtained from killifish primary cell cultures using primers with overhangs matching the Gibson Assembly regions on pSBbi-GN (see Reagents and Tools Table for primer sequences). The hcGAS gene was amplified from WT THP1 (InvivoGen) cDNA. kcGASb was chemically synthesized to contain the Gibson-Assembly overhangs flanking the gene and an N-terminal 6xHis-tag. The pSBbi-GN vector was linearized using SfiI (NEB), and the linear plasmid was combined with the amplified cDNAs for Gibson Assembly (NEBuilder® HiFi DNA Assembly, NEB) to make N-terminal 6xHis-tagged constructs. All cloning products used in the experiments have been submitted to Addgene (kcGAS: #225274, hcGAS #225275, kcGAS KO #225276, 6xHis-kcGASa: #259587, 6xHist-kcGASb: #259588).

### Cell culture transfections

THP1 cells were transfected using GeneXPlus (ATCC) according to the manufacturer’s instructions. Forty-eight hours after transfection, cells were split into three equal parts. One part was used for 2’,3’-cGAMP measurements, one part was used for RNA extraction and qPCRs, and one part was used for flow cytometry to assess transfection efficiency.

### Western blot

Forty-eight hours after transfecting HEK293T cells with 6-His-tagged cGAS genes, cells were washed once with PBS and then lysed on the plate with freshly prepared Triton Lysis Buffer (50 mM Tris-HCl pH 7.5, 150 mM NaCl, 1% TritonX-100, 50 Mm NaF, 2 mM Na-vanadate, 1× beta-glycerophosphate, 1× PhosSTOP™ (Roche), and 1× cOmplete™ (Roche). Cells were lysed on the plate for 10 min on ice before being scraped for harvesting. Lysates were centrifuged at 10,000× *g* for 15 min at 4 °C, and the supernatant was collected for downstream protein measurement. Protein concentration was determined using the microBCA kit (Thermo Fisher Scientific). Western blot samples were prepared to contain 10 μg protein per lane in sample in 1× Laemli SDS buffer (Thermo) diluted in ddH_2_O, boiled at 95 °C for 5 min, and then stored at −20 °C until use. Prior to electrophoresis, samples were thawed and equilibrated at room temperature before loading into TGX Stain-Free Precast Gels (Criterion). Samples were run at 120 V for 60 min, and the gel-separated proteins were transferred onto nitrocellulose membranes using the Trans-Blot Turbo Transfer System (Bio-Rad). Membranes were washed once with 1× TBST and then blocked for 60 min with 5% Skim Milk (Serva) in TBST. Membranes were then incubated overnight at 4 °C with 1:1000 anti-6xHis tag antibody (Abcam, [HIS.H8] # ab18184) in 5% BSA fraction V in TBST. The following day, membranes were washed 3× in TBST and then incubated for 60 min at room temperature with anti-mouse secondary antibody (Peroxidase AffiniPure® Donkey Anti-Mouse IgG (H + L), #715-035-151, Jackson ImmunoResearch) 1:5000 in 5% BSA fraction V in TBST. Membranes were washed again 3× with TBST before developing. Western Lightning Plus Enhanced Chemiluminescence Substrate (PerkinElmer) was used as a chemiluminescence reagent, and a ChemiDoc Imager (Bio-Rad, software, Image Lab v.6.1) was used for imaging.

### LC–MS-based targeted metabolomic measurements of cyclic nucleotides

This method was adapted from previously published work (Annibal et al, 2021). Briefly, cells were extracted using a two-phase extraction method with a mixture of DW:MeOH:CHCl3 (1:2:2). After centrifugation, the separated upper and lower phases were dried under a vacuum evaporator and stored at –80 °C until further analysis. The protein content of the remaining cell pellet was used for normalization, determined by the PierceTM BCA protein assay kit (Thermo Fisher Scientific).

Compounds were separated on a reversed-phase column (HSS T3 column 1.8 μm, 100 mm × 2.1 mm, Waters) maintained at 40 °C using the Vanquish UHPLC system (ThermoFisher Scientific) coupled to a triple quadrupole mass spectrometer (TSQ Altis, ThermoFisher Scientific).

The mobile phases consisted of 1% formic acid in H_2_O (A) and 1% formic acid in ACN (B), with a flow rate of 0.3 mL/min. The gradient was set as follows: 1% B was maintained for 1 min, then ramped up to 15% B over 3 min and reached 50% in an additional 1 min. At min 7, it reached 70% B and was maintained for 1.5 min. The gradient was then rapidly decreased to 1% B, and the column was re-equilibrated for 3 min.

2′3′-cGAMP (*m/z* 675.67) was quantified using a fragment ion at *m/z* 476.01 and validated using a confirmatory ion at *m/z* 505.9. The signal was calculated by dividing the peak area by the internal standard peak area (adenosine-13C10,15N5-5′-monophosphate, *m/z* 363.12 ➔ *m/z* 146.08) and further normalized to protein concentration. Data were analyzed using Skyline Version 22.2.0.527.

### RNA extraction and qPCR analysis

Killifish tissues were pulverized in a liquid nitrogen-filled mortar until completely homogenized. The resultant powder was thawed on ice in RLT buffer (QIAGEN) with 1% β-ME v/v. Samples were centrifuged at 10,000 × *g* for 10 min at 4 °C, and the supernatant was collected for subsequent RNA extraction.

As non-adherent cells, THP1 cells were collected by centrifugation at 500 × *g* for 4 min at room temperature. The pellet was washed once with DPBS, snap-frozen in liquid nitrogen, and subsequently, frozen pellets were thawed on ice in RLT buffer (QIAGEN) with 1% β-ME v/v for subsequent RNA extraction.

Nine days post irradiation, killifish fibroblasts in culture dishes were washed with PBS, incubated on ice with RLT buffer (QIAGEN) containing 1% β-ME v/v for 5 min, and then scraped and processed for subsequent RNA extraction.

RNA extraction was performed using the RNeasy Mini kit (QIAGEN), and DNase digestion was carried out using the RNase-Free DNase Set kit (QIAGEN) according to the manufacturer’s instructions. Concentration and purity of the RNA were assessed by NanoDrop spectrophotometry. cDNA synthesis was carried out using iScript (Bio-Rad). Quantitative PCR was performed using Power SYBR Green Master Mix (Applied Biosystems) on a ViiA 7 Real-Time PCR System (Applied Biosystems). Four technical replicates were averaged for each sample and primer pair. *Gapdh* and *Eif3c* were used as internal references for killifish samples; *GAPDH* was used for human THP1 cells (the Reagents and Tools Table provides primer sequences).

### Flow cytometry

THP1 cells were pelleted by centrifugation at 500×*g* for 4 min, washed once with DPBS (Gibco), and pelleted again under the same conditions. Cell pellets were resuspended in cold 2% (v/v) FBS in DPBS and passed through a 35-µm nylon mesh cell strainer to obtain a single cell suspension. DAPI was added at a concentration of 0.1 µg/mL to each sample, and at least 10,000 cells per sample were immediately acquired using a BD FACSCanto II flow cytometer. Data were analyzed using FlowJo (v10.10, BD Biosciences) software. Cells were gated based on forward and side scatter, and doublets were excluded. Transfection efficiency was monitored using GFP co-expression vectors (Fig. EV1F). Gates for GFP-positive cells were defined relative to untransfected THP1 cells. Gates for DAPI-positive cells were defined using samples without DAPI. Compensation between DAPI and GFP was performed using single-color controls.

### γH2AX staining

Killifish cells were plated on glass slide chambers (Nunc™ Lab-Tek™ II) and allowed to attach for 24 h. At the 24 h timepoint, chambers were irradiated with 10 Gy of γ-radiation in a Biobeam 8000 device and returned to a humidified incubator at 28 °C without CO_2_ supplementation. After completion of all timepoints (0 h non-irradiated, 2, 4, 8 h after irradiation), slides were processed together at room temperature unless otherwise indicated. Slides were washed once with DPBS (Gibco) and fixed in freshly prepared 4% (w/v) paraformaldehyde (Sigma-Aldrich) in PBS (pH 7.2) for 15 min. Following 3 washes with DPBS, cells were permeabilized with 0.5% (v/v) Triton-X100 (Sigma-Aldrich) in DPBS for 10 min, washed an additional 3 times, and blocked for 1 h in 8% (v/v) FBS, 0.3% (v/v) Triton-X100 in DPBS. Primary antibody against γH2AX (#9718 Cell Signaling) was applied at 1:200 dilution in 5% (v/v) FBS, 0.1% (v/v) Triton-X100 in DPBS and incubated overnight at 4 °C. Slides were washed three times with DPBS and then incubated for 1 h with fluorescence-conjugated secondary antibody (#A21206, Invitrogen) diluted 1:1000 in 5% (v/v) FBS, 0.1% (v/v) Triton-X100 in DPBS. Slides were washed 3 times and mounted using Fluoromount-G™ with DAPI (Invitrogen). Images were acquired using a Leica DMI6000 B microscope and analyzed with FIJI software. A stringent intensity threshold was set such that only intense, distinct puncta were counted. Images with distinct puncta were chosen randomly to define the threshold, which was then applied across all images. Only objects with a size of 1–50 pixels were counted per nucleus to exclude signal aggregates. At least 50 cells per slide were analyzed.

### EdU staining

For in vitro experiments, cultured killifish cells were plated on autoclaved glass coverslips and allowed to attach overnight. After 24 h, cells were irradiated with 10 Gy of γ-radiation in a Biobeam 8000 device and returned to a humidified incubator at 28 °C without CO_2_ supplementation. Eight days later, non-irradiated replicating cells were plated in parallel to serve as controls. Nine days post-irradiation, all cells were fixed and stained using reagents from the Click-iT™ EdU Cell Proliferation Kit for Imaging, Alexa Fluor™ 488 dye (Invitrogen), according to the manufacturer’s instructions.

For in vivo EdU labeling, 19-week-old WT and cGAS KO killifish were intraperitoneally injected with 8 μg/g body weight of filter-sterilized EdU in PBS. Six hours post-injection, kidneys and intestines were harvested in ice-cold PBS and cryoprotected by serially incubating in increasing concentrations of ice-cold sucrose solutions (10%, 20%, 30% w/v in PBS). Tissues were incubated 60 min each in 10% and 20% sucrose, and then held in ice-cold 30% sucrose overnight. The following day, tissues were embedded in Tissue-Tek® O.C.T. Compound (SAKURA), frozen at −80 °C, and then sectioned at 10 μm thickness using an Epredia CryoStar NX70 Cryostat. Sections were mounted on Superfrost Plus slides (Epredia). Slides were air-dried for 60 min, then fixed and stained using reagents from the kit Click-iT™ EdU Cell Proliferation Kit for Imaging, Alexa Fluor™ 488 dye (Invitrogen), according to the manufacturer’s instructions.

For both in vitro and in vivo experiments, stained slides were mounted using Fluoromount-G™ with DAPI (Invitrogen), images acquired using a Leica DMI6000 B microscope, and analyzed using FIJI software. A stringency threshold was set to identify EdU-positive cells. The image with the highest signal intensity was used to define the threshold, which was applied across all images. Following thresholding, watershed segmentation was applied to separate closely apposed cells, and all objects were quantified.

### LCP1 and PCNA immunofluorescence staining

Eighteen-week-old male WT and cGAS KO fish were euthanized and fixed in 10% Formalin at 4 °C for 72 h. Fish were subsequently decalcified in 0.5 M EDTA (Ambion) at 4 °C for an additional 72 h, and transferred back to 10% Formalin and processed for paraffin infiltration using the Paraffin Infiltrator Donatello. Immediately thereafter, fish were embedded in paraffin blocks using the Thermo HistoStar Embedding Workstation, and the blocks were stored at room temperature. Sagittal sections 4 μm thick were cut from the entire fish body using a Thermo Scientific Microm HM 340E electronic rotary microtome and sections attached to Superfrost Plus slides (Epredia). Slides were air-dried overnight at 37 °C and stored at room temperature.

Prior to staining, slides were deparaffinized using a Thermo Scientific Gemini AS automated slide stainer. Antigen retrieval was performed in pH 6 Citrate Buffer using a 2100 Antigen Retriever (Aptum). Slides were washed 3× in PBS (Gibco) + 0.3% TritonX-100 (Sigma), blocked for 1 h in a solution containing 5% (w/v) BSA fraction V (Roth) and 2% (v/v) FBS (Gibco) in PBS. Primary antibodies were applied overnight at 4 °C using anti-LCP1 (GeneTex, Cat. No.: GTX124420) at 1:500 and anti-PCNA ([PC10] (ab29), Abcam) at 1:10,000 in 5% BSA in PBST. The following day, slides were washed 3× in PBST and incubated for 1 h at room temperature with secondary antibodies: 1:500 anti-Rabbit IgG - Alexa Fluor™ 488 (Invitrogen, Catalog # A-21206) and 1:500 anti-Mouse IgG - Alexa Fluor™ 568 (Invitrogen, Catalog # A10037) in 5% BSA in PBST solution. Slides were washed 3× in PBS without TritonX-100 before mounting using Fluoromount-G™ with DAPI (Invitrogen). Slides were imaged using a Leica DMI6000 B microscope (×40 magnification and analyzed with FIJI software. A stringency threshold was set such that only intense and separate cells were counted. An image with the most intense puncta was selected to set the threshold, which was then applied across all images. After thresholding, watershed segmentation was used to separate proximal cells, and then all objects were quantified.

### RNA sequencing analysis

Irradiated and non-irradiated primary fibroblasts, as well as kidneys and guts (stomach and intestine) from WT and cGAS KO fish, were collected for RNAseq analysis. All in vitro samples were processed in one batch, and all in vivo samples were processed in another. Both WT and cGAS KO animals were derived from offspring of heterozygous parents to ensure comparable genetic backgrounds. To minimize circadian variation, fish were euthanized within a 2 h window in the early afternoon. Samples were snap-frozen in liquid nitrogen and stored at −80 °C. RNA extraction of all samples within each batch was performed simultaneously.

Frozen tissues were pulverized in a mortar in liquid nitrogen, and the resultant powder was thawed on ice in RLT buffer (QIAGEN) containing 1% β-ME v/v. RNA extraction was performed using the RNeasy Mini Kit (QIAGEN), followed by DNA digestion using the RNase-Free DNase Set kit (QIAGEN), according to the manufacturer’s instructions.

Libraries were prepared from 500 ng of total RNA. ERCC RNA Spike-In Mix 1 (Thermo Fischer) was added to the samples prior to library preparation. Ribosomal RNA was depleted enzymatically using the Ribo-Zero Gold rRNA Depletion Kit (Illumina), followed by library construction using a TruSeq Stranded Total RNA sample preparation Kit (Illumina). Remaining RNA was fragmented and reverse-transcribed with random hexamer primers. Second-strand synthesis incorporating dUTPs was followed by A-tailing, adapter ligation, and PCR amplification for 15 cycles. Libraries were validated and quantified using Tape Station (Agilent), pooled in equimolar amounts, and then quantified using the Peqlab KAPA Library Quantification Kit and the Applied Biosystems 7900HT Sequence Detection System. Samples were sequenced on an Illumina NovaSeq6000 platform using paired-end 100 bp reads targeting 50 million clusters per sample.

After removal of residual rRNA and tRNA reads, remaining reads were pseudo-aligned to the reference genome (Nfu_20140520) using Kallisto (v.0.45.0) (Bray et al, 2016). Mapping strand specificity was assessed using RSeQC (v4.0.0) (Wang et al, 2012b). Samples with >60% of reads mapping to a single strand were classified as stranded, whereas samples with <60% strand bias were classified as unstranded. Genes with fewer than ten total reads were excluded from analysis. Read counts were normalized using the median-of-ratios method, and pairwise differential gene expression analysis was performed using DESeq2 (v1.24.0) (Love et al, 2014), with log₂ fold changes shrunk using approximate posterior estimation for generalized linear model coefficients. In the in vitro RNAseq data, we also added the interaction term genotype x treatment to DESeq2, with the complete model including pairwise comparisons being _~_ genotype + treatment + genotype x treatment.

Gene set enrichment analysis (GSEA) (Mootha et al, 2003; Subramanian et al, 2005) and principal component analysis (PCA) were performed using Flaski (https://flaski.age.mpg.de/). KEGG pathway enrichment analysis for in vitro datasets was performed using the DAVID Bioinformatics Resources tool.

### Lifespan analysis

P0 heterozygous kcGAS KO/WT fish were crossed to generate F1 animals, which served as parents for all F2 fish used in lifespan analyses. The experimental cohort for lifespan was derived from eggs collected within a span of 10 days. After hatching, larvae were group-housed (seven larvae per 1.1 L tank), until they reached 2 weeks of age, and subsequently individually housed in 2.8 L tanks for their remaining lifespan. Mortality scoring started at the sixth week, corresponding to full sexual maturation. At this stage, no fish displayed abnormalities in body size or swimming ability, and no fish were censored from the study. Aged fish (> 28 weeks) exhibiting severe stress, lethargy, anorexia, or abnormal swimming behavior were sacrificed to ensure animal welfare. Age at euthanasia was recorded as the estimated time of natural death. Survival curves were generated using the Kaplan–Meier estimator and statistical significance determined using the Mantel–Cox log-rank test.

### Tissue fixation, H&E staining, and acid-fast bacteria staining

Fish within the lifespan cohorts were monitored four times daily to assess viability. Upon detection of death, fish were collected, their visceral cavities opened, and the specimens were fixed in 10% Formalin at room temperature. Fish with severe post-mortem autolysis (due to 16–24 h delay between death and detection) were not included in the study. Fish were stored in Formalin until the lifespan experiment was completed. In total, 141 samples were then submitted to IDEXX BioAnalytics for processing and histopathologic evaluation. Briefly, all fish were paraffin-embedded, and four sections were taken from each fish. After deparaffinization, the sections were processed for either H&E or acid-fast bacteria (AFB) staining. Lesion presence or absence was recorded across a standard tissue panel, including brain, epidermis, gills, heart, digestive tract (headgut, foregut, midgut, hindgut), kidney, liver, gonads (ovary or testes), skeletal muscle, spinal cord, swim bladder, and vertebral column. Lesions recorded included neoplastic, proliferative but non-neoplastic, degenerative, non-infectious inflammatory, and infectious inflammatory.

### Multivariate logistic regression analysis of pathological phenotypes

To assess the effect of cGAS knockout on pathological phenotypes, post-mortem histopathological data were analyzed from 60 WT and 47 cGAS KO fish. Pathologies were grouped into four non-neoplastic categories: proliferative, degenerative, infectious, inflammatory, and non-infectious inflammatory. Each outcome was treated as a binary variable (1 = pathology present, 0 = pathology absent).

Genotype was coded with WT as the reference group and cGAS KO as the experimental group. Sex was coded with males as the reference category and females as the comparison group. The effect of genotype and sex on pathology risk was assessed using a generalized linear model with a binomial distribution and logit link function. Age at death (in days) was included as a continuous covariate. The GLM model fitting was performed using the statsmodels library in Python. The full model was specified as:

$${{\bf{Pathology}}} \sim {{\bf{Genotype}}}+{{\bf{Sex}}}+{{\bf{Age}}}+{{\bf{Genotype}}}:{{\bf{Sex}}}$$

For sex-specific organs (ovary and testis), analyses were stratified by sex, and models included genotype and age as predictors (Pathology ~ Genotype + Age).

Results are reported as odds ratios (ORs) with corresponding 95% CIs. The null effect was defined as OR = 1.0. The genotype main-effect OR represents the effect of cGAS KO relative to WT in males (reference sex). The *P* value for this effect was obtained directly from the standard model output. The genotype-by-sex interaction OR quantifies the multiplicative factor modification of the genotype effect in females. The total effect of cGAS KO in females was calculated as the product of the genotype main-effect OR and the genotype x Sex interaction OR. Statistical significance for the Total Female Effect was determined using the Wald test on the linear hypothesis (beta(Genotype) + beta(Interaction) = 0). The main results report the main effect (male) and the total female effect with the corresponding statistical significance score.

## Supplementary information

Peer Review File

Source data Fig. 1

Source data Fig. 2

Source data Fig. 3

Source data Fig. 4

Source data Fig. 5

Expanded View Figures

## Data Availability

All raw in vivo RNAseq data are available in the SRA database under BioProject ID PRJNA1154547. The source data of this paper are collected in the following database record: biostudies:S-SCDT-10_1038-S44319-026-00917-y.
