# Supplementary material for: Loss of killifish cGAS attenuates age-related signatures but does not affect organismal life span
Source: EMBO Rep. 2026 Sep 9;27(20):5723–51. doi: 10.1038/s44319-026-00917-y (PMC13694710; doi:10.1038/s44319-026-00917-y)
Supplement: Supplementary file 1 [file 44319_2026_917_MOESM1_ESM.pdf]

EMBO reports

Loss of killifish cGAS attenuates age-related signatures but does not affect organismal life span

Eugen Ballhysa, Roberto Ripa, Nadine Hochhard, Tin Tin Nguyen, Jennifer Brazzell, Youngjun Park, Baptiste Ferreri, Elena Hoffmann, Raymond Laboy, Joachim Steiner, and Adam Antebi

Corresponding author(s): Adam Antebi (AAntebi@age.mpg.de)

Review Timeline:

Submission Date:	12th May 26
Editorial Decision:	18th May 26
Revision Received:	2nd Jul 26
Editorial Decision:	29th Jul 26
Revision Received:	8th Aug 26
Accepted:	21st Aug 26

Editor: Achim Breiling / Martina Rembold

Transaction Report: This manuscript was submitted to EMBO Reports following peer review at The EMBO Journal.

(Note: With the exception of the correction of typographical or spelling errors that could be a source of ambiguity, letters and reports are not edited. Depending on transfer agreements, referee reports obtained elsewhere may or may not be included in this compilation. Referee reports are anonymous unless the Referee chooses to sign their reports.)

Dear Dr. Antebi

Thank you for the submission of your manuscript to EMBO reports, that recently has been rejected by The EMBO Journal post arbitration. I now went again through the manuscript and the arbitration reports from The EMBO Journal (attached also below) and your p-b-p-response (further revision plan).

Judging from your revision plan, it seems that the points of the arbitrators, in particular of arbitrator #2, will be adequately addressed during a further revision. I thus invite you to revise your manuscript accordingly with the understanding that the referee concerns must be addressed in the revised manuscript and/or in a detailed further point-by-point response, as indicated in your revision plan.

Acceptance of your manuscript will depend on a positive outcome of a final round of review involving arbitrator #1. Revised manuscripts should be submitted within three months of a request for revision. Please contact me to discuss the revision (also by video chat) if you have questions or comments regarding the revision, or should you need additional time.

Moreover, your manuscript needs formatting to our journal style. Please carefully review the instructions that follow below.

PLEASE NOTE THAT upon resubmission revised manuscripts are subjected to an initial quality control prior to exposition to re-review. Upon failure in the initial quality control, the manuscripts are sent back to the authors, which may lead to delays. Frequent reasons for such a failure are the lack of the data availability section (please see below) and the presence of statistics based on n=2 (the authors are then asked to present scatter plots or provide more data points).

When submitting your revised manuscript, we will require:

1) a .docx formatted version of the final manuscript text (including legends for main figures, EV figures and tables), but without the figures included. Figure legends should be compiled at the end of the manuscript text.

2) individual production quality figure files as .eps, .tif, .jpg (one file per figure), of main figures and EV figures. Please upload these as separate, individual files upon re-submission.

The Expanded View format, which will be displayed in the main HTML of the paper in a collapsible format, has replaced the Supplementary information. You can submit up to 6 images as Expanded View. Please follow the nomenclature Figure EV1, Figure EV2 etc. The figure legend for these should be included in the main manuscript document file in a section called Expanded View Figure Legends after the main Figure Legends section. Additional Supplementary material should be supplied as a single pdf file labeled Appendix. The Appendix should have page numbers and needs to include a table of content on the first page (with page numbers) and legends for all content. Please follow the nomenclature Appendix Figure Sx, Appendix Table Sx etc. throughout the text, and also label the figures and tables according to this nomenclature.

For more details, please refer to our guide to authors:
https://link.springer.com/journal/44319/submission-guidelines#cms-Revised-submissions

Please consult our guide for figure preparation:
https://media.springernature.com/original/springer-cms/rest/v1/content/27825798/data/v1

3) a .docx formatted letter INCLUDING the reports from the arbitrators and your detailed point-by-point responses to their comments. As part of the EMBO Press transparent editorial process, the point-by-point response is part of the Review Process File (RPF), which will be published alongside your paper.

4) a complete author checklist, which you can download from our author guidelines (https://link.springer.com/journal/44319/submission-guidelines#cms-Revised-submissions). Please insert page numbers in the checklist to indicate where the requested information can be found in the manuscript. The completed author checklist will also be part of the RPF.

Please also follow our guidelines for the use of living organisms, and the respective reporting guidelines:
https://link.springer.com/partners/embo-press/editorial-policies#Animal%20and%20human%20studies

5) that primary datasets produced in this study (e.g. RNA-seq, ChIP-seq, structural and array data) are deposited in an appropriate public database. If no primary datasets have been deposited, please also state this in a dedicated section (e.g. 'No primary datasets have been generated and deposited'), see below.

See also: https://link.springer.com/partners/embo-press/editorial-policies#Data%20deposition

Please remember to provide a reviewer password if the datasets are not yet public.

The accession numbers and database should be listed in a formal "Data Availability" section that follows the model below. This is now mandatory (like the COI statement). Please note that the Data Availability Section is restricted to new primary data that are part of this study. This section is mandatory. As indicated above, if no primary datasets have been deposited, please state this in this section

Data availability

The datasets produced in this study are available in the following databases:

- RNA-Seq data: Gene Expression Omnibus GSE46843 (https://www.ncbi.nlm.nih.gov/geo/query/acc.cgi?acc=GSE46843)
- [data type]: [name of the resource] [accession number/identifier/doi] ([URL or identifiers.org/DATABASE:ACCESSION])

*** Note - All links should resolve to a page where the data can be accessed. ***

See also: https://link.springer.com/partners/embo-press/editorial-policies#Data%20availability%20statement

Moreover, I have these editorial requests:

6) We now request the publication of original source data with the aim of making primary data more accessible and transparent to the reader. You will receive a separate email with instructions for providing source data with your revised manuscript, including information how to upload and organize the files.

7) Our journal encourages inclusion of *data citations in the reference list* to directly cite datasets that were re-used and obtained from public databases. Data citations in the article text are distinct from normal bibliographical citations and should directly link to the database records from which the data can be accessed. In the main text, data citations are formatted as follows: "Data ref: Smith et al, 2001" or "Data ref: NCBI Sequence Read Archive PRJNA342805, 2017". In the Reference list, data citations must be labeled with "[DATASET]". A data reference must provide the database name, accession number/identifiers and a resolvable link to the landing page from which the data can be accessed at the end of the reference. Further instructions are available at: https://link.springer.com/journal/44319/submission-guidelines#cms-Reference-guidelines

8) Regarding data quantification and statistics, please make sure that the number "n" for how many independent experiments were performed, their nature (biological versus technical replicates), the bars and error bars (e.g. SEM, SD) and the test used to calculate p-values is indicated in the respective figure legends (also for EV and Appendix figures). Please provide statistical testing where applicable. Please avoid the phrase 'independent experiment' but clearly state if these were biological or technical replicates. Please also indicate (e.g. with n.s.) if testing was performed, but the differences are not significant. In case n=2, please show the data as separate datapoints without error bars and statistics. See also: https://link.springer.com/journal/44319/submission-guidelines#cms-Figure-and-data-presentation

If n<5, please show single datapoints for diagrams.

9) Please add scale bars of similar style and thickness to all microscopic images, using clearly visible black or white bars (depending on the background). Please place these in the lower right corner of the images themselves. Please do not write on or near the bars in the image but define the size in the respective figure legend.

10) Please use our reference format:
https://link.springer.com/journal/44319/submission-guidelines#cms-Reference-guidelines

11) We updated our journal's competing interests policy in January 2022 and request authors to consider both actual and perceived competing interests. Please name this section 'Disclosure and Competing Interests Statement' and put it after the Acknowledgements section.
See: https://link.springer.com/partners/embo-press/editorial-policies#Competing%20interest%20disclosures

12) We now use CRediT to specify the contributions of each author in the journal submission system. CRediT replaces the author contribution section. Please use the free text box to provide more detailed descriptions and do NOT provide your final manuscript text file with an author contributions section. See also our guide to authors (section 'Author contributions'): https://link.springer.com/journal/44319/submission-guidelines#cms-Revised-submissions

13) All Materials and Methods need to be described in the main text using our 'Structured Methods' format, which is required for all research articles. According to this format, the Methods section should include a Reagents and Tools Table (listing key reagents, experimental models, software, and relevant equipment and including their sources and relevant identifiers), uploaded as separate file, and a Methods section in which we encourage the authors to describe their methods using a step-by-step protocol format with bullet points, to facilitate the adoption of the methodologies across labs. More information on how to adhere

to this format as well as downloadable templates (.doc) for the Reagents and Tools Table can be found in our author guidelines (section 'Structured Methods'):

https://link.springer.com/journal/44319/submission-guidelines#cms-Manuscript-organisation-and-formatting

Please add primer or antibody information directly to the Reagents and Tools table.

14) Please order the manuscript sections like this, using only these names:
Title page - Abstract (max. 175 words) - Keywords (max. 5) - Introduction - Results - Discussion - Methods - Data availability section - Acknowledgements (please include here all the funding information) - Disclosure and Competing Interests Statement - References - Figure legends - Expanded View Figure legends

15) Please make sure that all the funding information is also entered into the online submission system and that it is complete and similar to the one in the acknowledgement section of the manuscript text file.

We would also welcome the submission of cover suggestions, or motifs to be used by our Graphics Illustrator in designing a cover.

As part of the EMBO publication's Transparent Editorial Process, EMBO reports publishes online a Review Process File (RPF) to accompany accepted manuscripts. This File will be published in conjunction with your paper and will include the referee reports, your point-by-point response and all pertinent correspondence relating to the manuscript.

You are able to opt out of this by letting the editorial office know (emboreports@embo.org). If you do opt out, the Review Process File link will point to the following statement: "No Review Process File is available with this article, as the authors have chosen not to make the review process public in this case."

I look forward to seeing a revised form of your manuscript when it is ready.

Kind regards,

Achim

Achim Breiling
Senior Editor
EMBO Reports

Arbitrator #1:

Ballhysa et al. generated cGAS KO killifish and characterized their aging. This was done primarily through RNAseq analysis of the kidney and the intestine. It is unclear why the authors selected the kidney and the gut as their tissues of interest; considering cGAS/STING overactivity has been implicated in pathologies affecting the brain, lung, and liver among others. The results appear contradictory.

Kidney RNAseq analysis from young males reveals young cGAS KO fish are significantly more inflamed than WT males. The authors also demonstrate that cGAS KO cells display higher incidence of DNA damage at basal conditions and under irradiation. However, analysis of RNAseq from old WT and KO fish does not mention any findings of inflammatory genes. What does the inflammatory landscape in aged fish look like? Why do the old WT fish appear to lack induction of inflammation compared to the young fish?

Interest in targeting the cGAS/STING pathway in the context of aging stems from consistent findings across taxa (including killifish) that inflammation increases with age, but the authors do not show this. This again calls into question the decision to probe kidney to assess the outcomes of these fish. Finally, the authors do not explain how it is possible for cGAS KO fish to have a clear inflammatory phenotype at a young age along with DNA damage that not only has no effect on lifespan but is lost with age. Is this a biological phenomenon or an experimental artifact?

A recent article published on cGAS KO killifish in Genes & Development (https://genesdev.cshlp.org/content/early/2026/04/13/gad.352760.125) shows that cGAS KO killifish cells have disrupted H3K9me3 organization, as does a preprint on cGAS KO mice (https://pmc.ncbi.nlm.nih.gov/articles/PMC11482887/). This could help explain the rise in inflammation and gH2AX reported by the authors in their young cGAS KO fish, but not their aging data. The aging data is weak and needs to be either expanded to include inflammation outcomes, other tissues outside of the kidney,

or removed. The phenotypic findings from young fish and in vitro are more interesting, and the authors could focus more on expanding their characterization of cGAS KO cells in fish under basal conditions as they already see a difference before irradiation. Without these changes, the manuscript reads as contradictory and without a clear message.

Arbitrator #2:

Overall, the study is technically solid and presents a consistent set of results across in vitro and in vivo systems. The main finding that loss of cGAS reduces senescence and aging-related features but does not extend lifespan is well supported and interesting. The work also makes a useful conceptual point, as it challenges the idea that reducing cGAS/STING-driven inflammation will necessarily increase lifespan. The use of a vertebrate aging model and inclusion of lifespan data strengthen this. At the same time, the study is somewhat limited in mechanistic depth. Much of the evidence is based on transcriptomic and pathway-level analyses, with limited direct functional insight. In particular, the lack of effect on lifespan is not mechanistically explained and remains rather speculative. Some in vivo analyses are also restricted to specific tissues and are not always fully conclusive. Nevertheless, I believe that the study is sufficiently robust and relevant to be considered further provided that the conclusions are kept within the limits of the data.

Arbitrating advisor #1's comments:

Ballhysa et al. generated cGAS KO killifish and characterized their aging. This was done primarily through RNAseq analysis of the kidney and the intestine.

We thank the advisor for their detailed evaluation and the opportunity to clarify our work.

We respectfully disagree that our manuscript is primarily descriptive or limited in scope. While transcriptomic analyses form an important component, they serve to guide downstream physiological investigation. In our study, RNA-seq identified differences in senescence-associated pathways and proliferation dynamics, which we followed up with histological analyses (Figure 5) quantifying senescence, proliferation, and immune infiltration.

In addition, we performed:

- Lifespan analyses in a large cohort of both sexes
- Necropsy across 11 tissues to identify subtle physiological changes
- Multi-tissue histological assessments (kidney, intestine, liver, gonads)

We therefore believe the current phrasing underestimates both the breadth and integration of the dataset.

It is unclear why the authors selected the kidney and the gut as their tissues of interest; considering cGAS/STING overactivity has been implicated in pathologies affecting the brain, lung, and liver among others. The results appear contradictory.

The selection of kidney and intestine was hypothesis-driven and biologically motivated, and justified in the main text.

The kidney in teleosts functions as a primary lymphoid organ and exhibits high proliferative activity. Given the established link between cGAS and senescence, we hypothesized that this tissue would be particularly sensitive to cGAS loss, which our data support. Due to its core function in immunity, the kidney also has high levels of cGAS expression (Figure 1).

To mitigate single-tissue bias, we extended analyses to the intestine, a barrier tissue with strong innate immune activity. Given that cGAS/STING is a central innate immune pathway, examining its role in a pathogen-exposed tissue is both logical and relevant, though it has lower expression (Figure 1).

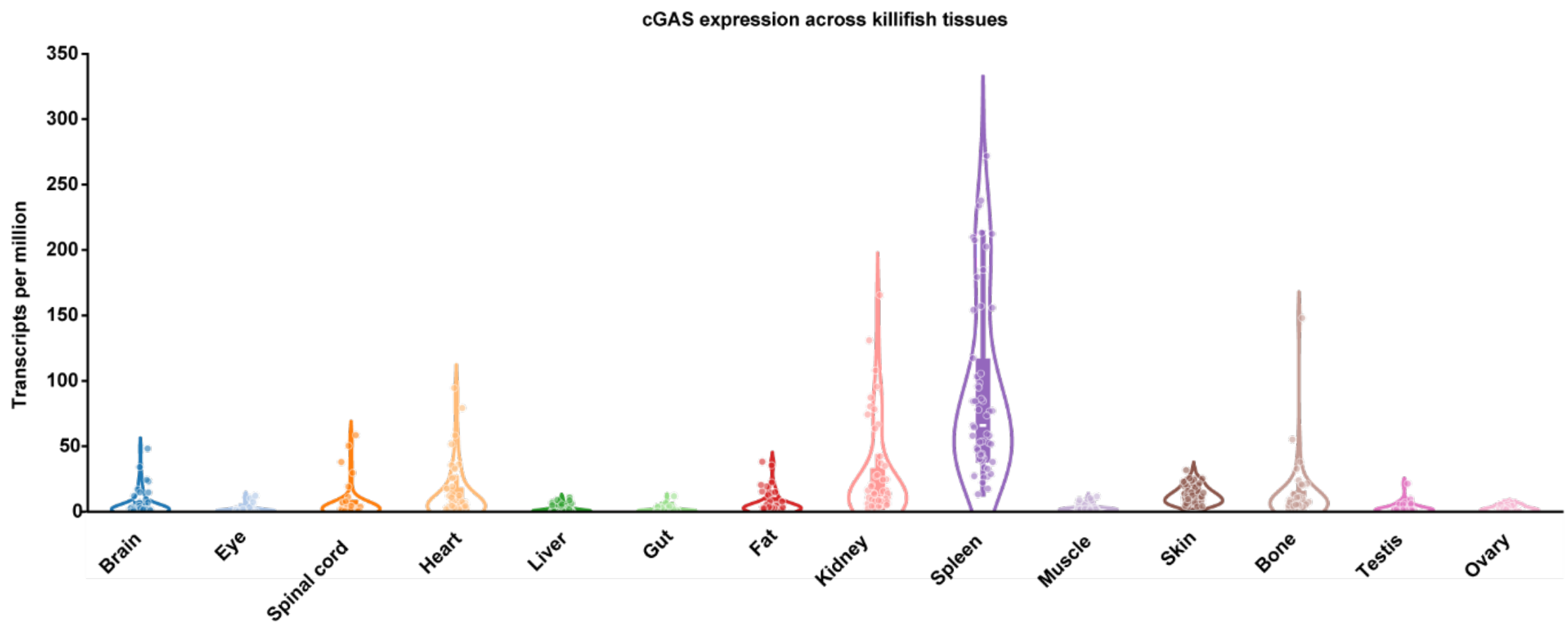

Figure 1. Transcripts per million of cGAS gene expression across killifish tissues. Expression data were obtained from Costa E. et al. [1].

To clarify this point for future readers, we have also included the above panel in Expanded View Figure 2 in response to the reviewer's comment.

Importantly, both tissues are well represented in the cGAS/STING literature:

- cGAS overactivation contributes to kidney pathology, including atrophy and acute injury [2, 3]
- STING inhibition reduces inflammatory signatures in aged kidneys [4]
- cGAS/STING signaling plays context-dependent roles in intestinal inflammation [5]

We can add these references to main text to strengthen our case.

Finally, our study is not restricted to these tissues: additional physiological analyses include liver, gonads, and a broad necropsy panel of 11 tissues.

Kidney RNAseq analysis from young males reveals young cGAS KO fish are significantly more inflamed than WT males. The authors also demonstrate that cGAS KO cells display higher incidence of DNA damage at basal conditions and under irradiation. However, analysis of RNAseq from old WT and KO fish does not mention any findings of inflammatory genes. What does the inflammatory landscape in aged fish look like? Why do the old WT fish appear to lack induction of inflammation compared to the young fish?

We appreciate the opportunity to clarify this point.

In young fish, inflammatory changes are subtle and detectable primarily at the pathway level (via GSEA), reflecting low variance between samples. Individual genes are not significantly altered, but coordinated pathway-level upregulation of the cGAS/STING signaling is evident, presumably reflecting compensation. Notably, the

Even so, we acknowledge that the effects are small and we.take great care to point this out in the text.

In older fish, increased biological variability reduces statistical power to detect the same subtle signatures. However, inflammation is clearly present and consistent with established aging signatures:

- The complement and coagulation cascade is the most upregulated pathway in aged kidneys.
- This pathway is a known hallmark of inflammaging in killifish and conserved across species[1, 6, 7]

Importantly, loss of cGAS blunts this age-associated inflammatory upregulation, supporting a role for cGAS in inflammaging.

Thus, inflammation is not absent in aged fish; rather, it manifests in a manner consistent with known aging biology and dataset-specific variance constraints.

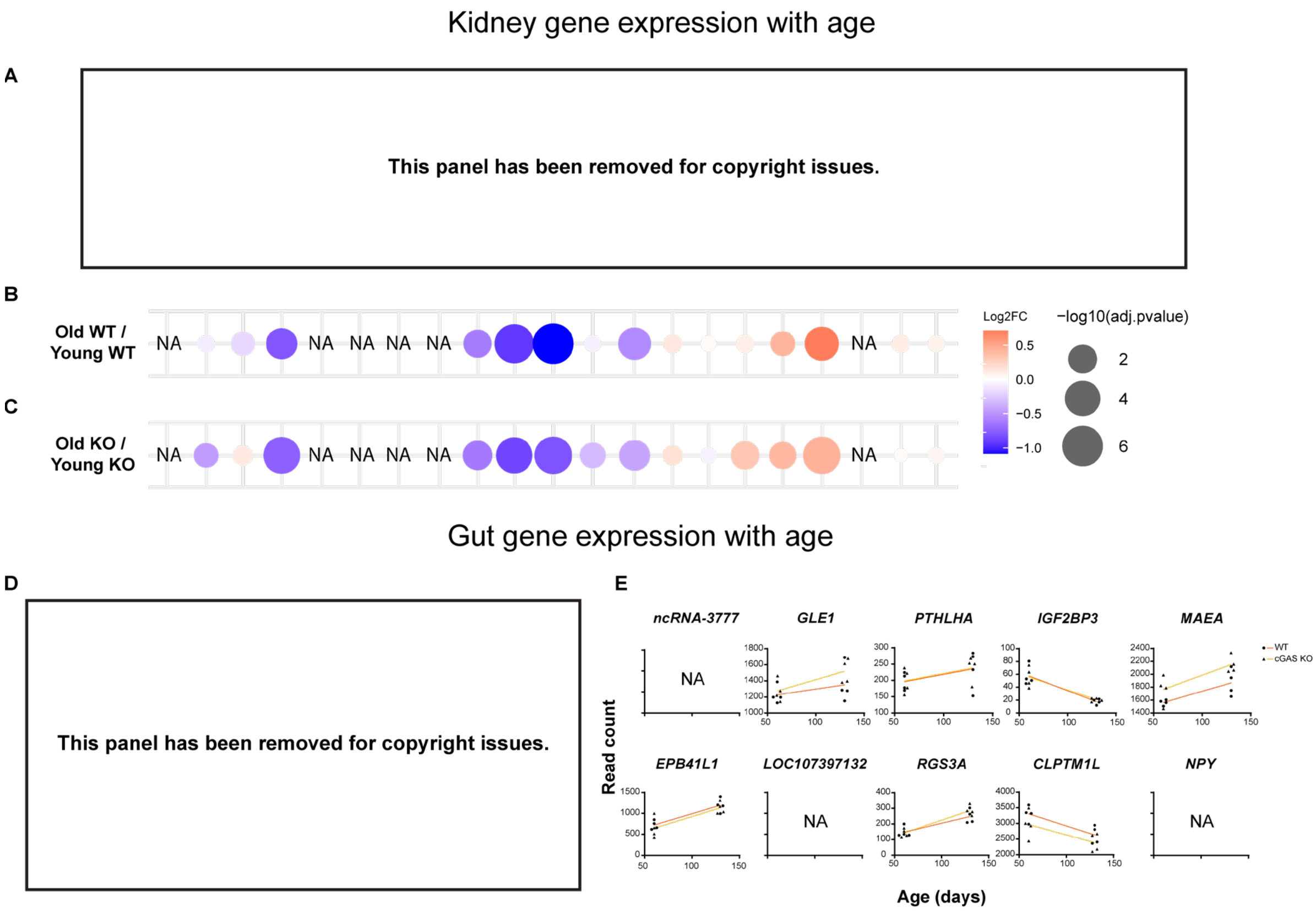

Figure 2. Kidney and gut gene expression with age. A, A modified version of panel 4b from PMID 41776309 (Costa, E.K., et al., Multi-tissue transcriptomic aging atlas reveals predictive aging biomarkers in the killifish. Nat Aging, 2026. 6(3): p. 636–664) was shown here [1, 6]. **B**, Expression changes of the same genes as in (A) comparing young and old WT killifish kidneys from our data. NA= not found in our analysis due to annotation discrepancies between Ncbi and Ensembl databases.

Gene regulation appears consistent. **C**, Expression changes of the same genes as in (A) comparing young and old cGAS KO killifish kidneys in our data. Lack of cGAS leads to increased T-cell marker CD3E as well as blunting of Lymphoid marker RF4A and macrophage marker CSF1RA. These markers are affected by cGAS loss, albeit not to the same extent as complement and coagulation cascade genes. **D**, A modified version of panel 5f from PMID 41776309 (Costa, E.K., et al., Multi-tissue transcriptomic aging atlas reveals predictive aging biomarkers in the killifish. Nat Aging, 2026. 6(3): p. 636–664) was shown here. **E**, Genes from (**D**) expressed in our data as read counts in young (60 days) and old (130 days) WT fish. WT fish gene expressions are shown in circles and the linear regression with age is shown in red. cGAS KO fish gene expressions are shown in triangles and the linear regression with age is shown in orange. The only discrepancy is CLPTM1L, which can be attributed to gene regulation in females, which is absent in our analysis. NA = genes not found in our analysis.

Interest in targeting the cGAS/STING pathway in the context of aging stems from consistent findings across taxa (including killifish) that inflammation increases with age, but the authors do not show this. This again calls into question the decision to probe kidney to assess the outcomes of these fish.

As mentioned above, inflammation is the most upregulated pathway with age, especially in the kidneys (Figure 3).

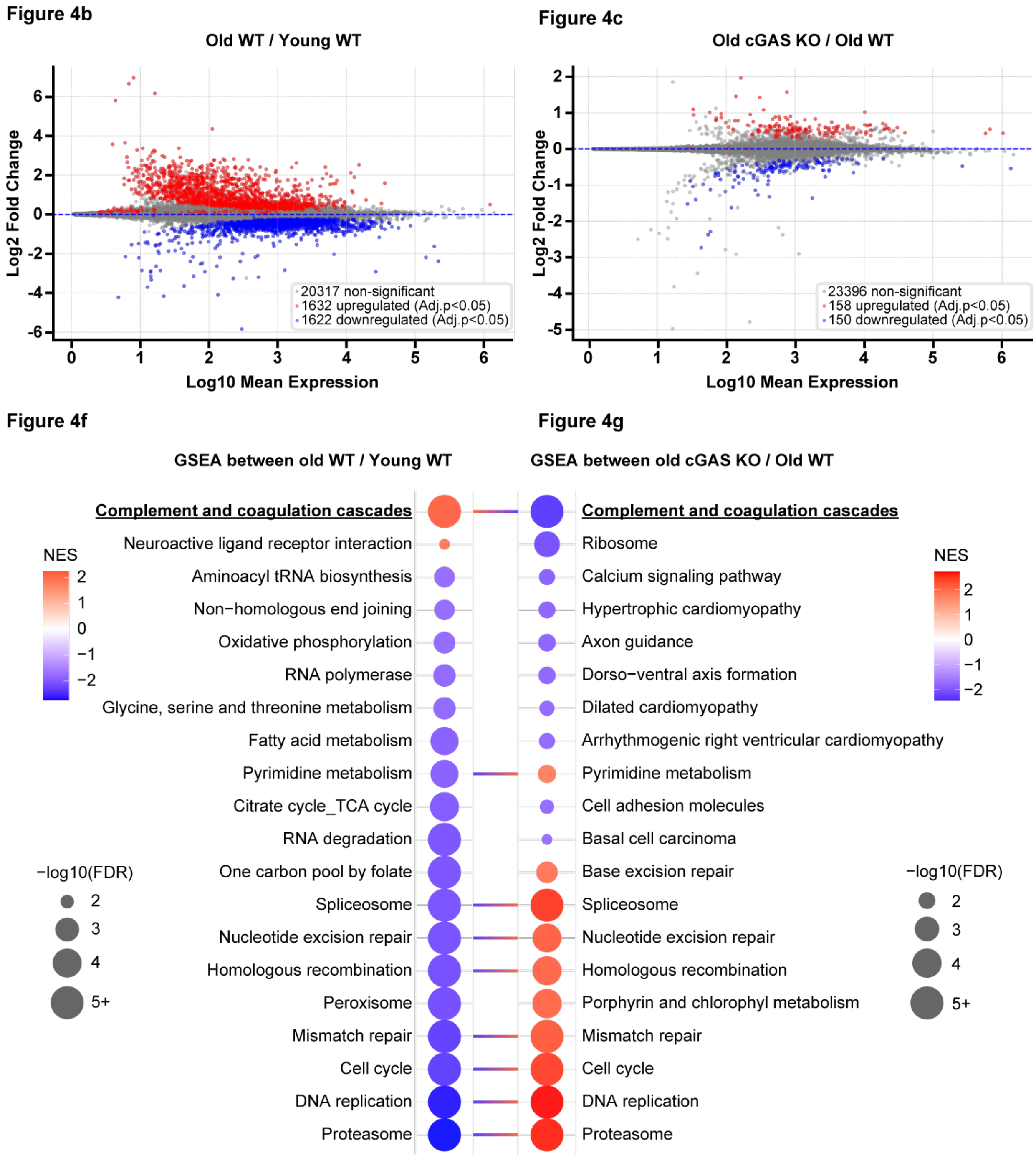

Figure 3. RNAseq analysis modified from main text Figure 4. The data show that with age, complement and coagulation cascades, an immune pathway associated with inflammaging in teleosts and humans, is the most upregulated pathway with age. Lack of cGAS leads to significant mitigation of this upregulation.

Finally, the authors do not explain how it is possible for cGAS KO fish to have a clear inflammatory phenotype at a young age along with DNA damage that not only has no effect on lifespan but is lost with age. Is this a biological phenomenon or an experimental artifact?

The inflammatory phenotype in young cGAS KO fish is modest and detectable only at the pathway level. It reflects a modest compensatory upregulation of cGAS pathway components, which we note, but downplay in the text. Its reduced visibility in older fish likely reflects:

1. Increased transcriptional noise with age (Figure 4).
2. Accumulation of age-related DNA damage masking genotype-specific effects

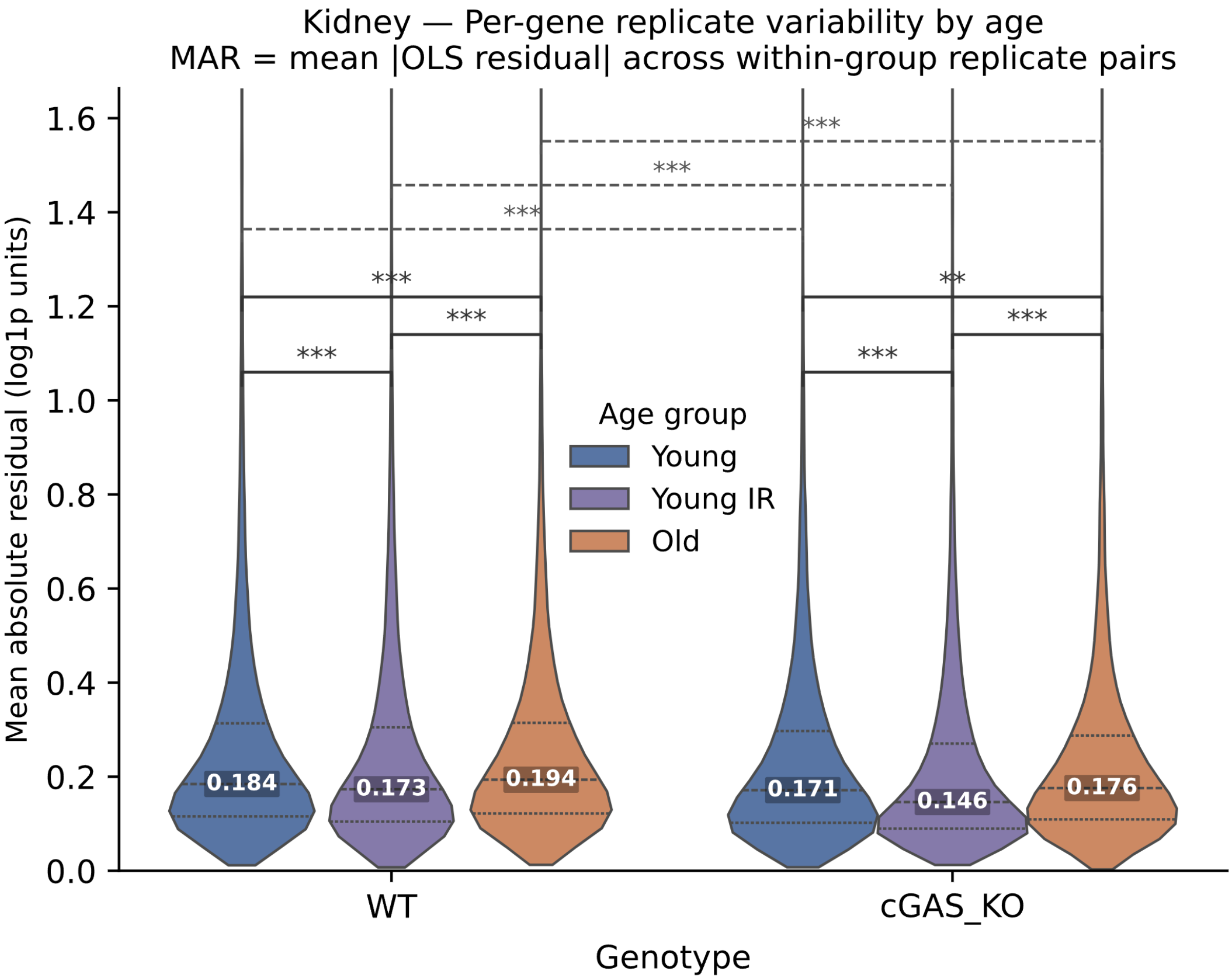

Figure 4. Variance in per gene expression significantly increases with age. The violin plots show per-gene residual distribution across biological replicates in young, young irradiated and old WT and cGAS KO kidneys.

Indeed, independent studies show that DNA damage increases in aging killifish kidneys, which could obscure early-life differences[6].

Despite this, aged kidneys show strong inflammatory activation overall, and cGAS loss specifically attenuates key components (e.g., complement pathways, SASP-related signals).

We agree that the absence of a lifespan effect is unexpected. However, we view this as a key finding rather than a limitation.

cGAS has both beneficial and detrimental roles:

- Beneficial: antiviral defense, tumor suppression
- Detrimental: promotion of senescence and chronic inflammation

Our results suggest that these opposing effects may balance at the organismal level, such that reducing cGAS-dependent inflammation does not translate into increased lifespan.

The data are not artifactual but simply defy expectations. This finding challenges a prevailing assumption in the field and underscores the importance of in vivo aging studies.

A recent article published on cGAS KO killifish in Genes & Development (https://genesdev.cshlp.org/content/early/2026/04/13/gad.352760.125) shows that cGAS KO killifish cells have disrupted H3K9me3 organization, as does a preprint on cGAS KO mice (https://pmc.ncbi.nlm.nih.gov/articles/PMC11482887/). This could help explain the rise in inflammation and gH2AX reported by the authors in their young cGAS KO fish, but not their aging data.

We agree that recent work linking cGAS to chromatin organization (e.g., H3K9me3 regulation) provides a compelling mechanistic framework. These findings are consistent with our observations of increased DNA damage in cGAS KO cells, possibly due to disrupted chro.

The aging data is weak and needs to be either expanded to include inflammation outcomes, other tissues outside of the kidney, or removed.

The phenotypic findings from young fish and in vitro are more interesting, and the authors could focus more on expanding their characterization of cGAS KO cells in fish under basal conditions as they already see a difference before irradiation.

Without these changes, the manuscript reads as contradictory and without a clear message.

We respectfully disagree that the aging data are weak or should be removed.

Our study:

- Demonstrates conserved inflammaging signatures
- Shows that cGAS modulates specific age-associated pathways

- Includes multi-tissue physiological validation

While mechanistic depth is limited, the dataset provides in vivo insight into cGAS function during normative aging, which remains scarce.

We believe the perceived contradictions arise from a misunderstanding of how inflammatory signatures are detected and interpreted across age groups. When considered in context, the data present a consistent picture: cGAS contributes to age-associated inflammation and senescence, but its loss does not extend lifespan, highlighting a complex balance between its beneficial and detrimental roles.

Arbitrating advisor #2's comments:

Overall, the study is technically solid and presents a consistent set of results across in vitro and in vivo systems. The main finding that loss of cGAS reduces senescence and aging-related features but does not extend lifespan is well supported and interesting. The work also makes a useful conceptual point, as it challenges the idea that reducing cGAS/STING-driven inflammation will necessarily increase lifespan. The use of a vertebrate aging model and inclusion of lifespan data strengthen this.

We thank the advisor for their constructive and balanced assessment.

At the same time, the study is somewhat limited in mechanistic depth. Much of the evidence is based on transcriptomic and pathway-level analyses, with limited direct functional insight. In particular, the lack of effect on lifespan is not mechanistically explained and remains rather speculative. Some in vivo analyses are also restricted to specific tissues and are not always fully conclusive.

We agree that the study emphasizes organismal and physiological outcomes rather than deep mechanistic dissection. This was an intentional design choice: our goal was to assess the role of cGAS in normative vertebrate aging in vivo.

To this end, our study integrates:

- Transcriptomic profiling
- Histological analyses of senescence, proliferation, and immune infiltration
- Lifespan measurements
- Multi-tissue necropsy

We agree that the lack of lifespan extension is not mechanistically resolved. However, we consider this an important conceptual result, suggesting that modulation of cGAS/STING-driven inflammation alone is insufficient to alter organismal lifespan.

Nevertheless, I believe that the study is sufficiently robust and relevant to be considered further provided that the conclusions are kept within the limits of the data.

We are happy to revise the manuscript to ensure that all conclusions remain appropriately constrained by the data and clearly framed as physiological rather than mechanistic insights.

References

1. Costa, E.K., et al., *Multi-tissue transcriptomic aging atlas reveals predictive aging biomarkers in the killifish*. Nat Aging, 2026. **6**(3): p. 636–664.
2. Sladitschek-Martens, H.L., et al., *YAP/TAZ activity in stromal cells prevents ageing by controlling cGAS-STING*. Nature, 2022. **607**(7920): p. 790–798.
3. Sun, C., et al., *The Activation of cGAS-STING in Acute Kidney Injury*. J Inflamm Res, 2023. **16**: p. 4461–4470.
4. Gulen, M.F., et al., *cGAS-STING drives ageing-related inflammation and neurodegeneration*. Nature, 2023. **620**(7973): p. 374–380.
5. Yang, Y., et al., *cGAS-STING signaling pathway in intestinal homeostasis and diseases*. Front Immunol, 2023. **14**: p. 1239142.
6. Morabito, G., et al., *Spontaneous aging-associated inflammation and genome instability in the immune system of turquoise killifish*. Nat Aging, 2026. **6**(3): p. 665–681.
7. Li, J., et al., *Multimodal clocks of human aging*. Cell, 2026.

Dear Dr. Antebi

Thank you for the sumbission of your revised manuscript to EMBO reports. As my colleague Achim Breiling is currently out of office, I have taken over the handling of your manuscript temporarily. We have meanwhile received the report from arbitrator #1, which is copied below.

As you will see, the arbitrating referee is very positive about your study and recommends publication pending textual changes.

Browsing through the manuscript myself, I noticed a few editorial things that we need before we can proceed with the official acceptance of your study.

- Your manuscript will be published in our Reports section. To make this possible, I kindly ask you to combine the Results and Discussion section and to keep an eye on the character limit, as for short reports the revised manuscript should not exceed 27,000 characters (including spaces but excluding materials & methods and references).

- Please describe your findings in the abstract in present tense.

- Data availability: this section should exclusively refer to datasets deposited in external repositories. Therefore, please remove the following sentence: "All data supporting the findings of this study are available from the corresponding author upon request." We also need the specific URL for the dataset (PRJNA1154547).

- Please format the references in an alphabetical instead of numerical style. 'et al' needs to be used after 10 author names.

- Please provide a callout for Figure 1b.

- Please provide Appendix Table 1 either as Table EV1 or Table 1 in the manuscript text. The information in the table could also be part of the Reagents and Tools table. In either case, please update the callouts to this table accordingly.

- Please remove the Reagents and Tools table from the manuscript and upload it as a separate .docx file (file type Reagents table). Please correct the callout to the Appendix table (see above).

- Martinez et al, 2024 is a preprint. Please cite it as follows:
(preprint: Martinez et al, 2024) in the text and in the reference list add [PREPRINT] at the end of the reference.

- Please correct the nomenclature for Expanded View figures to Figure EV# in all places: source file names, titles in the system, titles of the legends, callouts in the manuscript.

- The resolution of the submitted figures is too low for blot and microscopy images. This reduction in resolution is commonly caused by converting original 16-bit TIFF files to RGB format for publication. While this is not inherently problematic, it can raise concerns about image integrity for critical readers.

To avoid any misunderstanding and to meet EMBO Press standards, we kindly ask that you:

* Resubmit the complete figure set at the captured original data resolution.

- The information on funding in the manuscript and the online submission system must be congruent. Please add the Max Planck Research School as a funder in the system.

- Please address the following points in the figure legends using either track changes or highlights for a faster check:
* Please provide the exact p values in the legends of figures 1D,E,G,L; 3E; 4E; EV-1I,M; EV-3B; EV-4D.
* Please indicate the statistical test used for data analysis in the legends of figure EV-4B.
* Please define the box plots in terms of centre, minima, maxima, and whiskers, bounds of box and percentile in the legends of figure EV-2A.
* Please provide information related to n in the legends of figures EV-6I,K,M,O.
* Please define the error bars in the legends of figures 1C-E,G,J,L; 5B,D,F; EV-1H,I,K,L,M; EV-4A,B; EV-5B,D,F,G,H,I,J,K; EV-6B-F.
* Please define the measure of center for the error bars in the legends of figures EV-6I,K,M,O.

- Source data: I did a spot check of the microscopy images you provided and noticed that for Figure 1k, the provided images appear to be zooms, while the figure panel shows a lower magnification overview. Please check.

- Finally, EMBO Reports papers are accompanied online by

A) a short (1-2 sentences) summary of the findings and their significance,
B) 2-3 bullet points highlighting key results and
C) a schematic summary figure that provides a sketch of the major findings (not a data image).
Please provide the summary figure as a separate file in PNG or JPG format at a size of 550x300 pixels (width x height). Please note that the size is rather small and that text needs to be readable at the final size. Please send us this information along with the revised manuscript.

I look forward to seeing a new revised version of your manuscript as soon as possible.

Kind regards,

Martina Rembold, PhD
Senior Editor
EMBO reports

==================

Arbitrator #1:

The authors did a good job addressing concerns from the previous round of review and listing the limitations. This study is of broad interest to the field and should be published.

My only concern is that the manuscript reads strange as there is a disconnect between the observed detrimental aspects of cGAS KO and the conclusions of the paper that cGAS attenuates age-related signatures. While the authors report that young cGAS KO fish have more DNA damage and inflammatory signaling at both baseline and after irradiation compared to WT, they mostly shrug these findings off even though they indicate that the KO fish should experience a truncated lifespan instead of an extended one (which they expected) or an equivalent one (which they report). I think the authors should highlight the interesting findings in the young cGAS KO fish more prominently in the title and Abstract of the paper.

The authors have addressed all minor editorial requests.

Dr. Adam Antebi
Max Planck Institute for Biology of Ageing
Molecular Genetics
Gleueler Str. 50a
Cologne 50931
Germany

Dear Dr. Antebi,

I am very pleased to accept your manuscript for publication in the next available issue of EMBO reports. Thank you for your contribution to our journal.

Your manuscript will be processed for publication by EMBO Press. It will be copy edited and you will receive page proofs prior to publication. Please note that you will be contacted by Springer Nature Author Services to complete licensing and payment information.

You may qualify for financial assistance for your publication charges - either via a Springer Nature fully open access agreement or an EMBO initiative. Check your eligibility: https://link.springer.com/journal/44319/how-to-publish-with-us

Should you be planning a Press Release on your article, please notify the production team via this form in order to coordinate publication and release dates: https://forms.cloud.microsoft/e/eH4A8JVP6g

If you have any questions, please do not hesitate to contact the Editorial Office. Thank you for your contribution to EMBO Reports.

Yours sincerely,

Achim Breiling
Senior Editor
EMBO Reports

>>> Please note that it is EMBO Reports policy for the transcript of the editorial process (containing referee reports and your response letter) to be published as an online supplement to each paper. If you do NOT want this, you will need to inform the Editorial Office via email immediately. More information is available here: https://link.springer.com/partners/embo-press/editorial-policies#Peer%20review

EMBO Press Author Checklist

Corresponding Author Name: Adam Antebi	
Journal Submitted to: EMBO Reports	
Manuscript Number: 2026-64379V1	

USEFUL LINKS FOR COMPLETING THIS FORM
The EMBO Journal - Author Guidelines
EMBO Reports - Author Guidelines
Molecular Systems Biology - Author Guidelines
EMBO Molecular Medicine - Author Guidelines

Reporting Checklist for Life Science Articles (updated January
This checklist is adapted from Materials Design Analysis Reporting (MDAR) Checklist for Authors. MDAR establishes a minimum set of requirements in transparent reporting in the life sciences (see Statement of Task: 10.31222/osf.io/9sm4x). Please follow the journal's guidelines in preparing your manuscript.
Please note that a copy of this checklist will be published alongside your article.

Abridged guidelines for figures
1. Data
The data shown in figures should satisfy the following conditions:
- the data were obtained and processed according to the field's best practice and are presented to reflect the results of the experiments in an accurate and unbiased manner.
- ideally, figure panels should include only measurements that are directly comparable to each other and obtained with the same assay.
- plots include clearly labeled error bars for independent experiments and sample sizes. Unless justified, error bars should not be shown for technical
- if n<5, the individual data points from each experiment should be plotted. Any statistical test employed should be justified.
- Source Data should be included to report the data underlying figures according to the guidelines set out in the authorship guidelines on Data

2. Captions
Each figure caption should contain the following information, for each panel where they are relevant:
- a specification of the experimental system investigated (eg cell line, species name).
- the assay(s) and method(s) used to carry out the reported observations and measurements.
- an explicit mention of the biological and chemical entity(ies) that are being measured.
- an explicit mention of the biological and chemical entity(ies) that are altered/varied/perturbed in a controlled manner.
- the exact sample size (n) for each experimental group/condition, given as a number, not a range;
- a description of the sample collection allowing the reader to understand whether the samples represent technical or biological replicates (including how many animals, litters, cultures, etc.).
- a statement of how many times the experiment shown was independently replicated in the laboratory.
- definitions of statistical methods and measures:
  - common tests, such as t-test (please specify whether paired vs. unpaired), simple χ2 tests, Wilcoxon and Mann-Whitney tests, can be unambiguously identified by name only, but more complex techniques should be described in the methods section;
  - are tests one-sided or two-sided?
  - are there adjustments for multiple comparisons?
  - exact statistical test results, e.g., P values = x but not P values < x;
  - definition of 'center values' as median or average;
  - definition of error bars as s.d. or s.e.m.

Please complete ALL of the questions below.
Select "Not Applicable" only when the requested information is not relevant for your study.

Materials

Newly Created Materials	Information included in the manuscript?	In which section is the information available? (Reagents and Tools Table, Materials and Methods, Figures, Data Availability Section)
New materials and reagents need to be available; do any restrictions apply?	Yes	All plasmids generated have been deposited and can be purchased from Addgene (information at Methods section). Fish mutant strains as well as primary fibroblasts are available and can be provided upon request from the authors.

Antibodies	Information included in the manuscript?	In which section is the information available? (Reagents and Tools Table, Materials and Methods, Figures, Data Availability Section)
For **antibodies** provide the following information:<br>- Commercial antibodies: RRID (if possible) or supplier name, catalogue number and or/clone number<br>- Non-commercial: RRID or citation	Yes	All antibodies used were commercial and their information can be found in the methods section.

DNA and RNA sequences	Information included in the manuscript?	In which section is the information available? (Reagents and Tools Table, Materials and Methods, Figures, Data Availability Section)
**Short novel DNA or RNA including primers, probes**: provide the sequences.	Yes	Primer information for sequencing and qPCR as well as gRNA sequences are provided at the Appendix.

Cell materials	Information included in the manuscript?	In which section is the information available? (Reagents and Tools Table, Materials and Methods, Figures, Data Availability Section)
**Cell lines:** Provide species information, strain. Provide accession number in repository **OR** supplier name, catalog number, clone number, and/**OR** RRID.	Yes	THP1 cells were obtained from Invivogen (cGAS KO #thpd-kocgas, STING KO #thpd-kostg). Information is available at Methods section.
**Primary cultures:** Provide species, strain, sex of origin, genetic modification status.	Yes	Primary killifish fibroblasts were established from 10-week old male killifish fins of each genotype (WT, cGAS KO and STING KO). Information is available at Methods section.
Report if the cell lines were recently **authenticated** (e.g., by STR profiling) and tested for mycoplasma contamination.	Yes	The primary cell cultures were verified for their mutation status by genotyping PCR and sequencing. THP1 cells were used immediately after receipt from Invivogen, who have verified them as mentioned in their Data Sheet using functional assays, PCR and DNA sequencing. All cell cultures were tested before and after the conclusion of the experiments for mycoplasma and were verified to be negative. Information is also available at Methods section.

Experimental animals	Information included in the manuscript?	In which section is the information available? (Reagents and Tools Table, Materials and Methods, Figures, Data Availability Section)
**Laboratory animals or Model organisms:** Provide species, strain, sex, age, genetic modification status. Provide accession number in repository **OR** supplier name, catalog number, clone number, **OR** RRID.	Yes	Species: Nothobranchius furzeri. Strain: GRZ-AD. Sex: Both genders. Age: For experiments; Young (7-8 weeks) and Old (18-19 weeks), for lifespan; all ages. Mutants: Wild type and cGAS KO. All information is available at Methods section.
**Animal observed in or captured from the field:** Provide species, sex, and age where possible**.**	Not Applicable	No wild animals were used in the study.
Please detail **housing and husbandry conditions**.	Yes	All information can be found at the Methods section.

Plants and microbes	Information included in the manuscript?	In which section is the information available? (Reagents and Tools Table, Materials and Methods, Figures, Data Availability Section)
**Plants:** provide species and strain, ecotype and cultivar where relevant, unique accession number if available, and source (including location for collected wild specimens).	Not Applicable	
**Microbes:** provide species and strain, unique accession number if available, and source.	Not Applicable	

Human research participants	Information included in the manuscript?	In which section is the information available? (Reagents and Tools Table, Materials and Methods, Figures, Data Availability Section)
If collected and within the bounds of privacy constraints report on age, sex and gender or ethnicity for all study participants.	Not Applicable	

Core facilities	Information included in the manuscript?	In which section is the information available? (Reagents and Tools Table, Materials and Methods, Figures, Data Availability Section)
If your work benefited from core facilities, was their service mentioned in the acknowledgments section?	Yes	Information can be found in Acknowledgments section.

Design

Study protocol	Information included in the manuscript?	In which section is the information available? (Reagents and Tools Table, Materials and Methods, Figures, Data Availability Section)
If study protocol has been **pre-registered, provide DOI in the manuscript**. For clinical trials, provide the trial registration number **OR** cite DOI.	Not Applicable	
Report the **clinical trial registration number** (at ClinicalTrials.gov or equivalent), where applicable.	Not Applicable	

Laboratory protocol	Information included in the manuscript?	In which section is the information available? (Reagents and Tools Table, Materials and Methods, Figures, Data Availability Section)
Provide DOI OR other citation details if **external detailed step-by-step protocols** are available.	Not Applicable	

Experimental study design and statistics	Information included in the manuscript?	In which section is the information available? (Reagents and Tools Table, Materials and Methods, Figures, Data Availability Section)
Include a statement about **sample size** estimate even if no statistical methods were used.	Yes	All figures, Figure legends and Methods section explicitly mention sample sizes.
Were any steps taken to minimize the effects of subjective bias when allocating animals/samples to treatment (e.g. **randomization procedure**)? If yes, have they been described?	Yes	Although not mentioned, fish husbandry due to naturally limited space in the fish system, always includes randomization of fish locations. This prevents bias on water supply proximity and minor lighting differences on fish of different genotypes or ages.
Include a statement about **blinding** even if no blinding was done.	Yes	The methods section informs readers about blinding which took place in all microscopy, and lifespan analyses.
Describe **inclusion/exclusion criteria** if samples or animals were excluded from the analysis. Were the criteria pre-established?<br><br>If sample or data points were omitted from analysis, report if this was due to attrition or intentional exclusion and provide justification.	Not Applicable	
For every figure, are **statistical tests** justified as appropriate? Do the data meet the assumptions of the tests (e.g., normal distribution)? Describe any methods used to assess it. Is there an estimate of variation within each group of data? Is the variance similar between the groups that are being statistically compared?	Yes	All figures, Figure legends and Methods section explicitly mention statistical analyses performed and the main text as well as citations also provide information justifying the statistical analyses performed.

Sample definition and in-laboratory replication	Information included in the manuscript?	In which section is the information available? (Reagents and Tools Table, Materials and Methods, Figures, Data Availability Section)
In the figure legends: state number of times the experiment was **replicated** in laboratory.	Yes	Figure legends explicitly mention how many times the experiment has been replicated (biological replicates). All raw data for all biological replicates are included in the Source Material folders.
In the figure legends: define whether data describe **technical or biological replicates**.	Yes	Figure legends also explicitly mention whether datapoints describe technical or biological replicates.

Ethics

Ethics	Information included in the manuscript?	In which section is the information available? (Reagents and Tools Table, Materials and Methods, Figures, Data Availability Section)
Studies involving **human participants**: State details of **authority granting ethics approval** (IRB or equivalent committee(s), provide reference number for approval.	Not Applicable	
Studies involving **human participants**: Include a statement confirming that **informed consent** was obtained from all subjects and that the experiments conformed to the principles set out in the WMA Declaration of Helsinki and the Department of Health and Human Services Belmont Report.	Not Applicable	
Studies involving **human participants:** For publication of **patient photos**, include a statement confirming that consent to publish was obtained.	Not Applicable	
Studies involving experimental **animals**: State details of **authority granting ethics approval** (IRB or equivalent committee(s), provide reference number for approval. Include a statement of compliance with ethical regulations.	Yes	Animal experiments were conducted in accordance with relevant guidelines and approved by the 'Landesamt für Natur, Umwelt und Verbraucherschutz Nordrhein-Westfalen' under license number 2023.A109. Information is available at Methods section.
Studies involving **specimen and field samples:** State if relevant **permits** obtained, provide details of authority approving study; if none were required, explain why.	Not Applicable	

Dual Use Research of Concern (DURC)	Information included in the manuscript?	In which section is the information available? (Reagents and Tools Table, Materials and Methods, Figures, Data Availability Section)
Could your study fall under dual use research restrictions? Please check biosecurity documents and list of **select agents and toxins** (CDC): https://www.selectagents.gov/sat/list.htm	Not Applicable	
If you used a select agent, is the security level of the lab appropriate and reported in the manuscript?	Not Applicable	
If a study is subject to dual use research of concern regulations, is the name of the **authority granting approval and reference number** for the regulatory approval provided in the manuscript?	Not Applicable	

Reporting

The MDAR framework recommends adoption of discipline-specific guidelines, established and endorsed through community initiatives. Journals have their own policy about requiring specific guidelines and recommendations to complement MDAR.

Adherence to community standards	Information included in the manuscript?	In which section is the information available? (Reagents and Tools Table, Materials and Methods, Figures, Data Availability Section)
State if relevant guidelines or checklists (e.g., **ICMJE, MIBBI, ARRIVE, PRISMA**) have been followed or provided.	Not Applicable	
For **tumor marker prognostic studies,** we recommend that you follow the **REMARK** reporting guidelines (see link list at top right). See author guidelines, under 'Reporting Guidelines'. Please confirm you have followed these guidelines.	Not Applicable	
For **phase II and III randomized controlled trials**, please refer to the **CONSORT** flow diagram (see link list at top right) and submit the CONSORT checklist (see link list at top right) with your submission. See author guidelines, under 'Reporting Guidelines'. Please confirm you have submitted this list.	Not Applicable	

Data Availability

Data availability	Information included in the manuscript?	In which section is the information available? (Reagents and Tools Table, Materials and Methods, Figures, Data Availability Section)
Have **primary datasets** been deposited according to the journal's guidelines (see 'Data Deposition' section) and the respective accession numbers provided in the Data Availability Section?	Yes	All information available at Data Availability section.
Were **human clinical and genomic datasets** deposited in a public access-controlled repository in accordance to ethical obligations to the patients and to the applicable consent agreement?	Not Applicable	
Are **computational models** that are central and integral to a study available without restrictions in a machine-readable form? Were the relevant accession numbers or links provided?	Not Applicable	
If publicly available data were reused, provide the respective **data citations in the reference list**.	Yes	Data from Costa et al. have been cited and the authors are shown in references.
