# Supplementary material for: Loss of killifish cGAS attenuates age-related signatures but does not affect organismal life span
Source: EMBO Rep. 2026 Sep 9;27(20):5723–51. doi: 10.1038/s44319-026-00917-y (PMC13694710; doi:10.1038/s44319-026-00917-y)
Supplement: Supplementary file 2 [file 44319_2026_917_MOESM7_ESM.pdf]

Expanded View Figures

Figure EV1. Killifish and human cGAS have similar tertiary structures, and kcGASa harbors cGAMP activity.

(**A**) Multiple sequence amino acid alignment of human cGAS with killifish cGASa and cGASb generated using COBALT. Identical amino acids are shown in red, non-identical in gray. NTase, Mab21, and Zn finger domains are shown above the alignment.Right, percent identity to human cGAS as measured by BLAST. (**B**) Structural alignment of the human cGAS (PDB 5VDO) with the AlphaFold 3-predicted model of killifish cGASa bound to cGAMP. Inset depicts the cGAMP-binding pocket. (**C**) Structural alignment of the human cGAS (pdb 5vdo) with the AlphaFold 3-predicted model of killifish cGASb, while bound to cGAMP. Inset depicts the cGAMP-binding pocket. (**D**) Multiple sequence amino acid alignment of cGASa and cGASb orthologs from killifish and zebrafish was generated using COBALT. Identical amino acids are shown in red, non-identical in gray. (**E**) Triangle heatmap showing pairwise percent identity between aligned cGAS proteins in (**D**), calculated using ClustalW. (**F**) Schematic of expression vectors used for transfection of 6xHis-tagged kcGASa and kcGASb into THP1 and HEK293T cells. cGAS is driven by the *Ef-1a* promoter, and GFP driven by the *Rpbsa* promoter. Plasmid maps and sequences are provided in the Reagents & Tools Table. (**G**) Western blot analysis of HEK293T cell lysates transfected with empty expression vector or with plasmids harboring 6xHis-tagged killifish cGASa, killifish cGASa KO null mutation, and killifish cGASb. Blots were probed with anti-6xHis tag and anti-GAPDH as an internal control. (**H**) LC–MS-based targeted metabolomics of 2′,3′-cGAMP from cell extracts shown in (**G**). cGAMP levels per tissue were normalized to protein concentrations measured by BCA. Statistical analysis was performed using one-way ANOVA with Tukey correction for multiple comparisons. Error bars indicate standard deviation from the mean. (**I**) qPCR analysis of interferon and interferon-stimulated genes from cell extracts of cGAS KO THP1 cells transfected with the indicated cGAS constructs. Samples are identical to those used in (**H**). Expression values normalized to GAPDH were statistically analyzed using the Student's *t* test. Exact *P* values for the comparisons shown are: For IFNa-all; Vector vs kcGASa, $P = 0.353092$; Vector vs kcGASb, $P = 0.699750$, for IFNb; Vector vs kcGASa, $P = 0.000537$; Vector vs kcGASb, $P = 0.000988$, for IFI44; Vector vs kcGASa, $P = 0.000115$; Vector vs kcGASb, $P = 0.359836$. Error bars indicate standard deviation from the mean. (**J**) Representative flow cytometry plots of live THP1 cells transfected with plasmids carrying the indicated cGAS genes. The GFP intensity on the *x* axis (FITC-A) is plotted against background noise (APC-A) on the *y* axis. GFP + -gated cells are considered successfully transfected. (**K**) Quantification of transfection efficiency derived from the GFP+ gate as shown in (**J**). Student's *t* test was used to compare hcGAS and kcGAS means. Error bars indicate standard deviation from the mean. (**L**) Geometric means of GFP intensities of the GFP+ cells shown in (**J**). Student's *t* test was used to compare hcGAS and kcGAS geometric means. Error bars indicate standard deviation from the mean. (**M**) Percent of cells with area greater than 3000 μm^2 in irradiated and non-irradiated primary killifish fibroblasts. At least 500 cells were quantified per replicate. Statistical analysis was performed using One-way ANOVA with Dunn–Šidák correction for multiple comparisons. Exact *P* values for the comparisons shown are: WT 0 Gy vs WT 10 Gy, $P = 0.000000001902642$; KO 0 Gy vs KO 10 Gy, $P = 0.015185213159004$; WT 10 Gy vs KO 10 Gy, $P = 0.000000002802417$. Error bars indicate standard deviation from the mean. (**N**) Linear regression analysis of the log2-Fold Change (Log2FC) of genes significantly regulated by cGAS during irradiation identified by gene_irradiation interaction analysis. The *x* axis represents the Log2FC between irradiated and non-irradiated WT fibroblasts, and the *y* axis represents the Log2FC between irradiated cGAS KO and non-irradiated WT fibroblasts. The solid line indicates the linear regression fit of the significantly regulated genes in gene_irradiation interaction, while the dashed line represents the reference slope = 1 ($x = y$). (**O**) KEGG pathway enrichment of the up-inhibited genes in (**N**). Genes in this category are upregulated by irradiation in WT fibroblasts, but cGAS loss significantly blunts upregulation. All significantly (FDR < 0.05) regulated KEGG pathways are shown. (**P**) KEGG pathway enrichment comparing irradiated cGAS KO to irradiated WT primary fibroblasts. All significantly (FDR < 0.05) regulated KEGG pathways are shown. Data information: Flow cytometry data in (**J–L**) represent the transfection experiments shown in Fig. 1D,E. Cell enlargement data in (**M**) represent the irradiation experiment shown in Fig. 1F,G. Experiments were performed with three independent biological replicates, $n = 3$ plates per replicate, except for HEK293T transfections in (**G**, **H**), which were performed with two independent biological replicates, and transfection of THP1 cells for immune activation in (**I**), which was performed once. Each panel shows one representative experiment. IR irradiated, FDR false discovery rate.

© The Author(s)

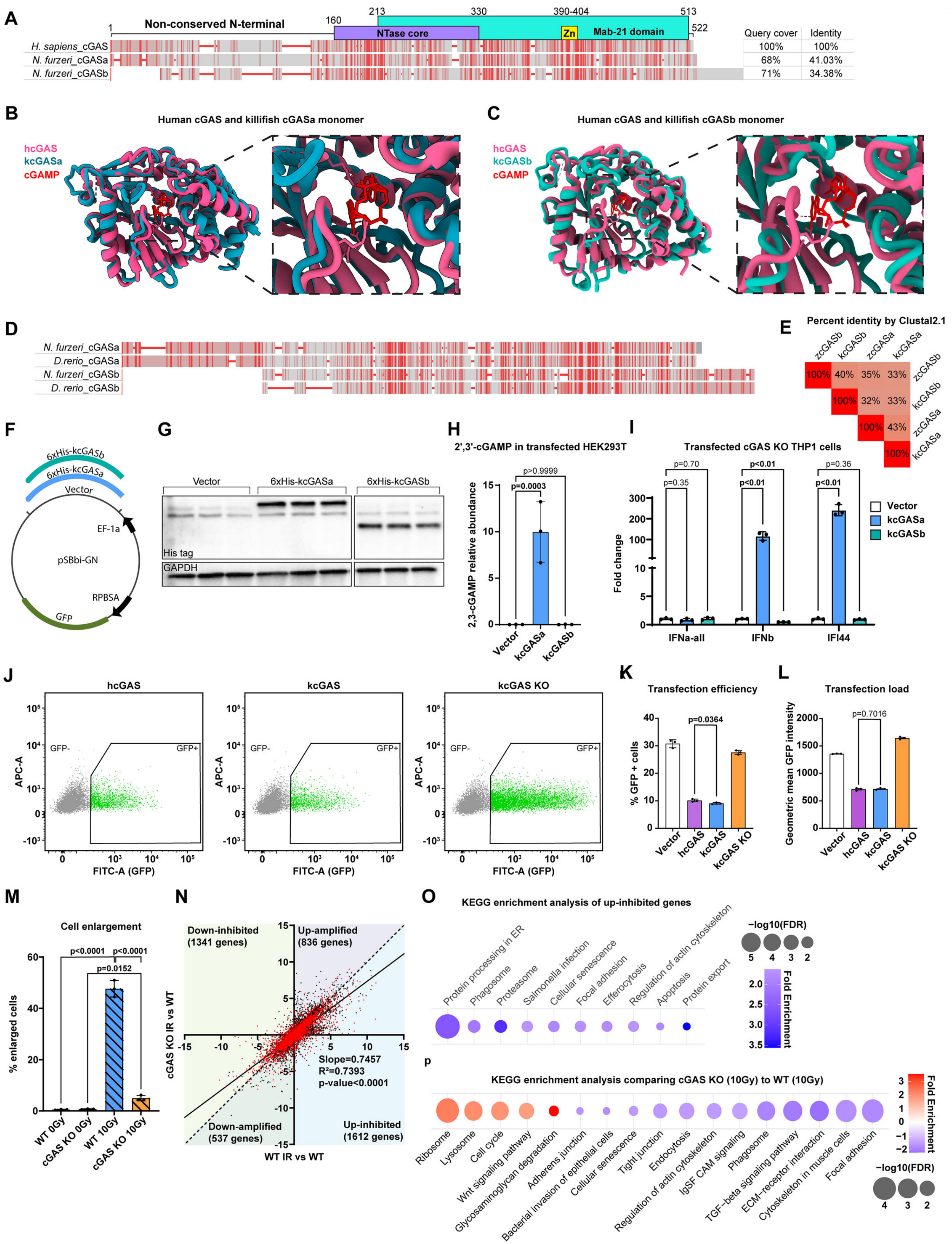

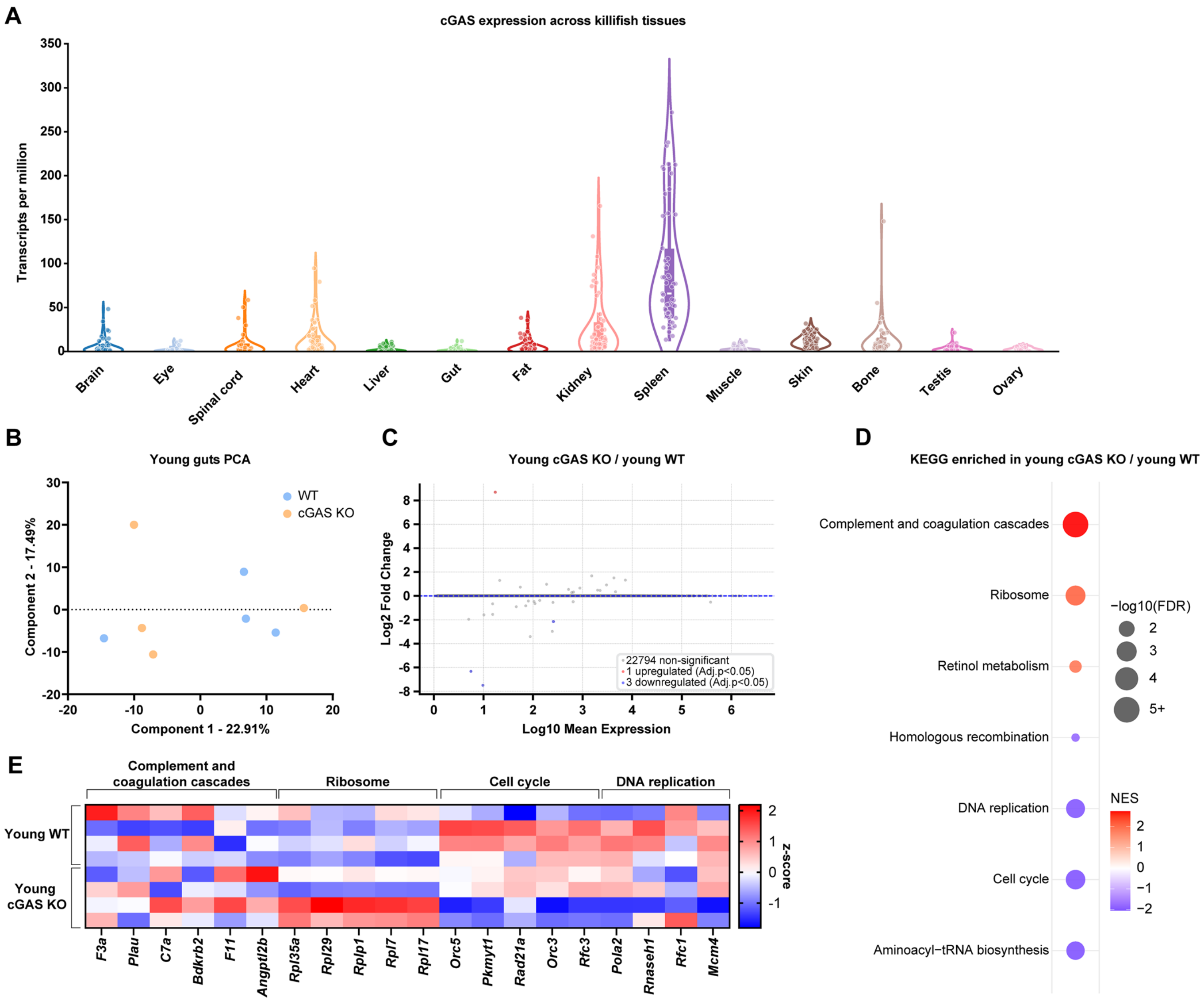
A
cGAS expression across killifish tissues
Transcripts per million
Brain
Eye
Spinal cord
Heart
Liver
Gut
Fat
Kidney
Spleen
Muscle
Skin
Bone
Testis
Ovary
B
Young guts PCA
WT
cGAS KO
Component 1 - 22.91%
Component 2 - 17.49%
C
Young cGAS KO / young WT
Log2 Fold Change
Log10 Mean Expression
22794 non-significant
1 upregulated (Adj.p<0.05)
3 downregulated (Adj.p<0.05)
D
KEGG enriched in young cGAS KO / young WT
Complement and coagulation cascades
Ribosome
Retinol metabolism
Homologous recombination
DNA replication
Cell cycle
Aminoacyl−tRNA biosynthesis
−log10(FDR)
NES
E
Complement and coagulation cascades
Ribosome
Cell cycle
DNA replication
Young WT
Young cGAS KO
z-score
F3a
Plau
C7a
Bdkrb2
F11
Angptl2b
Rpl35a
Rpl29
Rplp1
Rpl7
Rpl17
Orc5
Pkmyt1
Rad21a
Orc3
Rfc3
Pola2
Rnaseh1
Rfc1
Mcm4

 © The Author(s)

Figure EV2. Lack of cGAS minimally impacts the young gut transcriptome.

(**A**) Violin plot showing cGAS gene expression across killifish tissues. Expression data were obtained from Costa E et al (Costa et al, 2026). Exact values for each tissue are: for brain, median = 1.6245286431846302, Q1 = 1.0791615694676375, Q3 = 2.8037888982471975, IQR = 1.72462732877956, lower whisker=0.474158421926774, upper whisker = 5.390729891416537, min=0.474158421926774, max = 48.2453715667804; for eye, median=0.547680269358157, Q1 = 0.235363777736343, Q3 = 0.798345855574392, IQR = 0.562982077838049, lower whisker = 0.0, upper whisker=1.6428189723314655, min = 0.0, max = 12.1157552945876; for spinal cord, median=2.04627555126626, Q1 = 1.284645037250495, Q3 = 3.4376155205063776, IQR = 2.1529704832558827, lower whisker=0.217740281324982, upper whisker=6.6670712453902015, min = 0.217740281324982, max = 58.4880278758347; for heart, median=3.865281330013805, Q1 = 1.9589985254247875, Q3 = 12.811329526353475, IQR = 10.852331000928688, lower whisker = 0.439708512712193, upper whisker=29.089826027746508, min=0.439708512712193, max=94.5804438771201; for liver, median=0.5173101940333855, Q1 = 0.1746531946679195, Q3 = 2.0735977679541873, IQR = 1.8989445732862678, lower whisker = 0.0, upper whisker = 4.9220146278835895, min = 0.0, max = 10.8698616012988; for gut, median = 0.747865822726491, Q1 = 0.3397524591959925, Q3 = 1.795310461933685, IQR = 1.4555580027376926, lower whisker=0.0498437438811976, upper whisker=3.978647466040224, min=0.0498437438811976, max=11.8289638252401; for fat, median=3.01517811785425, Q1 = 1.73249885639874, Q3 = 5.46268366311472, IQR = 3.7301848067159797, lower whisker = 0.205501559850421, upper whisker=11.05796087318869, min = 0.205501559850421, max=38.2113117279423; for kidney, median=10.8054617219689, Q1 = 6.9128429676001595, Q3 = 27.6433653947586, IQR = 20.73052242715844, lower whisker=2.39874603857986, upper whisker=58.73914903549626, min=2.39874603857986, max=165.549858564364; for spleen, median=65.9689996898086, Q1 = 43.244477891429796, Q3 = 111.433045617879, IQR = 68.18856772644921, lower whisker = 13.3715532974671, upper whisker=213.7158972075528, min = 13.3715532974671, max = 271.947822176741; for muscle, median = 1.79864845535314, Q1 = 1.299485437079505, Q3 = 2.75280808943001, IQR = 1.453322652350505, lower whisker = 0.19207068303625, upper whisker = 4.932792067955767, min = 0.19207068303625, max=11.5862018603163; for skin, median = 11.123886655218449, Q1 = 6.916782891220315, Q3 = 16.5269339270682, IQR = 9.610151035847885, lower whisker=3.53813755557935, upper whisker=30.942160480840027, min=3.53813755557935, max=31.7439057910779; for bone, median=5.51303497371631, Q1 = 3.956933329710515, Q3 = 10.54572843574095, IQR = 6.588795106030434, lower whisker=1.2402779123645, upper whisker=20.428921094786602, min=1.2402779123645, max=148.035881907591; for testis, median=1.37947155752431, Q1 = 1.04091472081611, Q3 = 3.669744486008645, IQR = 2.6288297651925348, lower whisker=0.262387918868094, upper whisker = 7.612989133797447, min = 0.262387918868094, max = 21.4040638076684; for ovary, median=2.35800766871269, Q1 = 0.7814855668306304, Q3 = 3.962219821641135, IQR = 3.1807342548105044, lower whisker = 0.110071398308551, upper whisker=6.8056434046692, min=0.110071398308551, max = 6.8056434046692; (**B**) PCA of gut transcriptomes from young 8-week-old WT and cGAS KO animals. (**C**) MA plot showing minimal differential gene expression comparing gut transcriptomes from young cGAS KO to WT animals. Genes with adjusted P value < 0.05 were considered significantly differentially expressed and marked as upregulated (red) or downregulated (blue). (**D**) GSEA comparing gut transcriptomes from young cGAS KO to WT animals. All significantly (FDR < 0.05) up- and downregulated KEGG pathways are shown (**E**). Heatmap showing z-score-normalized expression values of genes with the highest positive and lowest negative rank metric scores from representative GSEA pathways shown in (**D**). Data information: NES normalized enrichment score, FDR false discovery rate. $n = 4$ fish per genotype.

© The Author(s)

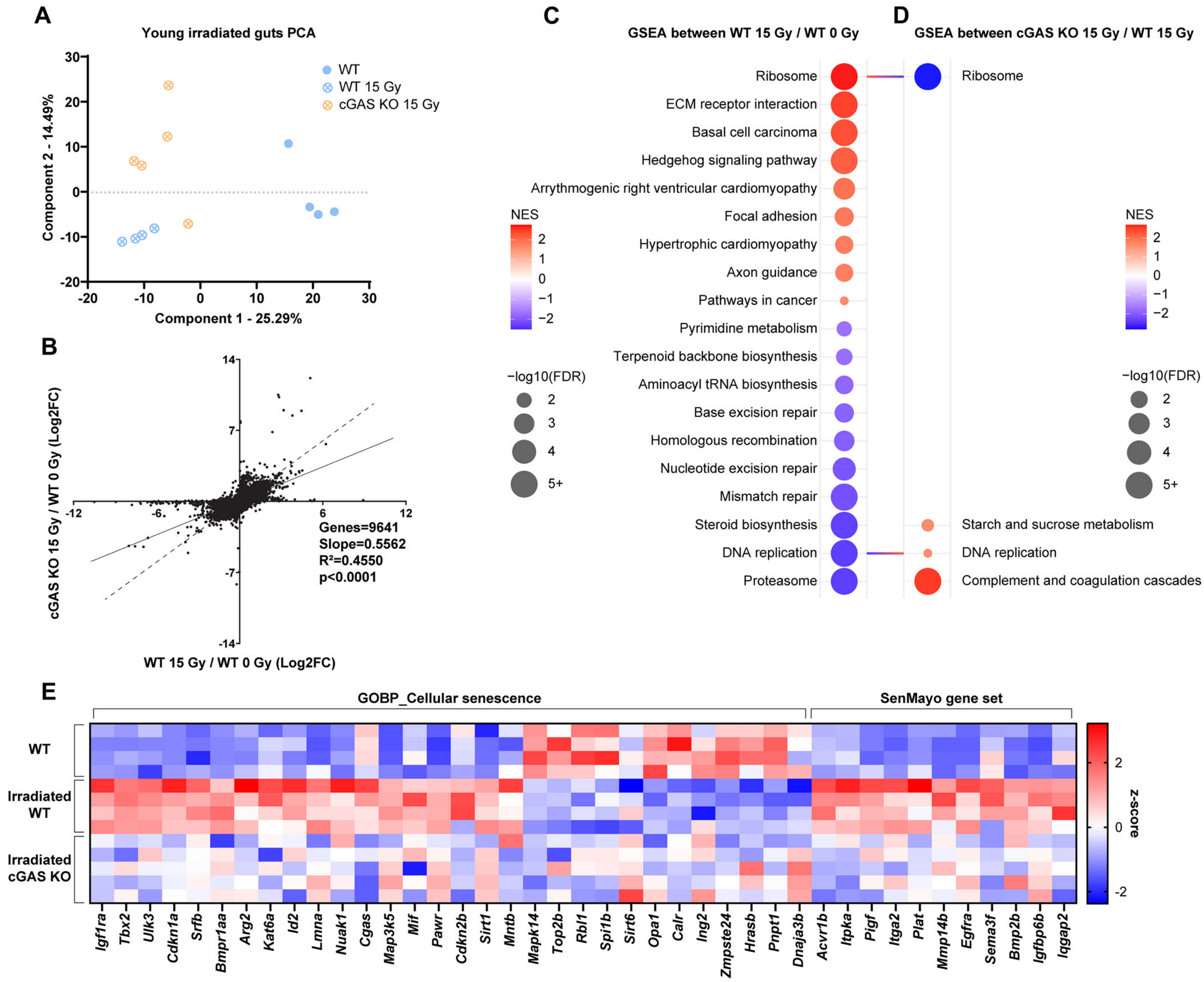

Figure EV3. cGAS modulates DNA damage-induced transcriptional changes in killifish guts.

(**A**) Principal component analysis of transcriptomes from young 8-week-old non-irradiated WT, irradiated (15 Gy) age-matched WT, and cGAS KO killifish guts. (**B**) Linear regression analysis of the Log2 Fold Change (Log2FC) of genes significantly regulated by irradiation. The *x* axis represents the Log2FC between irradiated and non-irradiated WT guts, and the *y* axis represents the Log2FC between irradiated cGAS KO and non-irradiated WT guts. Genes significantly regulated in both comparisons are shown. The solid line indicates the linear regression fit, while the dashed line represents the reference slope = 1 ($x = y$). *P* value indicates comparison between the observed slope and slope = 1, and the value is ~$3.21*10^{-1114}$. (**C**) GSEA comparing irradiated to non-irradiated WT guts. The top KEGG pathways upregulated and downregulated in irradiated WT, ranked by normalized enrichment score. (**D**) GSEA comparing irradiated cGAS KO and irradiated WT guts. KEGG pathways commonly regulated by irradiation and cGAS loss are positioned adjacent to their counterparts in (**C**), while remaining pathways are ranked independently. (**E**) Heatmap showing z-score-normalized expression values for genes with the highest positive and lowest negative rank metric scores from GSEA comparing irradiated to non-irradiated WT guts, using the SenMayo and GOBP Cellular Senescence gene sets.

 © The Author(s)

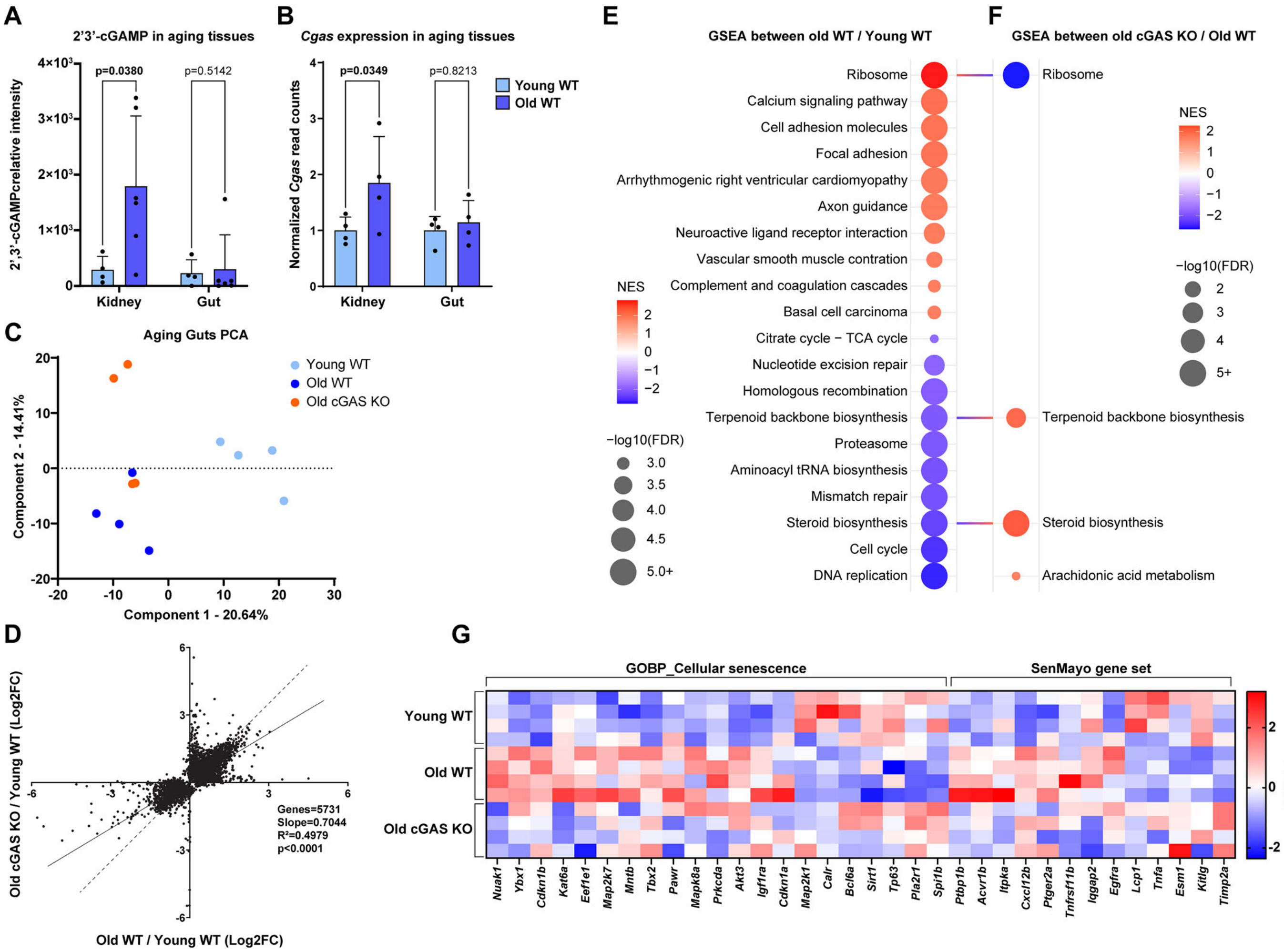

Figure EV4. cGAS modulates senescent transcriptional signatures during aging in killifish guts.

(**A**) LC–MS-based targeted metabolomics of 2′,3′-cGAMP extracted from young (7 weeks, $n = 4$) and old (18 weeks, $n = 6$) WT kidneys and guts. Statistical analysis was performed using the Mann–Whitney *U* test. Error bars indicate standard deviation from the mean. (**B**) Read counts of *Cgas* from RNAseq analysis in young and old kidneys and guts. Reads were normalized to the average of the young samples in each tissue. *P* values indicate Benjamini–Hochberg-adjusted *P* values obtained from the DESeq2 Wald test. Error bars indicate standard deviation from the mean. (**C**) PCA of gut transcriptomes from young (8 weeks) WT and old (18 weeks) WT and cGAS KO killifish. $n = 4$ for each condition. (**D**) Linear regression analysis of the Log2 Fold Change (Log2FC) of genes significantly regulated by aging. The *x* axis shows the Log2FC between old WT and young WT. The *y* axis shows the Log2FC between old cGAS KO and young WT. Genes significantly regulated in both comparisons are shown. The solid line indicates the linear regression fit, while the dashed line represents the reference slope = 1 ($x = y$). *P* value indicates comparison between the observed slope and slope = 1, and the value is ~$1.67*10^{-219}$. (**E**) GSEA comparing old to young WT guts. Top KEGG pathways upregulated and downregulated in irradiated WT, ranked by normalized enrichment score. (**F**) GSEA comparing old cGAS KO and old WT guts. KEGG pathways commonly regulated by aging and cGAS are positioned adjacent to their counterparts in (**E**), while remaining pathways are ranked independently. (**G**) Heatmap showing z-score-normalized expression values of genes with the highest positive and lowest negative rank metric scores from GSEA comparing old to young WT guts, using the Sen_Mayo and GOBP_Cellular Senescence gene sets.

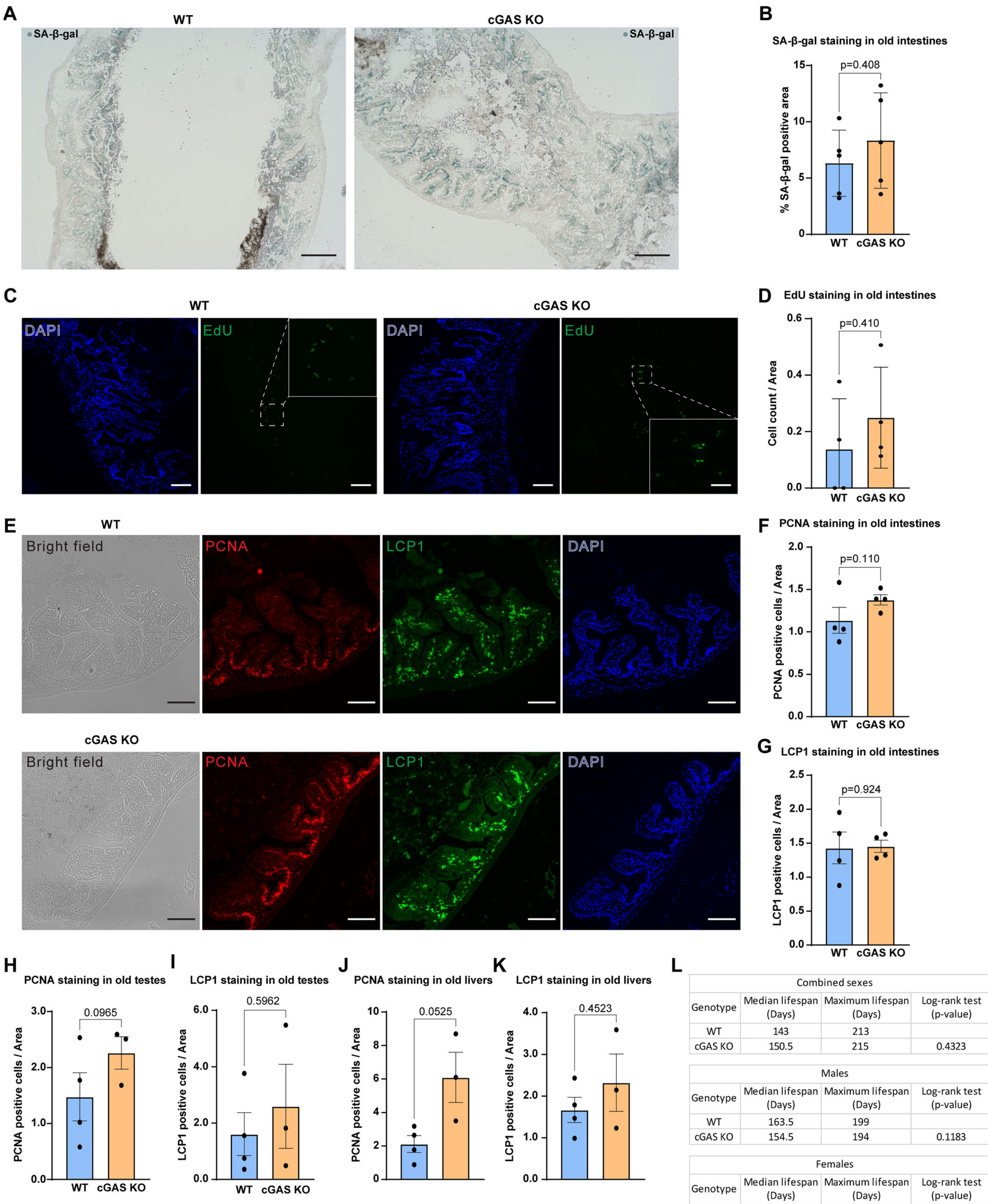

Combined sexes			
Genotype	Median lifespan (Days)	Maximum lifespan (Days)	Log-rank test (p-value)
WT	143	213	
cGAS KO	150.5	215	0.4323

Males			
Genotype	Median lifespan (Days)	Maximum lifespan (Days)	Log-rank test (p-value)
WT	163.5	199	
cGAS KO	154.5	194	0.1183

Females			
Genotype	Median lifespan (Days)	Maximum lifespan (Days)	Log-rank test (p-value)
WT	125	213	
cGAS KO	140	215	0.6738

 © The Author(s)

Figure EV5. Lack of cGAS does not significantly affect intestinal proliferation or immune infiltration in old age.

(**A**) Representative true-color bright-field images of old (19 weeks) WT and cGAS KO intestines stained for senescence-associated β-galactosidase activity. Scale bars = 200 µm. (**B**) Quantification of β-gal positive area normalized to intestinal area. Each dot represents one fish. $n = 5$ per genotype. Statistical significance was determined using the Student's *t* test. Error bars indicate standard deviation from the mean. (**C**) Representative images of old (19 weeks) WT and cGAS KO intestines stained for DAPI and EdU incorporation. EdU was injected intraperitoneally, and tissues were harvested 6 h later. Scale bars = 150 µm. (**D**) Quantification of EdU-positive cells normalized to intestinal area. Each dot represents one fish. $n = 5$ per genotype. Statistical significance was determined using the Mann–Whitney test. Error bars indicate standard deviation from the mean. (**E**) Representative Images of old (18 weeks) WT and cGAS KO intestines stained for LCP1, PCNA, and DAPI. Scale bars = 100 µm. (**F**) Quantification of PCNA-positive cells normalized to intestinal area. Each dot represents one fish. $n = 4$ per genotype. Error bars indicate standard deviation from the mean. (**G**) Quantification of LCP1-positive cells normalized to intestinal area. Each dot represents one fish. $n = 4$ per genotype. Error bars indicate standard deviation from the mean. (**H**) Quantification of PCNA-positive cells normalized to testis area. Each dot represents one fish. $n = 4$ for WT, $n = 3$ for cGAS KO. Error bars indicate standard deviation from the mean. (**I**) Quantification of LCP1-positive cells normalized to testis area. Each dot represents one fish. $n = 4$ for WT, $n = 3$ for cGAS KO. Error bars indicate standard deviation from the mean. (**J**) Quantification of PCNA-positive cells normalized to liver area. Each dot represents one fish. $n = 4$ for WT, $n = 3$ for cGAS KO. Error bars indicate standard deviation from the mean. (**K**) Quantification of LCP1-positive cells normalized to liver area. Each dot represents one fish. $n = 4$ for WT, $n = 3$ for cGAS KO. Error bars indicate standard deviation from the mean. (**L**) Tables summarizing median and maximum lifespans (in days), and *P* values calculated from Mantel–Cox log-rank statistics. Genotype and sex are indicated. Data information: At least two sections were stained per fish in (**A–K**). Statistical significance in (**F**, **G–K**) was determined using Student's *t* test.

© The Author(s)

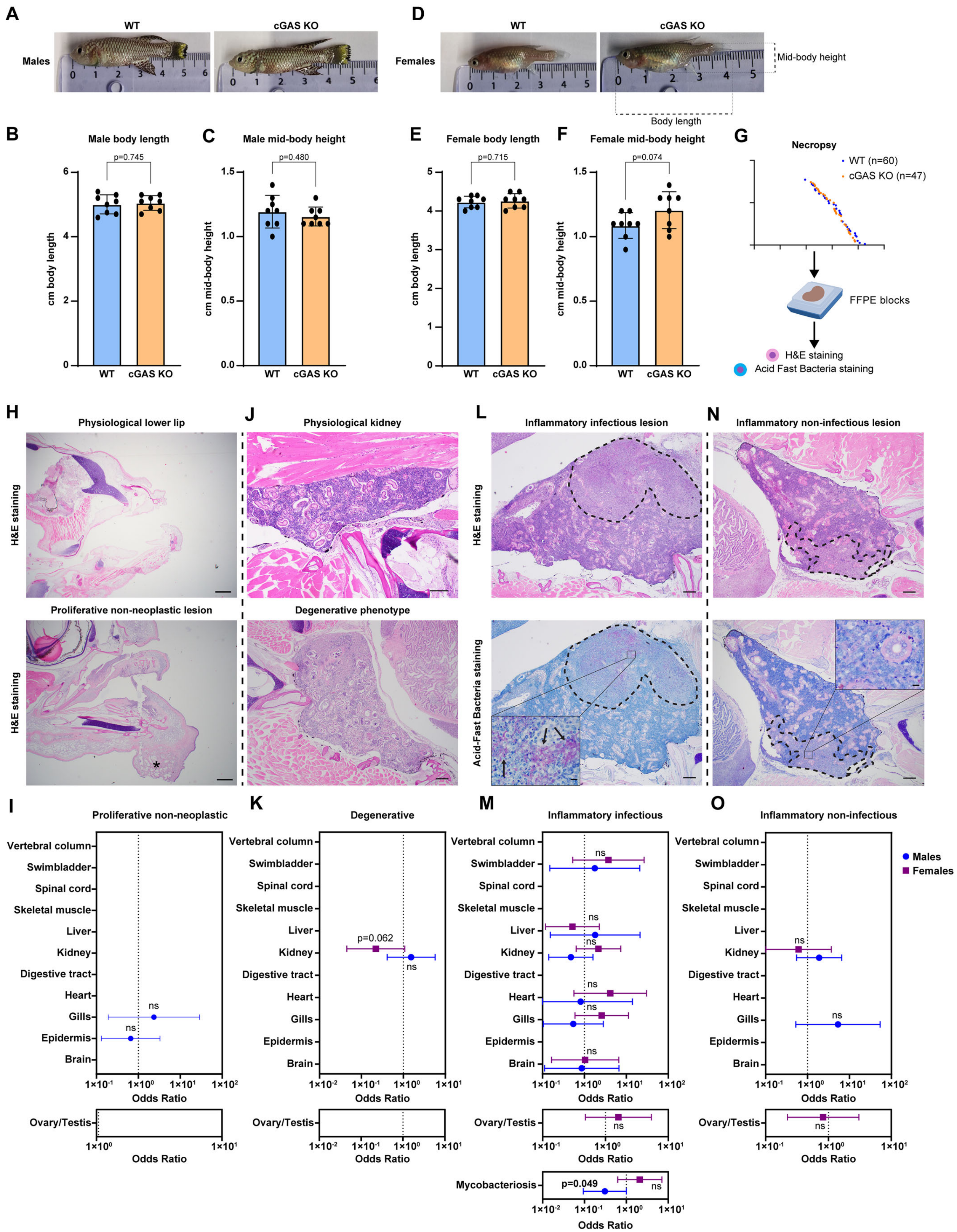
A
WT
cGAS KO
Males
D
WT
cGAS KO
Females
Mid-body height
Body length
B
Male body length
p=0.745
cm body length
WT cGAS KO
C
Male mid-body height
p=0.480
cm mid-body height
WT cGAS KO
E
Female body length
p=0.715
cm body length
WT cGAS KO
F
Female mid-body height
p=0.074
cm mid-body height
WT cGAS KO
G
Necropsy
WT (n=60)
cGAS KO (n=47)
FFPE blocks
H&E staining
Acid Fast Bacteria staining
H
Physiological lower lip
H&E staining
Proliferative non-neoplastic lesion
J
Physiological kidney
Degenerative phenotype
L
Inflammatory infectious lesion
Acid-Fast Bacteria staining
N
Inflammatory non-infectious lesion
I
Proliferative non-neoplastic
K
Degenerative
M
Inflammatory infectious
O
Inflammatory non-infectious
Vertebral column
Swimbladder
Spinal cord
Skeletal muscle
Liver
Kidney
Digestive tract
Heart
Gills
Epidermis
Brain
Odds Ratio
Ovary/Testis
Mycobacteriosis
p=0.062
p=0.049
ns
Males
Females

 © The Author(s)

Figure EV6. Killifish cGAS contributes to age-related pathology.

(**A**) Representative images showing gross male body size of old (19 weeks) WT and cGAS KO killifish. (**B**) Quantification of male body length and (**C**) mid-body height. Each dot represents one fish. Error bars indicate standard deviation from the mean. (**D**) Female gross body size in old (19 weeks) WT and cGAS KO killifish. (**E**) Quantification of female body length and (**F**) mid-body height. Each dot represents one fish. Error bars indicate standard deviation from the mean. (**G**) Schematic overview of fish retrieved for necropsy analysis from lifespan experiments (Fig. 5G–I), showing FFPE sections stained for hematoxylin & eosin (H&E) and acid-fast bacteria. (**H**) Representative H&E histological images of the jaw region depicting a normal lower lip and proliferative non-neoplastic lesion (*). (**I**) Forest plots for non-neoplastic proliferative lesions across 12 tissues in WT and cGAS KO fish. Center markers denote the point estimate of the odds ratio (OR), with error bars representing 95% confidence intervals (CI). Results summarize a multivariate logistic regression model used for interaction analysis (Pathology ~ Genotype + Sex + Genotype:Sex + Age). The OR for the female subgroup, purple square; OR for the male subgroup, a blue circle. OR and CI bars are omitted where the event incidence was zero. Statistical significance (see "Methods") is indicated adjacent to each plotted OR. (**J**) H&E histological images depicting a healthy kidney and a kidney displaying degenerative tubular changes. (**K**) Forest Plots illustrating the OR and 95% CI for degenerative pathology. Data were analyzed as in (**I**). (**L**) Representative H&E and acid-fast bacteria-stained histological images of a kidney depicting an inflammatory lesion infiltrated by mycobacteria. The outlined area contains necrosis and mononuclear inflammatory infiltrates, inset shows aggregates of acid-fast-positive rod-shaped bacteria, indicated by arrows. (**M**) Forest Plots illustrating the OR and 95% CI for inflammatory infectious lesions. Among the infectious lesions, mycobacteriosis incidence (positive for acid-fast staining) is plotted separately and indicates occurrence per fish. Data were analyzed as in (**I**). (**N**) Representative H&E and acid-fast bacteria-stained histological images of a kidney depicting an inflammatory lesion with no apparent bacterial infiltration. The inflammatory cell aggregate within the kidney is outlined. Neither positive nor negative staining acid-fast rod-shaped organisms were detected in these lesions. (**O**) Forest Plots illustrating the OR and 95% CI for inflammatory non-infectious lesions. Data were analyzed as in (**I**). Data information: For male and female size comparisons in (**A–F**), $n = 8$ fish were measured per sex and genotype. Statistical analysis was performed using Student's t test. $n = 60$ WT fish and $n = 47$ cGAS KO fish were used for necropsy in (**G–O**). 32 WT were female, and 28 were male, while 17 cGAS KO were female and 30 were male. Scalebars in kidney images = 200 µm, insets = 10 µm. Scalebars in jaw images = 500 µm.
